# Oxidase‐Type C−H/C−H Coupling Using an Isoquinoline‐Derived Organic Photocatalyst

**DOI:** 10.1002/anie.202202649

**Published:** 2022-03-19

**Authors:** Lei Zhang, Björn Pfund, Oliver S. Wenger, Xile Hu

**Affiliations:** ^1^ Laboratory of Inorganic Synthesis and Catalysis Institute of Chemical Sciences and Engineering École Polytechnique Fédérale de Lausanne (EPFL) ISIC-LSCI Lausanne Switzerland; ^2^ School of Chemistry and Material Sciences Hangzhou Institute of Advanced Study University of Chinese Academy of Sciences 1 Sub-lane Xiangshan, Hangzhou 310024 China; ^3^ Department of Chemistry University of Basel 4056 Basel Switzerland

**Keywords:** Hydrogen Atom Transfer, Minisci Reaction, Oxidation, Photocatalysis, Reaction Mechanisms

## Abstract

Oxidase‐type oxidation is an attractive strategy in organic synthesis due to the use of O_2_ as the terminal oxidant. Organic photocatalysis can effect metal‐free oxidase chemistry. Nevertheless, current methods are limited in reaction scope, possibly due to the lack of suitable photocatalysts. Here we report an isoquinoline‐derived diaryl ketone‐type photocatalyst, which has much enhanced absorption of blue and visible light compared to conventional diaryl ketones. This photocatalyst enables dehydrogenative cross‐coupling of heteroarenes with unactivated and activated alkanes as well as aldehydes using air as the oxidant. A wide range of heterocycles with various functional groups are suitable substrates. Transient absorption and excited‐state quenching experiments point to an unconventional mechanism that involves an excited state “self‐quenching” process to generate the N‐radical cation form of the sensitizer, which subsequently abstracts a hydrogen atom from the alkane substrate to yield a reactive alkyl radical.

## Graphical abstract

An isoquinoline‐derived diaryl ketone‐type photocatalyst was designed and synthesized. This photocatalyst enabled visible‐light‐mediated dehydrogenative cross‐coupling of heteroarenes with alkanes and aldehydes using air as the sole oxidant. An unconventional mechanism with “self‐quenching” to generate an isoquinoline *N*‐radical cation to trigger hydrogen atom transfer (HAT) is involved, according to time‐resolved optical spectroscopic and mass spectrometry studies.

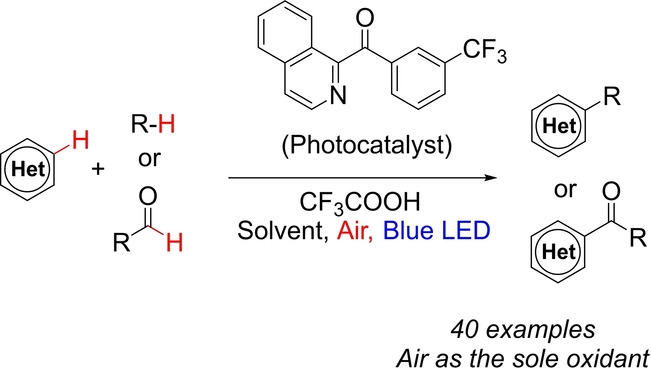

## Introduction

In nature, oxidase uses molecular oxygen (O_2_) as a proton and electron acceptor for the oxidation of organic substrates, generating water or H_2_O_2_ as a byproduct.^[1]^ Oxidase‐type oxidation methods are attractive for organic synthesis because O_2_ is an environmentally friendly and economical oxidant.^[1a]^ Although O_2_ is a thermodynamically strong oxidant, the triplet ground state of O_2_ poses substantial kinetic barriers to O_2_ reduction.^[2]^ Developments in transition metal‐catalyzed oxidase chemistry, particularly those based on Pd[^1a^, ^2^] and Cu,[^2^, ^3^] have resulted in a number of efficient aerobic C−H oxidation methods. Considering the cost, availability, and potential toxicity of many metal ions, however, metal‐free oxidase chemistry is an attractive alternative to its metal‐catalyzed counterpart.^[4]^

**Figure 1:**
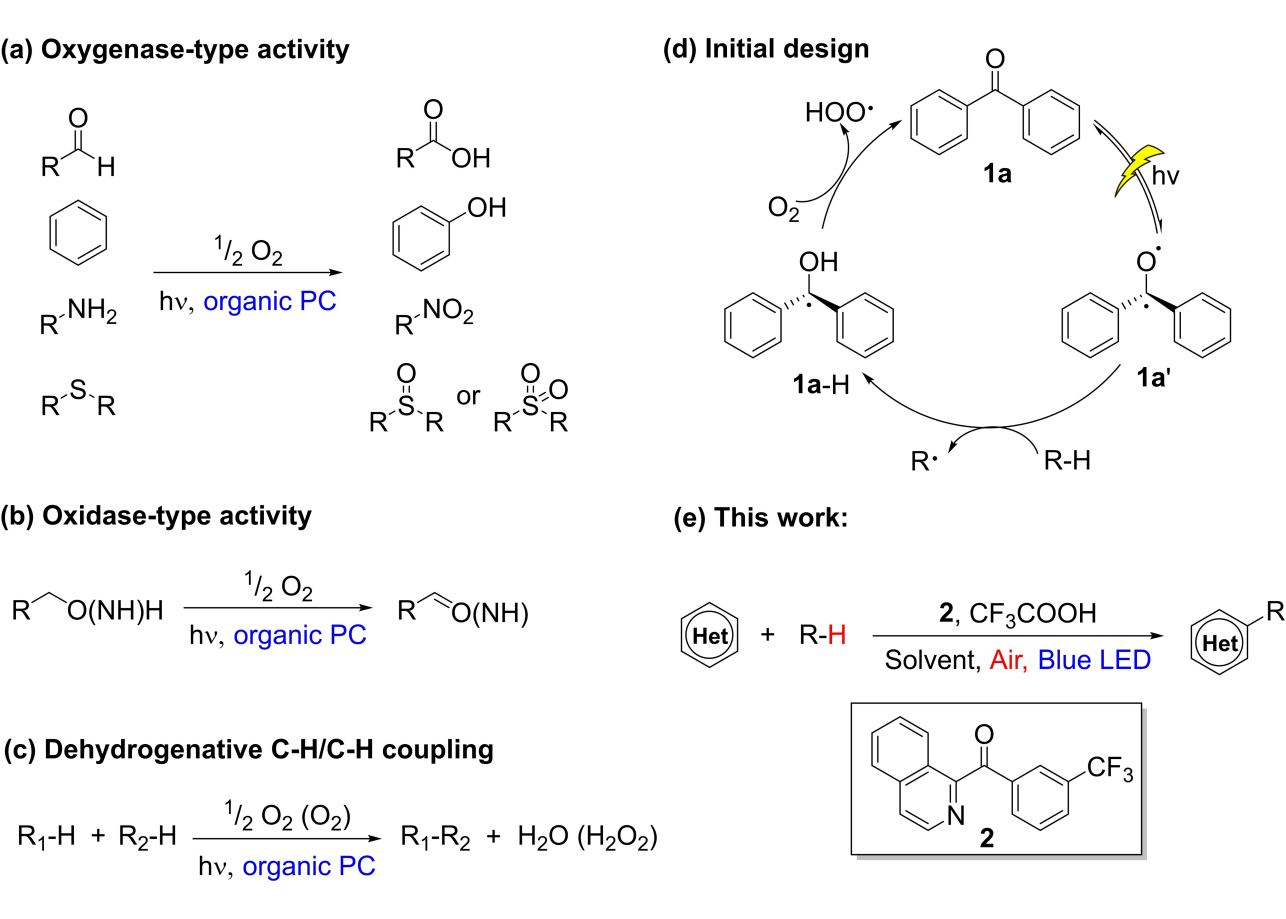
a) Oxygenase‐type activity enabled by organic photocatalysis. b) Oxidase‐type activity enabled by organic photocatalysis. c) Desired reaction: aerobic dehydrogenative C−H/C−H coupling via organic photocatalysis. d) Our initial design of the catalytic cycle. e) This work: C−H/C−H coupling using an isoquinoline‐derived organic photocatalyst (**2**).

Organic photocatalysis^[5]^ has emerged as a promising approach to move metal‐free aerobic oxidation chemistry beyond simple auto‐oxidation.^[4]^ Nevertheless, reported examples mostly describe oxygenase‐type activity (Figure 1a), where the oxygen atom(s) from O_2_ is incorporated into the organic products. Oxidase‐type activity is largely limited to oxidation of alcohols to aldehydes and oxidation amines to imines (Figure 1b).^[5a]^ Aerobic dehydrogenative C−H/C−H coupling, an attractive synthetic method which bypasses pre‐functionalization of starting reagents resulting in high atom‐ and step‐economies,^[6]^ has not been extensively studied using an organic photocatalyst under visible light (Figure 1c).^[7]^ These reactions are expected to be problematic because O_2_ can quench the excited state of photocatalysts or react with radical intermediates forming undesirable side products.

We initially considered diaryl ketones (**1 a**) as possible organic photocatalysts for oxidase‐type chemistry (Figure 1d). Diaryl ketones have a long‐lived triplet excited state, which behaves as a 1,2‐diradical and can undergo energy transfer (ET), single electron transfer (SET), or hydrogen atom transfer (HAT).^[8]^ We envisioned that an excited diaryl ketone (**1 a′**) would abstract a hydrogen atom from an alkane to form an alkyl radical and a ketyl radical (**1 a**‐H). The latter could react with O_2_ via HAT to regenerate the initial diaryl ketone catalyst (**1 a**) and produce an OOH radical (Figure 1d). Meanwhile, the alkyl radical can add to a heteroarene to form a new C−C bond. Oxidation of the resulting radical by OOH⋅ with a concomitant proton transfer completes the Minisci‐type C−H/C−H coupling^[9]^ and furnishes the H_2_O_2_ byproduct. While Minisci reactions between heteroarenes and unactivated C−H bonds have been achieved using Selectfluor,^[10]^ hypervalent iodine,^[11]^ peroxide,^[12]^ and anode^[13]^ as oxidants, an aerobic process as illustrated here remains elusive. Previously reported Minisci‐type reactions with O_2_ as the oxidant were generally limited to substrates with an activated C−H bond.[^7^, ^14^] Furthermore, we targeted air as the source of O_2_ for benefits in safety and practicality.

High‐energy UV irradiation is typically required to excite common diaryl ketones,^[15]^ which limits their applications in photoredox catalysis. We designed a new photocatalyst (**2**) where one aryl group in the diaryl ketone moiety was replaced by an isoquinoline group, and the other aryl group bore a CF_3_ group (Figure 1e). The isoquinoline group was expected to shift the light absorption to longer wavelengths due to increased π‐conjugation compared to an aryl group. The electron‐withdrawing CF_3_ group was expected to facilitate HAT processes according to previous reports.^[16]^ We show here that this new catalyst enables visible‐light‐mediated C−H/C−H coupling of heteroarenes with unactivated and activated alkanes using air as the sole oxidant (Figure 1e). Mechanistic studies reveal an unexpected mechanism for the reaction.

## Results and Discussion

**Figure 2:**
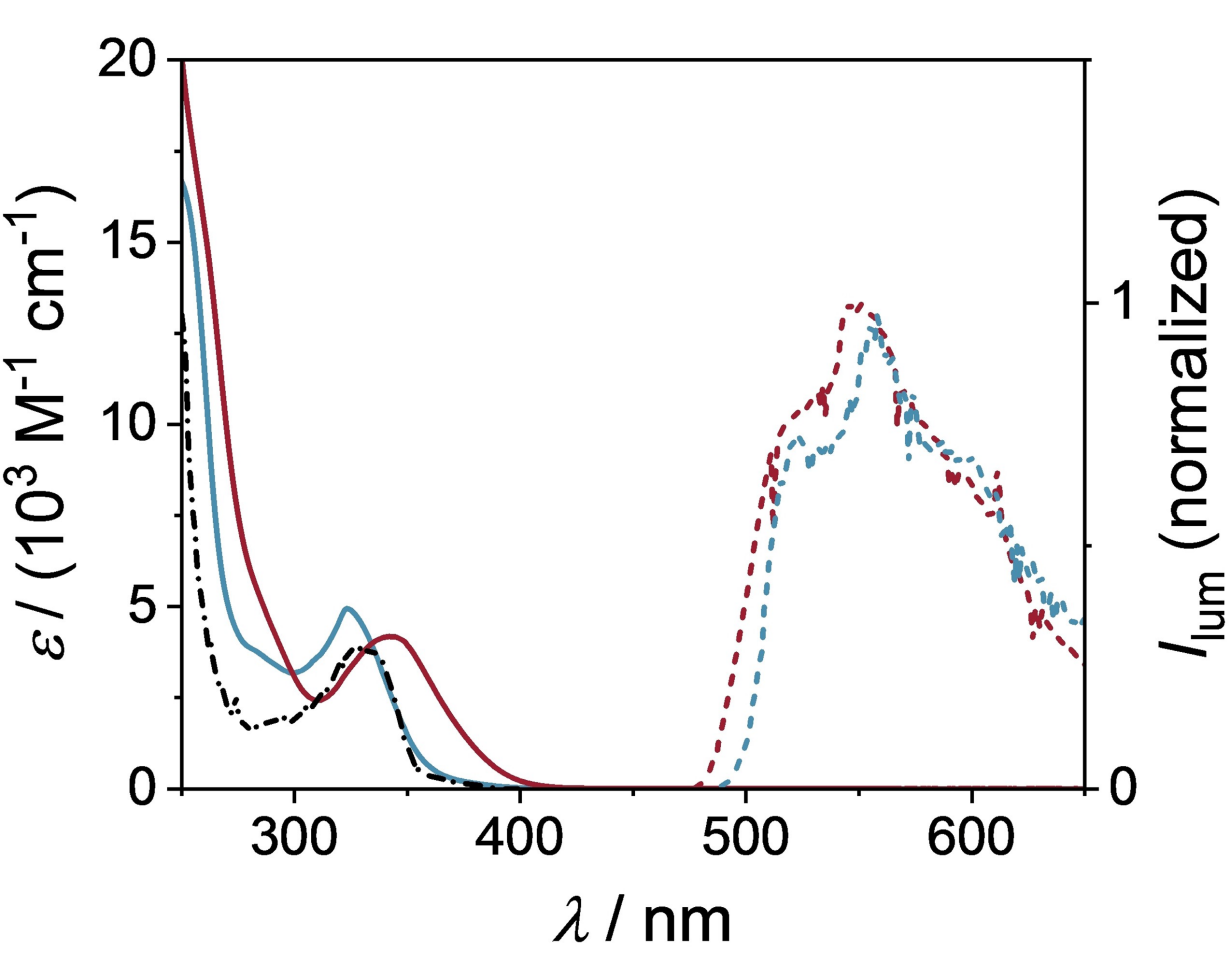
Sensitizer characteristics in the absence (**2**, blue lines) and after the addition of 20 mM CF_3_COOH (**2 H^+^**, red lines). The calibrated absorption spectra (solid lines) were recorded in CH_3_CN/CH_2_ClCH_2_Cl (2 : 1) at 20 °C. The phosphorescence spectra (dotted lines) were obtained in 2‐methyltetrahydrofuran at 77 K upon 350 nm (**2 H^+^**) and 330 nm (**2**) excitation. The excitation spectrum (black dash‐dotted line) of **2** was recorded in 2‐methyltetrahydrofuran at 77 K by monitoring the emission intensity at 550 nm as a function of excitation wavelength.

Compound **2** was synthesized from isoquinoline‐1‐carbonitrile and a newly prepared Grignard reagent (3‐(trifluoromethyl)phenyl)magnesium bromide in 86 % yield.^[17]^ The identity of **2** was confirmed by nuclear magnetic resonance (NMR) and mass spectrometry. The calibrated absorption spectra (Figure 2, solid lines) of the sensitizer in its neutral (**2**, blue line) and protonated (**2 H^+^**, red line, addition of 20 mM CF_3_COOH) forms were measured in CH_3_CN/CH_2_ClCH_2_Cl (2 : 1). The sensitizer **2** in its initial, not protonated form shows an absorption band at 325 nm with a molar extinction coefficient of 4900 M^−1^ cm^−1^. The respective absorption band of the protonated sensitizer **2 H^+^** is 20 nm red‐shifted, resulting in a band maximum at 345 nm and a slightly lower molar extinction coefficient of 4200 M^−1^ cm^−1^, tailing now into the visible. The UV/Vis absorption spectra of **2** and some representative diaryl ketones (benzophenone **1 a** and trifluoromethyl‐substituted benzophenone **1 b**) were compared in Figure S1. The result shows that the absorption of diaryl ketone compound **2** at >300 nm is greatly enhanced compared to **1 a** and **1 b**.

Phosphorescence spectra (Figure 2, dotted lines) of the charge‐neutral (**2**, dashed blue line) and protonated (**2H^+^**, dashed red line, measured after addition of 20 mM CF_3_COOH) sensitizer were obtained in 2‐methyl‐tetrahydrofuran at 77 K. The emission maxima show a minor blue shift of 10 nm from the neutral **2** (558 nm) to the protonated **2H^+^** (548 nm) sensitizer. The very large energy gap between the lowest‐energy absorption band and the emission band maximum (1.3 eV) indicates that this is indeed phosphorescence. The triplet excited state energies were estimated by determining the point on the high‐energy side of the phosphorescence band maximum at which the emission intensity amounts to 10 % compared to the maximum intensity, resulting in triplet energies of 2.45 eV (**2**) and 2.51 eV (**2 H^+^**). The excitation spectrum of the 550 nm phosphorescence of the charge‐neutral species **2** (Figure 2, black dashed‐dotted line) is very similar to the absorption spectrum (blue solid line), confirming that the emission is indeed due to the charge‐neutral sensitizer **2**.

The excited state absorption (ESA) spectra and the temporal evolution of the ESA of the neutral **2** (Figure S2a–c) and protonated **2 H^+^** (Figure S2d–f, addition of 200 mM CF_3_COOH) sensitizer were measured in CH_3_CN upon 355 nm excitation with laser pulses (12 mJ, 10 Hz). The neutral sensitizer **2** shows an ESA band at 525 nm with a shoulder at 395 nm (Figure S2a). The protonated sensitizer **2 H^+^** (Figure S2d) shows somewhat red‐shifted ESA bands at 535 nm and 402 nm. Furthermore, a ground state absorption (GSA) bleach at 360 nm was observed for **2 H^+^**, matching with the absorption band at 355 nm (Figure 2, blue solid line). The protonated sensitizer **2** shows an ESA lifetime of 340 ns under air, whereas **2 H^+^** exhibits a slightly longer lifetime of 470 ns. The same trend was observed under argon‐saturated conditions, where **2** exhibits a triplet lifetime of 9.66 μs and **2 H^+^** of 37.4 μs. When comparing the excited‐state decays of **2 H^+^** and **2** under aerobic and inert conditions, a large difference in excited state lifetime (up to a factor of 80) is observed. This drastic decrease in triplet lifetime when going from argon‐saturated to air‐saturated solution is attributable to quenching by oxygen, likely resulting in the formation of singlet oxygen.

The photocatalytic dehydrogenative coupling of methyl isoquinoline‐3‐carboxylate (**3 a**) and cyclohexane (**4 a**) using air as the oxidant under blue LED illumination was chosen as a test reaction (Table 1). After screening, we found that the C−H/C−H coupling product **5 a** was formed in 81 % GC yield using 20 mol % **2** as the photocatalyst under a set of optimized conditions (Table 1, entry 1). The heterocycle **3 a** was used as a limiting reagent on a 0.2 mmol scale, and an excess of alkane **4 a** (5 mmol, 25‐fold excess) was necessary to ensure this yield. The reaction conditions further involve CH_3_CN/CH_2_ClCH_2_Cl (3 mL, 2 : 1) as the solvent, 4 equiv of CF_3_COOH as an additive, and 24 h of reaction time at ambient temperature. Decreasing the amount of **4 a** to 10‐ and 5‐fold excess lowered the yields to 73 % and 34 %, respectively (Table 1, entries 2 and 3). CF_3_COOH was essential to the reaction. Without CF_3_COOH, no coupling was observed (Table 1, entry 4). Lowering the number of equivalents of CF_3_COOH to 2 decreased the yield to 74 % (Table 1, entry 5). Control experiments showed that illumination by blue LEDs, air, and photocatalyst **2** were all necessary for the observed high yield. Without light, no coupling occurred (Table 1, entry 6). When conducted under N_2_, the reaction gave a yield of 3 % (Table 1, entry 7), probably due to the absence of the air and a lack of photostability under these conditions, the photocatalyst could not be regenerated. Without **2**, the background reaction had a yield of 9 % (Table 1, entry 8), possibly owing to light absorption by the substrates. We also tested other organic photocatalysts. With **1 a** or **1 b**, the yield was modest (30–49 %, Table 1, entries 9 and 10), probably due to the weaker absorption of blue light (see above). With **1 c** or Eosin Y (**1 d**), nearly no coupling was observed (Table 1, entries 11 and 12). It was reported previously that in a polar solvent like CH_3_CN, the intersystem crossing to form the triplet excited‐state of **1 c** was prohibited,^[18]^ which could explain why **1 c** was not effective. Eosin Y (**1 d**) was used for aerobic oxidation of *N*‐alkylpyridinium salts.^[19]^ Its inactivity here is probably due to the inability to act as a HAT catalyst. In addition, this result excludes the possibility that reactive oxygen species generally involved in photocatalytic oxidation by Eosin Y,[^5a^, ^19^] such as singlet oxygen and superoxide, as the species that activate the unactivated C−H bonds here. Thus, the inactivity of **1 c** and **1 d** gave mechanistic insights. Slightly lower yields were obtained with CH_3_CN (Table 1, entry 13) or CH_2_ClCH_2_Cl (Table 1, entry 14) as sole solvent. However, with DMF as the solvent, the reaction only gave 3 % yield of the product (Table 1, entry 15). The substrate **3 a** appeared to react with DMF, but we could not identify the products.

**Table 1:** Summary of the influence of reaction conditions for the photocatalytic dehydrogenative coupling of **3 a** with **4 a**.

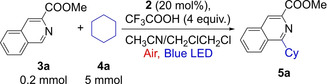		
Entry	Deviation from the standard conditions	Yield [%]^[a]^
1	none	81 (79^[b]^)
2	2 mmol cyclohexane	73
3	1 mmol cyclohexane	34
4	no CF_3_COOH	0
5	0.4 mmol CF_3_COOH	74
6	without light	0
7	under N_2_	3
8	no catalyst	9
9	**1 a** as catalyst	30
10	**1 b** as catalyst	49
11	**1 c** as catalyst	3
12	**1 d** as catalyst	2
13	CH_3_CN as the solvent	78
14	CH_2_ClCH_2_Cl as the solvent	72
15	DMF as the solvent	3
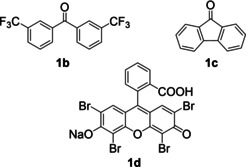		

[a] Conditions: **3 a** (0.2 mmol), **4 a** (5 mmol), **2** (20 mol %), CH_3_CN/CH_2_ClCH_2_Cl: 3 mL (2 : 1), reaction time: 24 h, cooling with fan, yield was determined by GC with mesitylene as an internal standard. [b] Isolated yield.

The above‐optimized conditions were applied to explore the scope of photocatalytic C−H/C−H coupling of heteroarenes with alkanes. We first probed the coupling of **3 a** with a variety of alkanes (Table 2). Cyclic alkanes, such as cycloheptane (**4 b**), cyclooctane (**4 c**), norbornane (**4 e**), and adamantane (**4 f**), all reacted to give good yields of coupling products (**5 a**–**5 c**, **5 e**–**5 f**). The coupling of norbornane afforded **5 e** with high *regio‐* and stereoselectivity. The coupling of a linear alkane, hexane (**4 d**) had a yield of only 30 % and a low *β*/*γ* selectivity, similar to previous reports.[^10b^, ^12b^] Unreactivated **4 d** remained in the reaction mixture. Activated alkanes were also suitable coupling partners. Moderate to high coupling yields were obtained with ethers, including THF (**4 g**), THP (**4 h**) and dioxane (**4 i**) as well as linear ethers (**4 j** and **4 k**). The coupling is regio‐selective at the C α‐to the oxygen atom. Aldehydes are also suitable coupling partners, giving acylated heterocycles in moderate to good yields (**5 l**–**5 p**). As expected, the coupling is selective at the acyl C−H bonds due to its lower bond dissociation energies compared to a typical C(sp^3^)−H bond. In these reactions, 5 equiv of aldehydes and 10 mol % of **2** were sufficient for the optimized yields. Our reaction conditions, namely ambient temperature and air as the oxidant, improved substantially over previous conditions which involved high temperature (≈100 °C) and pure O_2_ for a similar type of Minisci reactions between aldehydes and heterocycles.^[20]^ In the reaction with cyclopentanecarbaldehyde (**4 p**), 22 % of **5 p′** which originated from decarbonylation was also obtained. Similar side products were not observed or formed in a trace amount for the reactions with substrates **4 l**–**4 o**.

**Table 2:**
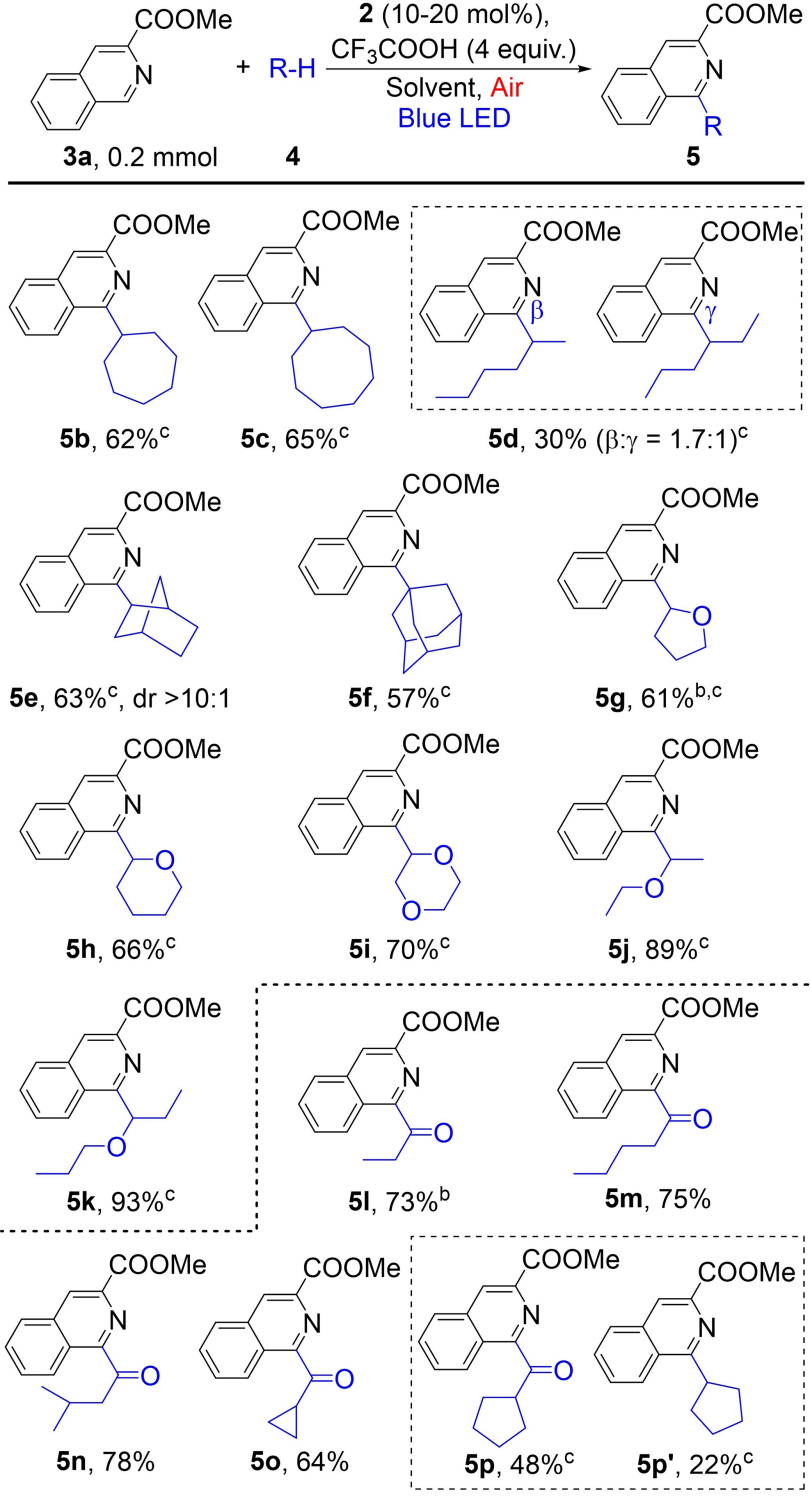
Scope of alkanes for the photocatalytic dehydrogenative coupling, using **3 a** as the heterocycle.^[a]^

[a] Conditions: **3 a** (0.2 mmol), **4** (5 mmol), **2** (20 mol %), EA: 2 mL, Reaction time: 24 h, cooling with fan, isolated yield. [b] Reaction time: 15 h. [c] **4** (1 mmol), **2** (10 mol %), CH_3_CN/CH_2_ClCH_2_Cl: 3 mL (2 : 1).

The scope of heterocycles was then explored using cyclohexane as the alkane partner (Table 3). Isoquinolines (**3 a**–**3 f**) and quinolines (**3 g**–**3 o**) were generally good substrates, giving the coupling products regioselectivity (**6 a**–**6 o**). For isoquinolines, alkylation occurs always at the 1‐position, and substituents at 3‐,4‐, or 5‐position are well tolerated. For quinolones with a 2‐substituent, alkylation occurred at the 4‐position (**6 g**–**6 j**); for those with a 4‐substituent, it occurred at the 2‐position (**6 k**–**6 o**). Other heterocycles such as phenanthridine (**3 p**), benzothiazole (**3 q**), pyridine derivatives (**3 r**–**3 x**) were also viable substrates, giving alkylated products in synthetically useful yields (**6 p**–**6 x**). Alkylation of pyridines could occur in both *ortho*‐ and *para*‐positions. Exclusive regio‐selectivity was achieved when the *ortho*‐ or *para*‐positions were blocked by substituents (**6 r**–**6 t**). When both *ortho*‐positions are available, mono‐ and di‐alkylation were observed (**6 u**–**6 x′**). Various functional groups such as cyano (**6 b** and **6 u**), ester (**6 r**, **6 s** and **6 v**), amide (**6 t** and **6 w**) and bromide (**6 e**, **6 i** and **6 o**) are compatible with the coupling conditions. The reaction of pyridine, however, gave a complex mixture with mono‐ and double‐alkylated products, where the alkylation seemed to occur at three different positions. Note that the photocatalyst **2**, although also a heterocycle, was not alkylated in these reactions because the 1‐position of isoquinoline, the reactive site for radical alkylation, was blocked by the acyl group.

**Table 3:**
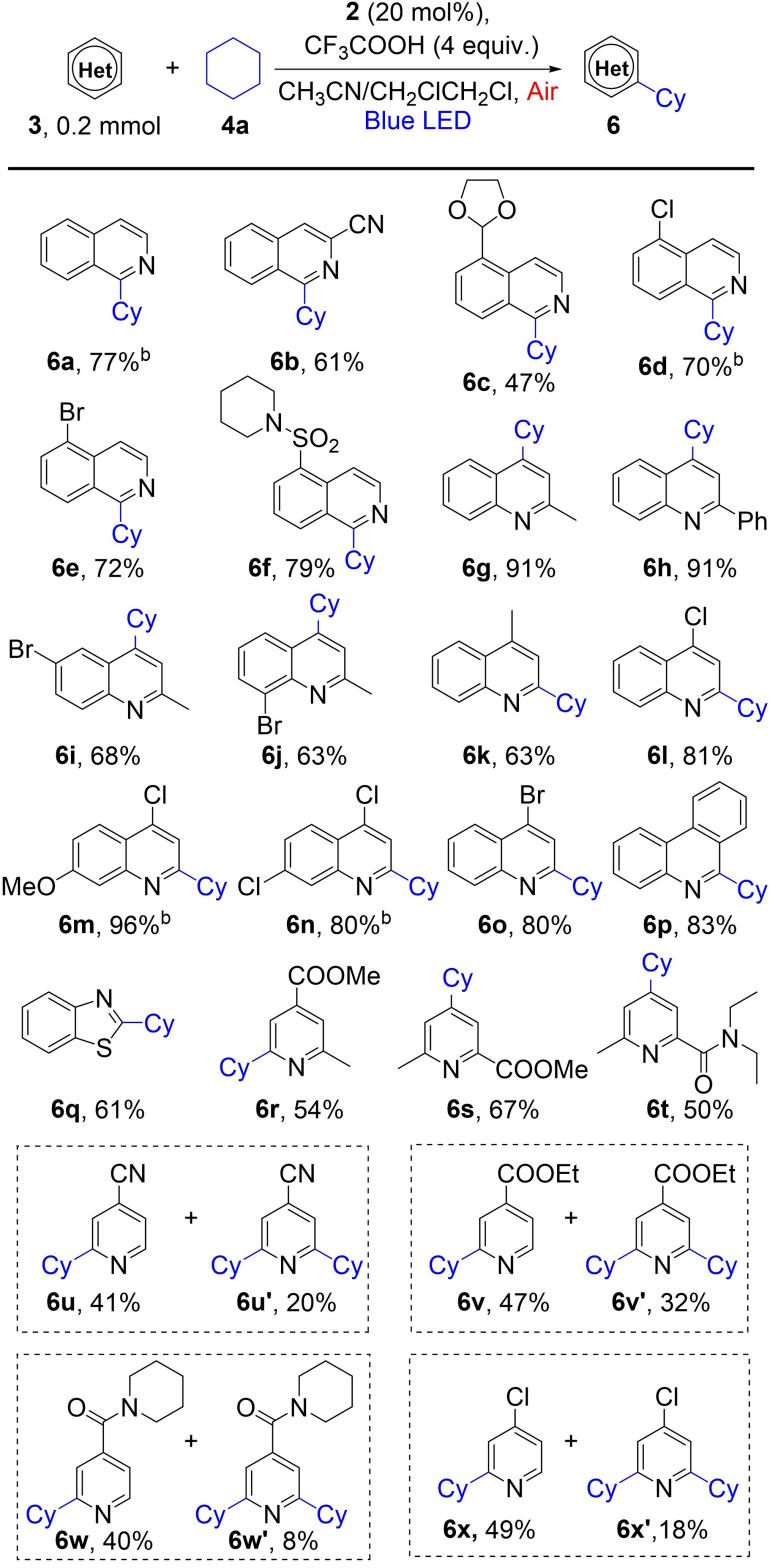
Scope of heterocycles for the photocatalytic dehydrogenative coupling, using **4 a** as the alkane substrate.^[a]^

[a] Conditions: **3** (0.2 mmol), **4 a** (5 mmol), **2** (20 mol %), CH_3_CN/CH_2_ClCH_2_Cl: 3 mL (2 : 1), reaction time: 24 h, cooling with fan, isolated yield. [b] Reaction time: 15 h.

Conceptually, the mechanism for C−H/C−H coupling can be divided into the light‐induced generation of an alkyl radical and its subsequent radical addition to the heterocycle. The second step is well investigated in literature, and is not further discussed here. However, the light‐initiated formation of the alkyl radical is less obvious and was therefore investigated in this work. Two possible pathways for the formation of the alkyl radical seemed particularly plausible to us: I) Direct hydrogen atom transfer (HAT) from the alkyl substrate (cyclohexane) to the photoexcited benzophenone sensitizer, forming an alkyl radical and the ketyl radical form of the sensitizer (Figure 1d). II) A “self‐quenching” process of the sensitizer forming the electrophilic *N*‐radical cation followed by a HAT reaction of the *N*‐radical cation with alkyl substrates.^[21]^ To investigate these two possible mechanisms, optical spectroscopy and atmospheric pressure photoionization (APPI) mass spectrometry experiments were performed.

### Excited State Quenching Experiments with Cyclohexane

**Figure 3:**
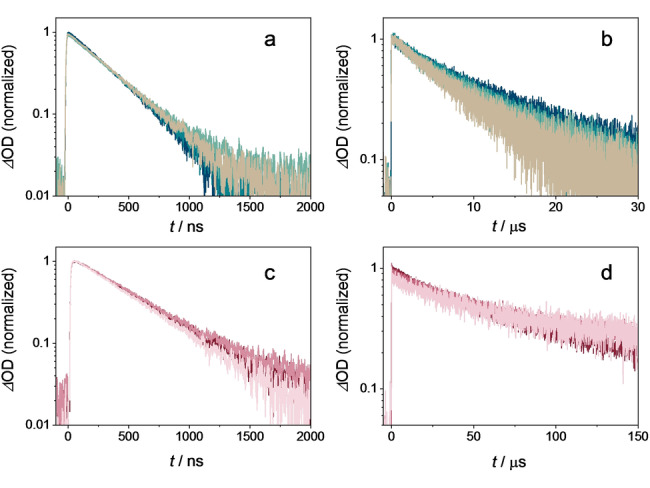
Excited state absorption decays monitored at 530 nm of **2** (a and b, 300 μM) and **2 H^+^** (c and d, 100 μM containing 200 mM CF_3_COOH), recorded in CH_3_CN at 20 °C after 355 nm pulsed excitation (12 mJ per pulse). The ESA lifetimes in panels a and c were recorded under aerobic conditions, whereas the data in panels b and d were obtained under argon‐saturated conditions with different concentrations of cyclohexane (370, 740 and 1100 mM).

In order to investigate the possibility of a direct HAT from cyclohexane to photoexcited **2** and **2 H^+^** (following addition of 200 mM of CF_3_COOH), quenching experiments of the respective photosensitizer triplet excited states were performed. To mimic the synthetically relevant reaction conditions as closely as possible, the quenching studies were first performed in CH_3_CN under aerobic conditions (Figure 3a, c) with up to 1100 mM cyclohexane (**4 a**). However, even at this very high concentration, cyclohexane is unable to quench the excited states of **2** and **2 H^+^**, indicating that direct HAT with cyclohexane is kinetically not competitive with the inherent excited‐state decay rates under these conditions. The same experiment was repeated under argon‐saturated conditions (Figure 3b, d). Analogously, no significant lifetime quenching of the excited state of the sensitizer was detectable.

Kinetic transient absorption measurements such as those in Figure 3 are typically associated with an experimental uncertainty of 10 %. In other words, pseudo first‐order reaction rate constants (1/*τ*_err_) exceeding the inherent excited‐state decay rate constant (1/*τ*_0_) by 10 % or more are detectable, whilst smaller reaction rate constants are not measurable. This leads to Equation (1) for the upper limit of the rate constant *k*_q_ for the bimolecular reaction between the triplet‐excited photosensitizer and the substrate, where [*Q*_max_] is the highest concentration of the substrate used.

(1) $$k_{q}\leq \frac{1}{\left[Q_{max}\right]}\times \;\left(\frac{1}{\tau_{err}}-\frac{1}{\tau_{0}}\right)$$

Using as input values *τ*_0_=37.4 μs (experimentally determined value in Figure 3d), 1/*τ*_err_=1.1×(1/*τ*_0_) (10 % faster decay than inherent excited‐state decay), and [*Q*_max_]=1.1 M (highest cyclohexane concentration used), one obtains an upper limit of 2.4×10^3^ M^−1^ s^−1^ for *k*_q_. In other words, the rate constant for the bimolecular reaction between triplet‐excited **2 H^+^** and cyclohexane must be slower than 2.4×10^3^ M^−1^ s^−1^.

### Excited State Quenching Experiments with Oxygen

As mentioned above, oxygen exhibits a good quenching ability for both, neutral **2** and protonated **2 H^+^** sensitizer. The reaction rate constant with oxygen was estimated from the oxygen solubility in CH_3_CN under atmospheric pressure (2.4 mM)^[22]^ and the lifetime quenching from 9.66 μs to 340 ns for **2**, and from 37.4 μs to 470 ns for **2 H^+^** (Figure S2). Using Equation 2, the bimolecular rate constants for reaction of triplet‐excited photosensitizer with oxygen (*k*_q_) are calculated as 1.2×10^9^ M^−1^ s^−1^ for **2** and 8.7×10^8^ M^−1^ s^−1^ for **2 H^+^**. These values approach the diffusion limit of 1.9×10^10^ M^−1^ s^−1^ in CH_3_CN at 25 °C.^[23]^

(2) $$k_{q}=\frac{1}{\left[O_{2}\right]}\times \;\left(\frac{1}{\tau }-\frac{1}{\tau_{0}}\right)$$

For the synthetically most relevant **2 H^+^** species, the lifetime reduction from 37.4 μs in argon‐saturated solution to 470 ns in aerated solution implies an efficiency of 98.8 % (=(470 ns)^−1^/[(37.4 μs)^−1^+(470 ns)^−1^]×100 %) for triplet excited‐state quenching by oxygen (using the excited‐state lifetime under argon‐saturated conditions as a reference point).

Given the upper limit of 2.4×10^3^ M^−1^ s^−1^ estimated above for the bimolecular reaction between triplet‐excited **2 H^+^** and cyclohexane (Equation 1), we estimate that under the synthetically relevant conditions with 1.4 M cyclohexane present, the upper limit for the pseudo first‐order rate constant for the reaction with triplet‐excited **2 H^+^** and cyclohexane is 2.4×10^3^ M^−1^ s^−1^ ×1.4 M=3.4×10^3^ s^−1^. The decay rate constant of triplet‐excited **2 H^+^** in air‐saturated CH_3_CN is 2.13×10^6^ s^−1^ (=470 ns^−1^). Consequently, the upper limit for the efficiency of direct reaction between triplet excited **2 H^+^** and cyclohexane under these conditions is 0.16 % (=(3.4×10^3^ s^−1^/2.13×10^6^ s^−1^)×100 %). As noted above, this estimate of the upper limit relies on the assumption that the transient absorption experiments in Figures 3 and S2 are suitable to detect substrate‐induced triplet decay accelerations of 10 %. This is a reasonable assumption given the sensitivity of the employed setup and the microsecond decay kinetics involved here.

### Excited State Quenching Experiments with Isoquinoline Substrate

**Figure 4:**
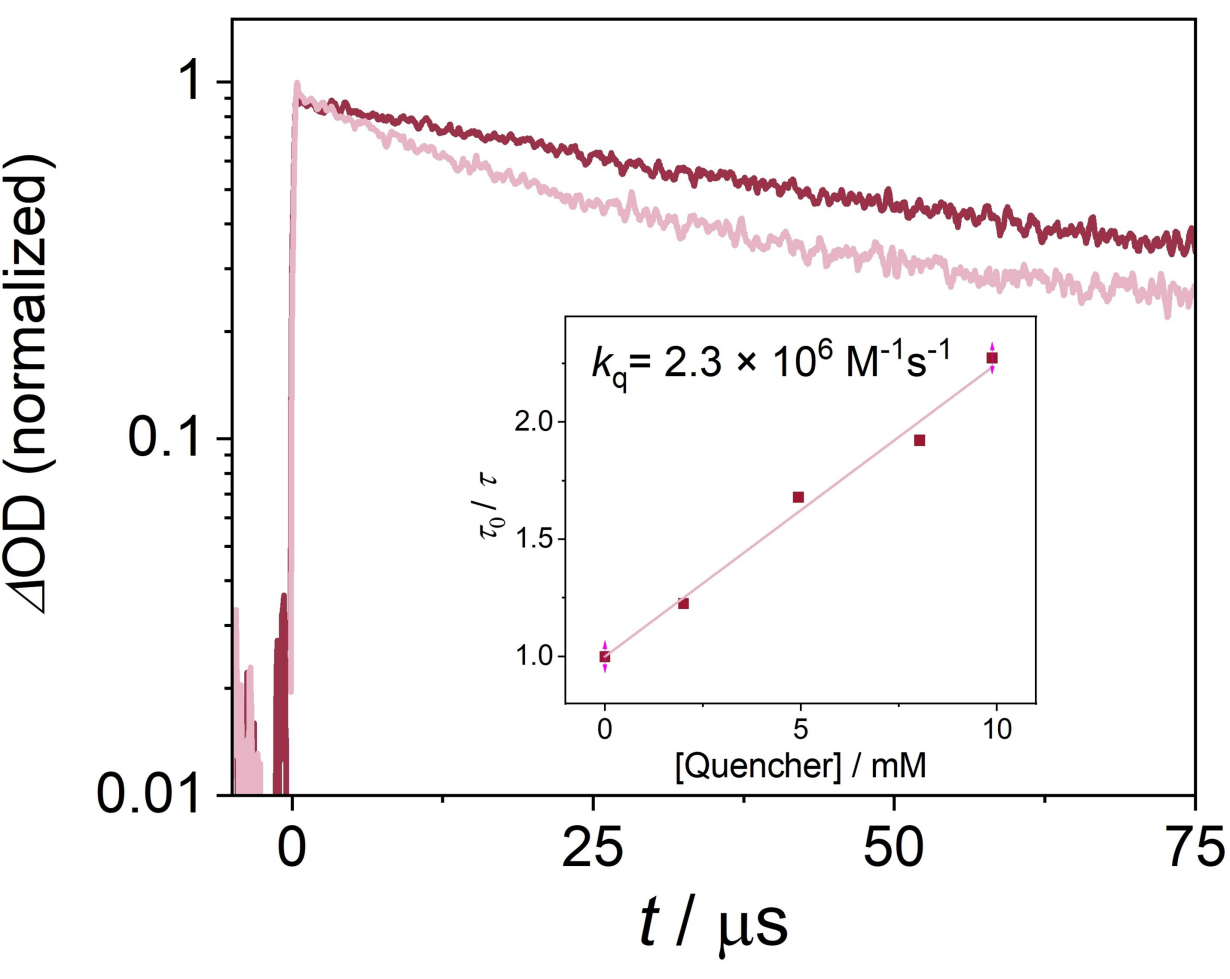
Main plot, excited state absorption decay of **2 H^+^** (1.9 mM) in argon‐saturated CH_3_CN in the absence (red line) and in the presence of 10 mM **3 a** (pale red line) upon 410 nm pulsed excitation (6 mJ per pulse). Inset: Stern–Volmer plot resulting in a reaction rate constant of 2.3×10^6^ M^−1^ s^−1^.

Based on cyclic voltammetric data, **2 H^+^** has a reduction potential of −0.33 V vs SCE in acetonitrile (Figure S6). Given an energy of 2.51 eV for its triplet state, an excited state reduction potential of 2.18 V vs. SCE is estimated based on the Rehm–Weller equation. Consequently, triplet‐excited **2 H^+^** is thermodynamically competent to oxidize the protonated isoquinoline substrate **3 a** (ca. 1.94 V vs. SCE) to its *N*‐radical cation form under the synthetically relevant reaction conditions. Experimentally, the excited state quenching of the protonated sensitizer **2 H^+^** by **3 a** (quencher) was examined by transient absorption spectroscopy (Figure 4). Since this particular quencher absorbs down to wavelengths near 360 nm, a comparatively high photosensitizer concentration of 1.9 mM had to be used in order to ensure selective photoexcitation of **2 H^+^** at the experimentally available wavelength of 410 nm (the instrumental limit of the employed laser / OPO combination). Given the nearly diffusion‐limited quenching by oxygen (see above), these quenching experiments were performed under argon‐saturated conditions, to enhance the detection sensitivity for any isoquinoline‐induced excited‐state quenching. As seen from the data in Figure 4, **3 a** indeed quenches the triplet excited state of **2 H^+^**. Based on a Stern–Volmer analysis (Figure 4, inset) the bimolecular reaction rate constant is 2.3×10^6^ M^−1^ s^−1^.

Under the synthetically relevant aerated conditions, in which 1.4 M cyclohexane and 57 mM **3 a** are simultaneously present, the pseudo first‐order rate constants for the reaction of triplet‐excited **2 H^+^** is below 3.4×10^3^ s^−1^ for the reaction with cyclohexane (see above) and 1.31×10^5^ s^−1^ (=2.3×10^6^ M^−1^ s^−1^×0.057 M) for the reaction with **3 a**. The reaction between triplet‐excited **2 H^+^** and **3 a** is therefore at least a factor of 38 (=1.31×10^5^ s^−1^/3.4×10^3^ s^−1^) faster than the reaction with cyclohexane.

### Excited State Self‐Quenching

**Figure 5:**
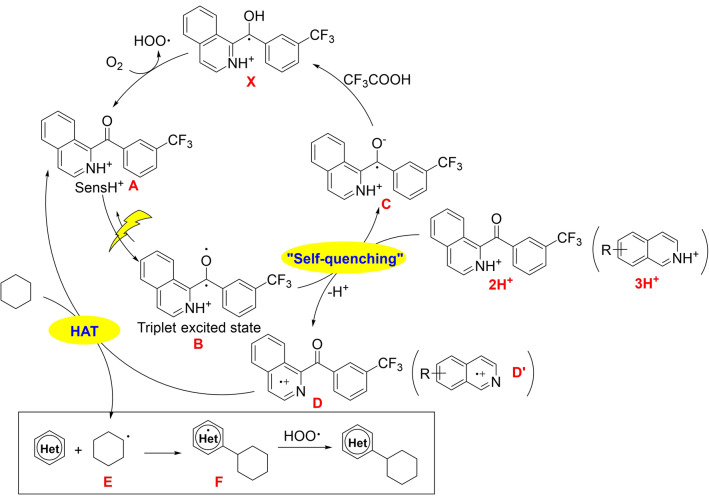
Mechanism of the photocatalytic oxidase‐type reaction described in this work. HAT=hydrogen atom transfer; Het=heterocycle.

Given the fact that the photosensitizer contains itself an isoquinoline moiety (reminiscent of substrate **3 a**), an excited state “self‐quenching” process is conceivable.^[24]^ Specifically, the excited state of **2 H^+^** (named **B** in Figure 5) could react with one ground‐state equivalent of **2 H^+^** to form the same type of *N*‐radical cation that is formed in the course of the photoinduced electron transfer reaction explored in the preceding paragraph (Figure 4).^[21b]^ In other words, triplet‐excited **2 H^+^** (**B** in Figure 5) could potentially photo‐oxidize **2 H^+^** in its electronic ground state, to yield one equivalent of species **C** and one equivalent of species **D** (Figure 5). To investigate this proposed “self‐quenching” process, excited‐state quenching experiments at different sensitizer concentrations were performed under argon‐saturated conditions (Figure S5). Upon increasing the concentration of **2 H^+^**, its excited state lifetime decreases from ≈63 μs (20 μM) to ≈13 μs (500 μM) in CH_3_CN at 20 °C. This observation seems in line with a “self‐quenching” process involving an analogous photoinduced electron transfer as for the reaction with the isoquinoline substrate **3 a** in the preceding section.

### Photocatalyst Recovery in the Catalytic Cycle

Under argon‐saturated conditions, the ESA decay traces of **2** and **2 H^+^** (Figure S2c, S2f) do not return to baseline even after very long delay times of up to 200 μs. This observation points towards the formation of a long‐lived intermediate under these specific (anaerobic) conditions. In an attempt to identify this long‐lived intermediate, transient absorption spectra with different time delays were recorded. For time delays shorter than 10 μs, the resulting ESA spectrum (Figure S3) is identical to that obtained under aerobic conditions (Figure S2d), featuring an absorption band maximum around 520 nm and a shoulder at 402 nm. At longer time delays (>50 μs), two bands at 480 nm and 522 nm arise, which resemble the published absorption spectrum of the ketyl radical of benzophenone (**1 a**–**H**), for which absorption bands at 511 nm and 540 nm are known (Figure S3).^[25]^ To further support this assignment, transient absorption spectra of **2** and **2 H^+^** in the presence of HAT‐donors (toluene, isopropanol) and an electron donor (triphenylamine) were recorded. When exciting **2** and **2 H^+^** in the presence of the abovementioned HAT‐donors, no additional ESA bands were detectable (data not shown), confirming that direct HAT to the excited sensitizer is unlikely. However, upon excitation of **2 H^+^** in the presence of excess triphenylamine (TPA) and CF_3_COOH, additional bands at 560 nm and 670 nm corresponding to TPA^+^ arise (Figure S4B).^[26]^ Furthermore, a new transient absorption band at 500 nm becomes detectable, precisely where its ketyl radical form (**1 a**‐H in Figure 1d, species **X** in Figure 5) is expected to absorb (Figure S4C). Under pH‐neutral conditions in the absence of CF_3_COOH, that ketyl radical remained undetectable on short timescales (Figure S4A). However, next to the TPA^⋅+^ signals, an additional ESA band arose at 600 nm over time, reminiscent of the spectral signature of the radical anion of **1 a**.^[25]^ Thus, under pH‐neutral conditions, the protonation of the species **C** (which is the N‐protonated form of that radical anion) to the ketyl radical (species **X** in Figure 5) is slow, but in the presence of excess CF_3_COOH it is fast. These combined observations suggest that after the formation of species **C** as a result of the abovementioned “self‐quenching” process, **C** is protonated rapidly by CF_3_COOH to yield the ketyl radical **X** (Figure 5), which corresponds to species **1 a**‐H in Figure 1d. That ketyl radical can then react with O_2_ to regenerate the initial photocatalyst form and to yield superoxide, in line with the known photoreactivity of the benzophenone archetype compound.

### Mass Spectrometry Studies

The photochemical measurements presented above exclude an efficient pathway of direct HAT from the alkyl substrate to the photosensitizer and instead point to the formation of a HAT active isoquinoline *N*‐radical cation species. A modified mass spectrometry experiment using APPI as the ionization source was adapted from literature to demonstrate the HAT ability of the isoquinoline radical cation.^[21b]^ The radical cation generated via APPI abstracts a deuterium atom (D) from isotopically labeled cyclohexane to give **2 D^+^** (Table S1), providing direct evidence for the HAT active isoquinoline *N*‐radical cation. Control experiments showed that with the non‐deuterated cyclohexane or with ESI ionization techniques (where the N‐radical cation was not formed), only protonated isoquinoline could be detected (Table S2 and S3).

### The Role of the CF_3_COOH

**Scheme 1:**
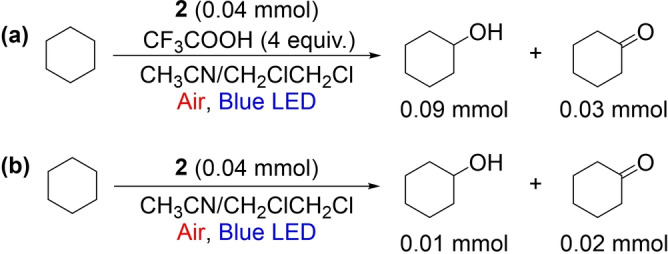
Aerobic oxidation of cyclohexane with or without CF_3_COOH under otherwise identical photochemical conditions.

CF_3_COOH was previously shown to be essential for the activation of heterocycles in Minisci‐type reactions.^[16]^ The accumulated results showed that CF_3_COOH also enhanced the activity of the photocatalyst to promote the present reaction. The addition of CF_3_COOH to **2** shifted its UV/Vis spectrum to longer wavelengths (Figure 2). Thus, one key function of CF_3_COOH is to protonate **2**, leading to better absorption of visible light. Furthermore, the observed self‐quenching process is more dominant for the protonated sensitizer (**2 H** ^+^), because in this case the remaining transient absorption signal at a delay time of 200 μs is about 3 times more intense than in the case of **2**, in line with the much faster proton transfer reaction from the radical anion of **2 a** under acidic conditions. To corroborate this hypothesis, we monitored the light‐mediated aerobic oxidation of cyclohexane with **2** as the photocatalyst (Scheme 1). The reaction was carried out under the standard conditions in the absence of a heterocycle substrate. In the presence of 4 equiv of CF_3_COOH, 0.09 mmol of cyclohexanol and 0.03 mmol of cyclohexanone were obtained. In the absence of CF_3_COOH, only 0.01 mmol of cyclohexanol and 0.02 mmol of cyclohexanone were observed. Assuming cyclohexanol and cyclohexanone were produced by quenching of a cyclohexyl radical intermediate with air, these results further indicate the higher photoactivity of **2 H** ^+^ relative to **2**.

### C−H Bond Activation of Aldehyde

Experiments were further conducted to explore the activation of aldehyde as the target C−H bond in this compound class is much weaker than in alkanes. The O_2_ itself has been reported to activate aldehydes to give acyl radicals through aldehyde auto‐oxidation.[^20^, ^27^] Therefore, O_2_ may work as a direct oxidant to generate an acyl radical. A hydroacylation reaction was carried out between fumarate **6** and aldehyde **3 n** in the presence of **2**. The reactions occurred at ambient temperature (≈30 °C) with a blue LED as the light source. When the reaction was performed in the absence of the photocatalyst, only 4 % GC yield of C−C bond formation product **7** was obtained after 12 h (Table 4, a). With the photocatalyst under N_2_ atmosphere, 40 % **7** was observed after 12 h (Table 4, b). The reaction was faster with **2** under air, and 70 % yield of **7** was obtained after 12 h (Table 4, c). These results demonstrated the essentiality of the photosensitizer, which may activate the C−H bond to trigger the reaction. Although O_2_ in the air accelerates and improves the reaction, either by regenerating the photocatalyst **2** or by activating the aldehyde directly, it is not essential for this reaction. It is worth mentioning that the present system (Table 1) is an efficient method to add aldehydes to α, β‐unsaturated esters. Previous methods for hydroacylation of α, β‐unsaturated esters through aldehyde auto‐oxidation required a very long reaction time (3–10 days) at an elevated temperature.^[27]^ By contrast, the present process occurred at ambient temperature (≈30 °C) and within a much shorter reaction time.

**Table 4:** Aerobic C−H bond activation of aldehyde.^[a]^

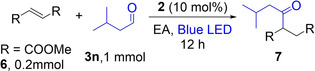		
Entry	Reaction conditions	Yield [%]
A	without **2**, under air atmosphere	4
b	with **2**, under N_2_ atmosphere	40
c	with **2**, under air atmosphere	70

[a] Conditions: **6** (0.2 mmol), **3 n** (1 mmol), **2** (10 mol %), EA: 2 mL, reaction time: 12 h, cooling with fan, yield was determined by GC with mesitylene as an internal standard.

### Proposed Mechanism

Based on the above results, we propose the mechanism of the photocatalytic dehydrogenative coupling of non‐activated alkanes as described in Figure 5. Upon irradiation, the photosensitizer **A** (**2 H^+^**) is excited to a relatively long‐lived triplet state **B**. The latter is quenched by another molecule of **2 a** or a heterocyclic substrate (with a quinoline‐like unit) to form **C** and **D (D′)**. Species **C** undergoes a fast proton transfer under acidic conditions forming the ketyl radical (**X**). The ketyl radical is regenerated by O_2_ to **A** while forming a superoxide radical. Meanwhile, species **D (D′)** abstracts a hydrogen atom from the alkane (e.g, cyclohexane) to give an alkyl radical **E**. The latter adds to the heterocyclic substrate to give intermediate **F**, which is oxidized by superoxide radical to furnish the dehydrogenative C−H/C−H coupling product.

## Conclusion

In summary, we have developed a new organic photocatalyst for oxidase‐type reactions with atmospheric oxygen as the terminal oxidant. The isoquinoline‐derived diaryl ketone **2** exhibits a long‐lived photo‐oxidizing triplet excited state, a high triplet energy (≈2.51 eV), and substantial absorption in the visible spectral range after protonation. Aerobic dehydrogenative C−H/C−H coupling between heterocycles and alkanes was achieved with **2** as the photocatalyst. Both unactivated and activated alkanes, as well as aldehydes were suitable C−H bond sources. A wide range of heterocycles with various functional groups were suitable substrates under oxidase‐type reaction conditions. Time‐resolved optical spectroscopic experiments under various conditions were performed to probe the mechanism. An unanticipated “self‐quenching” process forming the *N*‐radical cation form of isoquinoline moieties seems to be crucial for the hydrogen atom abstraction from cyclohexane, to enable the overall observable photoreactivity. This mechanism is facilitated by the photochemical properties of the new photocatalyst **2**.^[28]^

## Conflict of interest

The authors declare no conflict of interest.

1

## Supporting information

As a service to our authors and readers, this journal provides supporting information supplied by the authors. Such materials are peer reviewed and may be re‐organized for online delivery, but are not copy‐edited or typeset. Technical support issues arising from supporting information (other than missing files) should be addressed to the authors.

Supporting Information

## Data Availability

The data that support the findings of this study are available in the supplementary material of this article. The data are also accessible on Zenodo.^[28]^
