# Supplementary material for: Oxidase‐Type C−H/C−H Coupling Using an Isoquinoline‐Derived Organic Photocatalyst
Source: Angew Chem Int Ed Engl. 2022 Mar 19;61(20):e202202649. doi: 10.1002/anie.202202649 (PMC9310868; doi:10.1002/anie.202202649)
Supplement: Supplementary file 1 — Supporting Information [file ANIE-61-0-s001.pdf]

Angewandte Chemie
Eine Zeitschrift der Gesellschaft Deutscher Chemiker

Supporting Information

Oxidase-Type C–H/C–H Coupling Using an Isoquinoline-Derived Organic Photocatalyst

L. Zhang, B. Pfund, O. S. Wenger, X. Hu**

Table of Contents

1. General Information S2
2. Visible-Light-Mediated Aerobic Oxidative Cross-Dehydrogenative Coupling S3
3. UV-vis Spectra S24
4. Time Dependent Spectroscopic Measurements S24
5. Cyclic Voltammetry and Determination of Excited State Potential S30
6. Mass Spectrometry Studies S31
7. References S33
8. NMR Spectra S34

1. General Information

a. Materials

All manipulations were carried out under air unless otherwise indicated. The following chemicals were purchased and used as received: **1a**, **1b** and **1c** (TCI), Eosin Y (Aldrich), CF_3COOH (Aldrich), heteroaromatics (Aldrich, TCI or Fluorochem). **2** were prepared according to previously reported procedures.[1] All other reagents and solvents were purchased from commercial sources and used without purification.

b. Analytical Methods

NMR spectra were recorded on Bruker Avance 400 MHz spectrometers. ^{1}H NMR chemical shifts were referenced to residual protio solvent peaks or tetramethylsilane signal (0 ppm), and ^{13}C NMR chemical shifts were referenced to the solvent resonance. Data for ^{1}H NMR are recorded as follows: chemical shift (δ, ppm), multiplicity (s = singlet, d = doublet, t = triplet, m = multiplet or unresolved, coupling constant (s) in Hz, integration). Data for ^{13}C NMR are reported in terms of chemical shift (δ, ppm). GC measurements were conducted on an Agilent Technologies 7890A GC equipped with a FID detector. GC-MS measurements were conducted on an Agilent Technologies 7890A GC system equipped with a 5975C MS detector. HRMS-ESI measurements were conducted at the EPFL ISIC Mass Spectrometry Service with a Micro Mass QTOF. Room temperature absorption and 77 K phosphorescence spectra were measured using a Cary 5000 spectrometer from Varian and a Fluorolog-3-22 setup from Horiba Jobin-Yvon, respectively. The emission spectra were corrected for the wavelength-dependent sensitivity of the spectrometer.

2. Visible-Light-Mediated Aerobic Oxidative Cross-Dehydrogenative Coupling

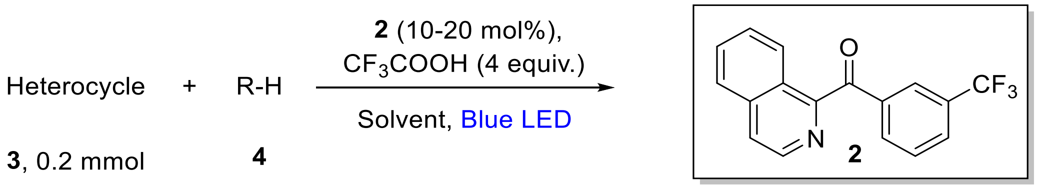

General procedure for aerobic C-H bond activation under organic photocatalysis: In the air, the photocatalysts (6 or 12.0 mg, 10-20 mol%), heterocycle (0.2 mmol), CF_3COOH (61 uL, 0.8 mmol, 4 equiv.), an R-H substrate, and solvent (CH_3CN/CH_2ClCH_2Cl = 2/1, 3 mL or ethyl acetate (EA), 2 mL) were added to a test tube equipped with a magnetic stir bar. The reaction was placed in the photoreactor cooled with a fan and stirred generally for 15~24 h. The resulting solution was extracted with ethyl acetate (3 mL x 3), after which the organic solution was combined and dried over $MgSO_4$. Then the solvent was evaporated under vacuum and the residue was purified by chromatography on silica gel, eluting with the mixture of ethyl acetate/hexane or (CH_2ClCH_2Cl/hexane) to give the corresponding products.

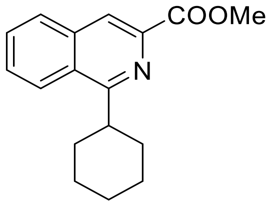

Methyl 1-cyclohexylisoquinoline-3-carboxylate (5a). 5a was synthesized following the general procedure. The residue was purified by chromatography on silica gel, eluting with hexane/ethyl acetate (5:1) to give the title compound as pale yellow solid (42.4 mg, 79%).

5a: ^{1}H NMR (400 MHz, $CDCl_3$) δ 8.39 (s, 1H), 8.30 – 8.24 (m, 1H), 7.97 – 7.91 (m, 1H), 7.75 – 7.67 (m, 2H), 4.02 (s, 3H), 3.57 (tt, *J* = 11.2, 3.9 Hz, 1H), 2.05 – 1.88 (m, 6H), 1.85 – 1.77 (m, 1H), 1.60 – 1.38 (m, 3H). ^{13}C NMR (101 MHz, $CDCl_3$) δ 166.9, 166.2, 140.8, 136.1, 130.2, 129.1, 129.1, 127.8, 125.0, 122.5, 52.7, 42.1, 32.3, 26.9, 26.1.

These spectroscopic data correspond to reported data.[2]

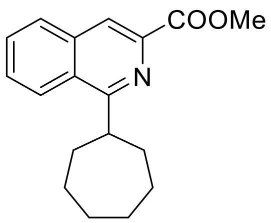

Methyl 1-cycloheptylisoquinoline-3-carboxylate (5b). 5**b** was synthesized following the general procedure. The residue was purified by chromatography on silica gel, eluting with hexane/ethyl acetate (10:1) to give the title compound as colorless oil (35.2 mg, 62%).

5b: ^{1}H NMR (400 MHz, $CDCl_3$) δ 8.37 (s, 1H), 8.26 – 8.21 (m, 1H), 7.95 – 7.89 (m, 1H), 7.73 – 7.66 (m, 2H), 4.01 (s, 3H), 3.73 (tt, *J* = 8.3, 5.3 Hz, 1H), 2.12 – 2.03 (m, 4H), 1.97 – 1.87 (m, 2H), 1.80 – 1.62 (m, 3H). ^{13}C NMR (101 MHz, $CDCl_3$) δ 167.6, 166.9, 140.6, 136.2, 130.2, 129.2, 129.1, 127.6, 125.2, 122.4, 52.8, 44.2, 34.3, 28.2, 27.7.

These spectroscopic data correspond to reported data.[2]

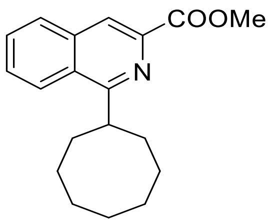

Methyl 1-cyclooctylisoquinoline-3-carboxylate (5c). 5c was synthesized following the general procedure. The residue was purified by chromatography on silica gel, eluting with hexane/ethyl acetate (10:1) to give the title compound as colorless oil (38.7mg, 65%).

5c: ^{1}H NMR (400 MHz, $CDCl_3$) δ 8.40 (s, 1H), 8.30 – 8.24 (m, 1H), 7.98 – 7.92 (m, 1H), 7.75 – 7.39 (m, 2H), 4.04 (s, 3H), 3.87 (tt, *J* = 9.5, 3.4 Hz, 1H), 2.23 – 2.12 (m, 2H), 2.08 – 1.90 (m, 2H), 1.96 – 1.87 (m, 2H). 1.83 – 1.64 (m, 8H). ^{13}C NMR (101 MHz, $CDCl_3$) δ 168.3, 166.9, 140.6, 136.3, 130.2, 129.2, 129.1, 127.5, 125.3, 122.4, 52.7, 41.9, 33.0, 27.0, 26.8, 26.4.

These spectroscopic data correspond to reported data.[2]

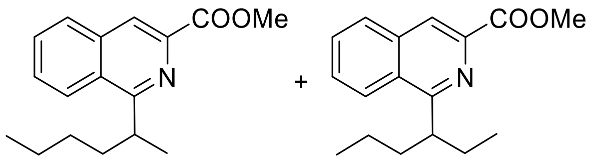

Methyl 1-(hexan-2-yl)isoquinoline-3-carboxylate (5d). 5d and **5d'** was synthesized following the general procedure. The residue was purified by chromatography on silica gel, eluting with hexane/ethyl acetate (10:1) to give the title compound as colorless oil (16.2 mg, 30%).

5d: ^{1}H NMR (400 MHz, $CDCl_3$) δ 8.39 (s, 1H), 8.31–8.25 (m, 1H), 7.96–7.92 (m, 1H), 7.74–7.67 (m, 2H), 4.02 (s, 3H), 3.83–3.73 (m, 1H), 2.10– 1.97 (m, 2H), 1.82–1.72 (m, 1H), 1.46 (d, J = 6.5 Hz, 3H), 1.41–1.20 (m, 3H), 0.86 (t, J = 7.2 Hz, 3H); ^{13}C NMR (101 MHz, $CDCl_3$) δ 167.0, 166.7, 140.8, 136.2, 130.2, 129.2, 128.3, 125.0, 122.4, 52.7, 37.00, 36.3, 30.3, 23.0, 20.2, 14.2.

Methyl 1-(hexan-3-yl)isoquinoline-3-carboxyla (5d').

5d': ^{1}H NMR (400 MHz, $CDCl_3$) δ 8.38 (s, 1H), 8.31–8.25 (m, 1H), 7.96– 7.92 (m, 1H), 7.74–7.67 (m, 2H), 4.02 (s, 3H), 3.66–3.57 (m, 1H), 1.90–1.82 (m, 1H), 1.83–1.72 (m, 1H), 1.41–1.20 (m, 3H), 1.18–1.08 (m, 1H), 0.85 (t, J = 7.4 Hz, 3H), 0.79 (t, J = 7.4 Hz, 3H); ^{13}C NMR (101 MHz, $CDCl_3$) δ 167.0, 166.0, 141.0, 136.0, 130.2, 129.4, 129.1, 125.1, 122.1, 52.7, 37.5, 37.0, 28.4, 21.2, 14.4, 12.6.

These spectroscopic data correspond to reported data.[2]

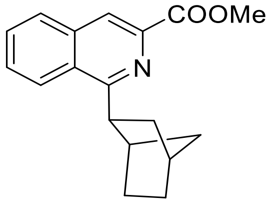

4-(4-methoxybenzyl)-N,N-dimethylbenzamide (5e). 5e was synthesized following the general procedure. The residue was purified by chromatography on silica gel, eluting with hexane/ethyl acetate (10:1) to give the title compound as colorless oil (35.2 mg, 63%).

5e: ^{1}H NMR (400 MHz, $CDCl_3$) δ 8.39 (s, 1H), 8.29 – 8.23 (m, 1H), 7.97 – 7.90 (m, 1H), 7.75 – 7.69 (m, 2H),, 4.04 (s, 3H), 3.63 – 3.57 (m, 1H), 2.69 – 2.61 (m, 1H), 2.58 (s, 1H), 2.47 (s, 1H), 1.76 – 1.66 (m, 4H), 1.65 – 1.57 (m, 1H), 1.47 – 1.39 (m, 1H), 1.17 (d, J = 9.5 Hz, 1H). ^{13}C NMR (101 MHz, $CDCl_3$) δ 167.0, 164.5, 140.1, 136.0,

130.1, 129.0, 129.0, 128.5, 125.5, 122.3, 52.6, 45.7, 43.5, 36.8, 36.0, 35.3, 30.4, 29.5.

These spectroscopic data correspond to reported data.[2]

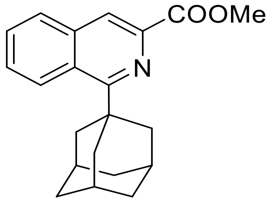

Methyl 1-((3*r*,5*r*,7*r*)-adamantan-1-yl)isoquinoline-3-carboxylate (5f). 5f was synthesized following the general procedure. The residue was purified by chromatography on silica gel, eluting with hexane/ethyl acetate (10:1) to give the title compound as white solid (36.3 mg, 57%).

5f: ^{1}H NMR (400 MHz, $CDCl_3$) δ 8.86 – 8.78 (m, 1H), 8.41 (s, 1H), 8.02 – 7.93 (m, 1H), 7.73 – 7.64 (m, 2H), 4.05 (s, 3H), 2.46 (d, J = 2.3 Hz, 6H), 2.27 – 2.20 (m, 3H), 1.91 (q, J = 12.3 Hz, 6H). 13C NMR (101 MHz, CDCl3) δ 167.3, 167.0, 139.6, 137.4, 130.1, 129.4, 127.9, 127.8, 127.3, 123.2, 52.7, 43.1, 42.0, 37.2, 29.3.

These spectroscopic data correspond to reported data.[3]

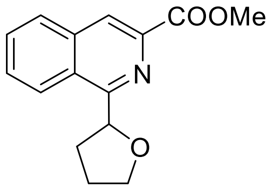

Methyl 1-(tetrahydrofuran-2-yl)isoquinoline-3-carboxylate (5g). 5g was synthesized following the general procedure. The residue was purified by chromatography on silica gel, eluting with hexane/ethyl acetate (3:1) to give the title compound as colorless oil (31.2 mg, 61%).

5g: ^{1}H NMR (400 MHz, $CDCl_3$) δ 8.51 – 8.45 (m, 2H), 7.98 – 7.92 (m, 1H), 7.76 – 7.67 (m, 2H), 5.66 (t, J = 7.2 Hz, 1H), 4.20 – 4.11 (m, 1H), 4.06 – 3.99 (m, 1H), 4.02 (s, 3H), 2.74 – 2.63 (m, 1H), 2.46 – 2.35 (m, 1H), 2.26 – 2.05 (m, 2H). ^{13}C NMR (101 MHz, $CDCl_3$) δ 166.6, 160.1, 140.1, 136.5, 130.6, 129.4, 128.9, 128.3, 126.1, 124.2, 80.5, 69.1, 52.8, 30.4, 26.2.

These spectroscopic data correspond to reported data.[4]

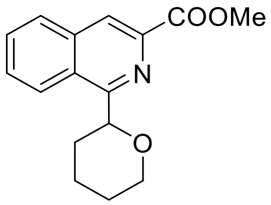

Methyl 1-(tetrahydro-2*H*-pyran-2-yl)isoquinoline-3-carboxylate (5h). 5h was synthesized following the general procedure. The residue was purified by chromatography on silica gel, eluting with hexane/ethyl acetate (3:1) to give the title compound as white solid (35.7 mg, 66%).

5h: ^{1}H NMR (400 MHz, $CDCl_3$) δ 8.70 (d, *J* = 7.4 Hz, 1H), 8.51 (s, 1H), 7.97 (dd, *J* = 6.5, 2.4 Hz, 1H), 7.79 – 7.70 (m, 2H), 5.18 (dd, *J* = 11.4, 2.0 Hz, 1H), 4.26 (d, *J* = 11.1 Hz, 1H), 4.05 (s, 3H), 3.78 (td, *J* = 11.6, 2.2 Hz, 1H), 2.29 – 2.18 (m, 1H), 2.20 – 2.12 (m, 2H), 1.94 – 1.79 (m, 2H), 1.74 – 1.66 (m, 1H),. ^{13}C NMR (101 MHz, $CDCl_3$) δ 166.6, 160.7, 140.1, 136.8, 130.6, 129.3, 128.9, 127.9, 126.7, 124.4, 82.1, 69.4, 53.0, 30.8, 26.0, 23.8.

HRMS (ESI/QTOF) m/z: $[M + H]^+$ Calcd for $C_{16}H_{18}NO_3^+$ 272.1281; Found 272.1283.

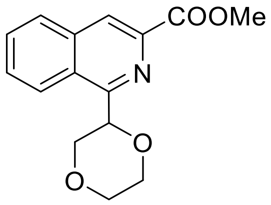

Methyl 1-(1,4-dioxan-2-yl)isoquinoline-3-carboxylate (5i). 5i was synthesized following the general procedure. The residue was purified by chromatography on silica gel, eluting with hexane/ethyl acetate (10:1) to give the title compound as colorless oil (38.0 mg, 70%).

5i: ^{1}H NMR (400 MHz, $CDCl_3$) δ 8.55 – 8.47 (m, 2H), 7.97 – 7.90 (m, 1H), 7.77 – 7.70 (m, 2H), 5.40 (dd, *J* = 9.7, 3.0 Hz, 1H), 4.32 – 4.18 (m, 2H), 4.10 – 3.98 (m, 2H), 4.00 (s, 3H), 3.92 – 3.84 (m, 2H). ^{13}C NMR (101 MHz, $CDCl_3$) δ 166.3, 156.8, 140.3, 136.5, 130.8, 129.6, 128.9, 128.3, 125.9, 124.6, 77.3, 69.7, 67.6, 66.5, 52.8.

HRMS (ESI/QTOF) m/z: $[M + Na]^+$ Calcd for $C_{15}H_{15}NNaO_4^+$ 296.0893; Found 296.0891.

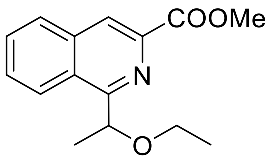

Methyl 1-(1-ethoxyethyl)isoquinoline-3-carboxylate (5j). 5j was synthesized following the general procedure. The residue was purified by chromatography on silica gel, eluting with hexane/ethyl acetate (5:1) to give the title compound as colorless oil (46.3 mg, 89%).

5j: ^{1}H NMR (400 MHz, $CDCl_3$) δ 8.90 (d, *J* = 8.4 Hz, 1H), 8.46 (s, 1H), 7.93 (d, *J* = 8.2 Hz, 1H), 7.74 – 7.63 (m, 2H), 5.18 (q, *J* = 6.8 Hz, 1H), 4.00 (s, 3H), 3.53 (dq, *J* = 8.9, 6.8 Hz, 1H), 3.31 (dq, *J* = 9.0, 7.0 Hz, 1H), 1.69 (d, *J* = 6.8 Hz, 3H), 1.14 (t, *J* = 7.0 Hz, 3H). ^{13}C NMR (101 MHz, $CDCl_3$) δ 166.5, 162.9, 140.1, 136.7, 130.7, 129.1, 128.9, 127.5, 126.4, 124.3, 82.0, 64.7, 52.9, 21.9, 15.5.

HRMS (ESI/QTOF) m/z: $[M + H]^+$ Calcd for $C_{15}H_{18}NO_3^+$ 260.1281; Found 260.1281.

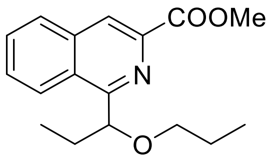

Methyl 1-(1-propoxypropyl)isoquinoline-3-carboxylate (5k). 5k was synthesized following the general procedure. The residue was purified by chromatography on silica gel, eluting with hexane/ethyl acetate (50:1) to give the title compound as colorless oil (53.4 mg, 93%).

5k: ^{1}H NMR (400 MHz, $CDCl_3$) δ 8.92 (d, *J* = 8.3 Hz, 1H), 8.46 (s, 1H), 7.93 (d, *J* = 8.4 Hz, 1H), 7.71 (t, *J* = 7.5 Hz, 1H), 7.65 (t, *J* = 7.7 Hz, 1H), 4.89 (dd, *J* = 8.9, 5.4 Hz, 1H), 4.01 (s, 3H), 3.39 (dt, *J* = 9.0, 6.5 Hz, 1H), 3.24 (dt, *J* = 9.1, 6.6 Hz, 1H), 2.22 – 2.10 (m, 1H), 1.98 – 1.86 (m, 1H), 1.59 – 1.48 (m, 2H), 1.00 (t, *J* = 7.4 Hz, 3H), 0.82 (t, *J* = 7.4 Hz, 3H). ^{13}C NMR (101 MHz, $CDCl_3$) δ 166.6, 162.7, 140.1, 136.6, 130.6, 128.9, 128.8, 127.8, 126.8, 124.2, 88.0, 71.4, 52.9, 29.5, 23.2, 11.0, 10.7.

HRMS (ESI/QTOF) m/z: $[M + H]^+$ Calcd for $C_{17}H_{22}NO_3^+$ 288.1594; Found 288.1590.

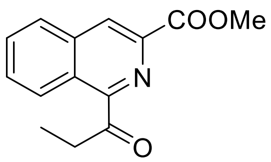

Methyl 1-propionylisoquinoline-3-carboxylate (5l). 5l was synthesized following the general procedure. The residue was purified by chromatography on silica gel, eluting with hexane/ethyl acetate (5:1) to give the title compound as white solid (35.3 mg, 73%).

5l: ^{1}H NMR (400 MHz, $CDCl_3$) δ 8.86 – 8.80 (m, 1H), 8.65 (s, 1H), 8.01 – 7.95 (m, 1H), 7.81 – 7.74 (m, 2H), 4.05 (s, 3H), 3.41 (q, J = 7.2 Hz, 2H), 1.26 (t, J = 7.2 Hz, 3H). ^{13}C NMR (101 MHz, $CDCl_3$) δ 204.8, 165.9, 153.9, 139.8, 137.0, 131.2, 131.2, 128.5, 127.2, 127.0, 126.8, 53.0, 33.9, 7.9.

HRMS (ESI/QTOF) m/z: $[M + H]^+$ Calcd for $C_{14}H_{14}NO_3^+$ 244.0968; Found 244.0965.

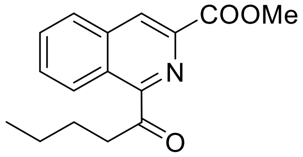

Methyl 1-pentanoylisoquinoline-3-carboxylate (5m). 5m was synthesized following the general procedure. The residue was purified by chromatography on silica gel, eluting with hexane/CH_2ClCH_2Cl (5:1) to give the title compound as white solid (40.5 mg, 75%).

5m: ^{1}H NMR (400 MHz, $CDCl_3$) δ 8.84 – 8.77 (m, 1H), 8.65 (s, 1H), 8.00 – 7.94 (m, 1H), 7.79 – 7.73 (m, 2H), 4.04 (s, 3H), 3.37 (t, J = 7.4 Hz, 2H), 1.75 (p, J = 7.5 Hz, 2H), 1.50 – 1.39 (m, 2H), 0.95 (t, J = 7.3 Hz, 3H). ^{13}C NMR (101 MHz, $CDCl_3$) δ 204.5, 165.8, 154.0, 139.8, 136.9, 131.1, 131.1, 128.5, 127.1, 127.0, 126.8, 52.9, 40.3, 26.2, 22.5, 14.1.

HRMS (ESI/QTOF) m/z: $[M + Na]^+$ Calcd for $C_{16}H_{17}NNaO_3^+$ 294.1101; Found 294.1103.

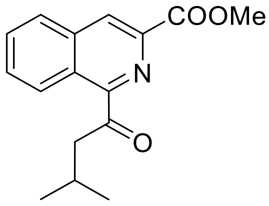

Methyl 1-(3-methylbutanoyl)isoquinoline-3-carboxylate (5n). 5n was synthesized following the general procedure. The residue was purified by chromatography on silica gel, eluting with hexane/ethyl acetate (10:1) to give the title compound as white solid (42.2 mg, 78%).

5n: ^{1}H NMR (400 MHz, $CDCl_3$) δ 8.84 (dd, *J* = 6.3, 3.4 Hz, 1H), 8.67 (s, 1H), 8.00 (dd, *J* = 6.3, 3.1 Hz, 1H), 7.79 (dt, *J* = 6.4, 3.4 Hz, 2H), 4.07 (s, 3H), 3.28 (d, *J* = 6.9 Hz, 2H), 2.40 – 2.28 (m, 1H), 1.05 (d, *J* = 6.7 Hz, 6H). ^{13}C NMR (101 MHz, $CDCl_3$) δ 204.2, 165.9, 154.1, 139.8, 137.0, 131.2, 131.2, 128.6, 127.1, 127.0, 126.8, 52.9, 49.2, 25.1, 22.9.

HRMS (ESI/QTOF) m/z: $[M + Na]^+$ Calcd for $C_{16}H_{17}NNaO_3^+$ 294.1101; Found 294.1098.

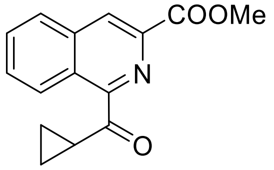

Methyl 1-(cyclopropanecarbonyl)isoquinoline-3-carboxylate (5o). 5o was synthesized following the general procedure. The residue was purified by chromatography on silica gel, eluting with hexane/ethyl acetate (5:1) to give the title compound as white solid (32.8 mg, 64%).

5o: ^{1}H NMR (400 MHz, $CDCl_3$) δ 8.92 – 8.86 (m, 1H), 8.68 (s, 1H), 8.02 – 7.95 (m, 1H), 7.80 – 7.73 (m, 2H), 4.06 (s, 3H), 3.48 (tt, *J* = 8.2, 4.7 Hz, 1H), 1.38 – 1.33 (m, 2H), 1.20 – 1.14 (m, 2H). ^{13}C NMR (101 MHz, $CDCl_3$) δ 203.1, 165.9, 153.8, 139.9, 137.0, 131.2, 131.2, 128.5, 127.3, 127.2, 126.9, 53.0, 19.3, 13.5.

HRMS (ESI/QTOF) m/z: $[M + H]^+$ Calcd for $C_{15}H_{14}NO_3^+$ 256.0968; Found 256.0964.

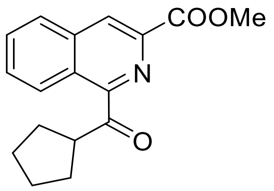

Methyl 1-(cyclopentanecarbonyl)isoquinoline-3-carboxylate (5p). 5p and **5p'** were synthesized following the general procedure. The residue was purified by chromatography on silica gel, eluting with hexane/CH_2ClCH_2Cl (2:1) to give the title compound as colorless oil (**5p:** 27.3 mg, 48%; **5p':** 11.0 mg, 22%;).

5p: ^{1}H NMR (400 MHz, $CDCl_3$) δ 8.72 – 8.67 (m, 1H), 8.65 (s, 1H), 8.02 – 7.97 (m, 1H), 7.81 – 7.74 (m, 2H), 4.39 (p, *J* = 8.1, 7.6 Hz, 1H), 4.05 (s, 3H), 1.99 – 1.83 (m, 4H), 1.77 – 1.64 (m, 4H). ^{13}C NMR (101 MHz, $CDCl_3$) δ 205.9, 166.0, 155.1, 140.0,

136.9, 131.2, 131.0, 128.5, 127.3, 126.9, 126.8, 53.0, 48.3, 29.1, 26.4.

HRMS (ESI/QTOF) m/z: $[M + H]^+$ Calcd for $C_{17}H_{18}NO_3^+$ 284.1281; Found 284.1284.

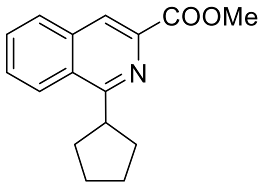

Methyl 1-cyclopentylisoquinoline-3-carboxylate (5p'): ^{1}H NMR (400 MHz, $CDCl_3$) δ 8.42 (s, 1H), 8.34 – 8.28 (m, 1H), 7.98 – 7.94 (m, 1H), 7.78 – 7.70 (m, 2H), 4.06 – 3.96 (m, 1H), 4.04 (s, 3H), 2.26 – 2.16 (m, 4H), 2.02 – 2.91 (m, 2H), 1.86 – 1.76 (m, 2H). ^{13}C NMR (101 MHz, $CDCl_3$) δ 167.0, 165.2, 140.5, 136.1, 130.3, 129.1, 129.0, 128.7, 125.6, 122.7, 52.8, 43.9, 32.6, 26.0.

These spectroscopic data correspond to reported data.[2]

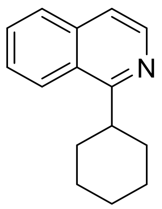

1-cyclohexylisoquinoline (6a). 6a was synthesized following the general procedure. The residue was purified by chromatography on silica gel, eluting with hexane/ethyl acetate (10:1) to give the title compound as colorless oil (32.5 mg, 77%).

6a: ^{1}H NMR (400 MHz, $CDCl_3$) δ 8.50 (d, J = 5.7 Hz, 1H), 8.24 (d, J = 8.4 Hz, 1H), 7.82 (d, J = 8.2 Hz, 1H), 7.59 (td, J = 7.5, 1.1 Hz, 1H), 7.59 (ddd, J = 8.3, 6.9, 1.4 Hz, 1H), 7.49 (d, J = 5.7 Hz, 1H), 3.58 (tt, J = 11.7, 3.3 Hz, 1H), 2.05 – 1.93 (m, 4H), 1.91 – 1.81 (m, 3H), 1.62 – 1.50 (m, 2H), 1.43 (tt, J = 12.7, 3.3 Hz, 1H). ^{13}C NMR (101 MHz, $CDCl_3$) δ 165.8, 142.0, 136.5, 129.6, 127.6, 126.9, 126.4, 124.8, 119.0, 41.6, 32.7, 27.0, 26.4.

These spectroscopic data correspond to reported data.[5]

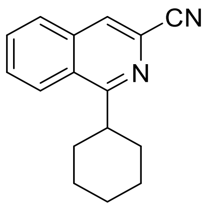

1-cyclohexylisoquinoline-3-carbonitrile (6b). 6b was synthesized following the general procedure. The residue was purified by chromatography on silica gel, eluting with hexane/ethyl acetate (5:1) to give the title compound as white solid (29.0 mg, 61%).

6b: ^{1}H NMR (400 MHz, $CDCl_3$) δ 8.33 – 8.26 (m, 1H), 7.98 (s, 1H), 7.93 – 7.87 (m, 1H), 7.83 – 7.75 (m, 2H), 3.56 (tt, *J* = 11.6, 3.3 Hz, 1H), 2.00 – 1.91 (m, 4H), 1.88 – 1.78 (m, 3H), 1.60 – 1.47 (m, 2H), 1.46 – 1.37 (m, 1H). ^{13}C NMR (101 MHz, $CDCl_3$) δ 168.0, 135.3, 131.1, 130.0, 128.2, 127.4, 126.2, 126.1, 125.2, 118.8, 41.9, 32.4, 26.8, 26.2.

These spectroscopic data correspond to reported data.[5]

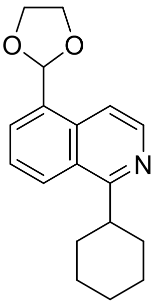

1-cyclohexyl-5-(1,3-dioxolan-2-yl)isoquinoline (6c). 6c was synthesized following the general procedure. The residue was purified by chromatography on silica gel, eluting with hexane/ethyl acetate (5:1) to give the title compound as colorless oil (26.4 mg, 47%).

6c: ^{1}H NMR (400 MHz, $CDCl_3$) δ 8.52 (d, *J* = 6.0 Hz, 1H), 8.26 (d, *J* = 8.6 Hz, 1H), 7.92 (d, *J* = 7.1 Hz, 1H), 7.83 (d, *J* = 6.0 Hz, 1H), 7.57 (t, *J* = 7.9 Hz, 1H), 6.39 (s, 1H), 4.22 – 4.11 (m, 4H), 3.57 (tt, *J* = 11.6, 3.2 Hz, 1H), 2.00 – 1.89 (m, 4H), 1.88 – 1.76 (m, 3H), 1.59 – 1.46 (m, 2H), 1.40 (tt, *J* = 12.6, 3.3 Hz, 1H). ^{13}C NMR (101 MHz, $CDCl_3$) δ 166.3, 142.4, 134.4, 133.3, 127.1, 126.6, 126.1, 126.1, 115.2, 101.9, 65.5, 41.9, 32.8, 27.0, 26.4.

HRMS (ESI/QTOF) m/z: $[M + H]^+$ Calcd for $C_{18}H_{22}NO_2^+$ 284.1645; Found 284.1649.

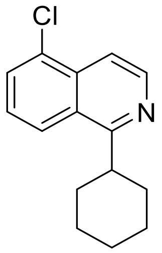

5-chloro-1-cyclohexylisoquinoline (6d). 6d was synthesized following the general procedure. The residue was purified by chromatography on silica gel, eluting with hexane/ethyl acetate (10:1) to give the title compound as colorless oil (34.2 mg, 70%).

6d: ^{1}H NMR (400 MHz, $CDCl_3$) δ 8.60 (d, *J* = 5.9 Hz, 1H), 8.17 (d, *J* = 8.5 Hz, 1H), 7.90 (dd, *J* = 5.9, 1.0 Hz, 1H), 7.74 (dd, *J* = 7.5, 1.0 Hz, 1H), 7.50 (dd, *J* = 8.6, 7.5 Hz, 1H), 3.56 (tt, *J* = 11.6, 3.3 Hz, 1H), 2.03 – 1.92 (m, 4H), 1.90 – 1.78 (m, 3H), 1.61 – 1.48 (m, 2H), 1.42 (tt, *J* = 12.7, 3.3 Hz, 1H).^{13}C NMR (101 MHz, $CDCl_3$) δ 166.1, 143.2, 134.4, 132.0, 129.7, 127.4, 126.6, 123.9, 115.1, 42.0, 32.8, 26.9, 26.3.

These spectroscopic data correspond to reported data.[6]

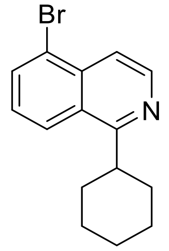

4-bromo-1-cyclohexylisoquinoline (6e). 6e was synthesized following the general procedure. The residue was purified by chromatography on silica gel, eluting with hexane/ethyl acetate (10:1) to give the title compound as colorless oil (46.2 mg, 80%).

6e: ^{1}H NMR (400 MHz, $CDCl_3$) δ 8.59 (d, *J* = 5.9 Hz, 1H), 8.21 (d, *J* = 8.5 Hz, 1H), 7.93 (d, *J* = 7.4 Hz, 1H), 7.87 (d, *J* = 5.9 Hz, 1H), 7.87 (t, *J* = 8.0 Hz, 1H), 3.55 (tt, *J* = 11.6, 3.3 Hz, 1H), 2.02 – 1.91 (m, 4H), 1.90 – 1.78 (m, 3H), 1.60 – 1.47 (m, 2H), 1.41 (tt, *J* = 12.7, 3.3 Hz, 1H).^{13}C NMR (101 MHz, $CDCl_3$) δ 166.2, 143.4, 135.6, 133.5, 127.5, 127.1, 124.6, 122.7, 117.8, 41.9, 32.8, 26.9, 26.3.

These spectroscopic data correspond to reported data.[6]

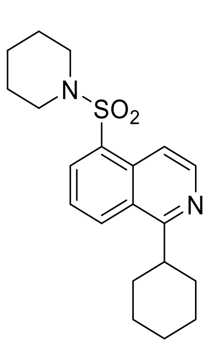

1-cyclohexyl-5-(piperidin-1-ylsulfonyl)isoquinoline (6f). 6f was synthesized

following the general procedure. The residue was purified by chromatography on silica gel, eluting with hexane/ethyl acetate (5:1) to give the title compound as white solid (56.7 mg, 79%).

6f: ^{1}H NMR (400 MHz, $CDCl_3$) δ 8.58 (d, *J* = 6.1 Hz, 1H), 8.47 (d, *J* = 8.6 Hz, 1H), 8.36 – 8.31 (m, 2H), 7.64 (t, *J* = 8.0 Hz, 1H), 3.54 (tt, *J* = 11.5, 3.2 Hz, 1H), 3.15 (t, *J* = 5.5 Hz, 4H), 1.98 – 1.88 (m, 4H), 1.87 – 1.75 (m, 3H), 1.60 – 1.51 (m, 5H), 1.50 – 1.36 (m, 4H). ^{13}C NMR (101 MHz, $CDCl_3$) δ 166.6, 143.7, 133.6, 133.5, 132.7, 130.5, 126.9, 125.2, 115.8, 46.4, 42.1, 32.8, 26.8, 26.2, 25.4, 23.6.

HRMS (ESI/QTOF) m/z: $[M + H]^+$ Calcd for $C_{20}H_{27}N_2O_2S^+$ 359.1788; Found 359.1780.

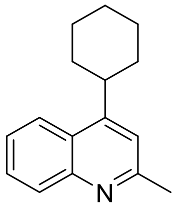

4-cyclohexyl-2-methylquinoline (6g). 6g was synthesized following the general procedure. The residue was purified by chromatography on silica gel, eluting with hexane/ethyl acetate (5:1) to give the title compound as colorless oil (40.8 mg, 91%).

6g: ^{1}H NMR (400 MHz, $CDCl_3$) δ 8.03 (dd, *J* = 8.3, 4.6 Hz, 2H), 7.64 (t, *J* = 7.2 Hz, 1H), 7.47 (t, *J* = 7.6 Hz, 1H), 7.16 (s, 1H), 3.33 – 3.23 (m, 1H), 2.71 (s, 3H), 2.05 – 1.80 (m, 5H), 1.60 – 1.46 (m, 4H), 1.39 – 1.29 (m, 1H),. ^{13}C NMR (101 MHz, $CDCl_3$) δ 158.9, 153.5, 148.2, 129.6, 128.9, 125.4, 125.3, 122.9, 118.4, 38.9, 33.7, 27.1, 26.4, 25.6.

These spectroscopic data correspond to reported data.[5]

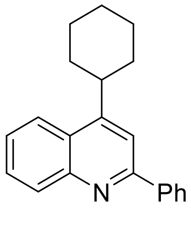

4-cyclohexyl-2-phenylquinoline (6h). 6h was synthesized following the general procedure. The residue was purified by chromatography on silica gel, eluting with hexane/ethyl acetate (10:1) to give the title compound as white solid (52.0 mg, 91%).

6h: ^{1}H NMR (400 MHz, $CDCl_3$) δ 8.22 (d, *J* = 8.4 Hz, 1H), 8.18 – 8.14 (m, 2H), 8.10 (d, *J* = 8.3 Hz, 1H), 7.78 (s, 1H), 7.72 (ddd, *J* = 8.2, 6.8, 1.1 Hz, 1H), 7.57 – 7.51 (m, 3H), 7.49 – 7.44 (m, 1H), 3.43 – 3.34 (m, 1H), 2.14 – 2.06 (m, 2H), 2.00 – 1.94 (m, 2H), 1.92 – 1.85 (m, 1H), 1.70 – 1.53 (m, 4H), 1.45 – 1.35 (m, 1H). ^{13}C NMR (101 MHz, $CDCl_3$) δ 157.5, 154.2, 148.7, 140.3, 130.8, 129.3, 129.2, 128.9, 127.8, 126.0, 126.0, 123.0, 115.6, 39.3, 33.8, 27.1, 26.5.

These spectroscopic data correspond to reported data.[5]

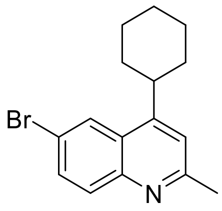

6-bromo-4-cyclohexyl-2-methylquinoline (6i). 6i was synthesized following the general procedure. The residue was purified by chromatography on silica gel, eluting with hexane/ethyl acetate (10:1) to give the title compound as colorless oil (41.2 mg, 68%).

6i: ^{1}H NMR (400 MHz, $CDCl_3$) δ 8.16 (d, *J* = 2.1 Hz, 1H), 7.92 (d, *J* = 8.9 Hz, 1H), 7.73 (dd, *J* = 8.9, 2.2 Hz, 1H), 7.19 (s, 1H), 3.25 – 3.15 (m, 1H), 2.72 (s, 3H), 2.02 – 1.92 (m, 4H), 1.91 – 1.84 (m, 1H), 1.64 – 1.47 (m, 4H), 1.40 – 1.32 (m, 1H). ^{13}C NMR (101 MHz, $CDCl_3$) δ 159.4, 152.7, 146.8, 132.3, 131.3, 126.6, 125.4, 119.5, 119.3, 38.8, 33.6, 26.9, 26.3, 25.5.

These spectroscopic data correspond to reported data.[7]

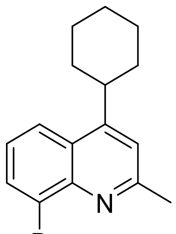

8-bromo-4-cyclohexyl-2-methylquinoline (6j). 6j was synthesized following the general procedure. The residue was purified by chromatography on silica gel, eluting with hexane/ethyl acetate (10:1) to give the title compound as colorless oil (37.9 mg, 63%).

6j: ^{1}H NMR (400 MHz, $CDCl_3$) δ 8.02 (td, *J* = 8.1, 7.5, 1.3 Hz, 2H), 7.34 (t, *J* = 7.9 Hz, 1H), 7.23 (s, 1H), 3.33 – 3.24 (m, 1H), 2.81 (s, 1H), 2.04 – 1.92 (m, 4H), 1.91 – 1.84 (m, 1H), 1.62 – 1.48 (m, 4H), 1.37 (tt, *J* = 12.5, 3.7 Hz, 1H). ^{13}C NMR (101 MHz, $CDCl_3$) δ 160.2, 153.8, 145.3, 132.6, 126.7, 125.5, 125.3, 122.9, 119.2, 39.1, 33.7, 27.0, 26.3, 26.0.

HRMS (ESI/QTOF) m/z: $[M + H]^+$ Calcd for $C_{16}H_{19}BrN^+$ 304.0695; Found 304.0697.

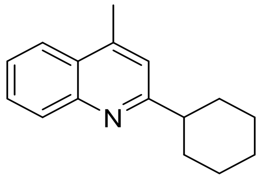

2-cyclohexyl-4-methylquinoline (6k). 6k was synthesized following the general procedure. The residue was purified by chromatography on silica gel, eluting with hexane/ethyl acetate (5:1) to give the title compound as colorless oil (28.3 mg, 63%).

6k: ^{1}H NMR (400 MHz, $CDCl_3$) δ 8.06 (d, *J* = 8.4 Hz, 1H), 7.93 (d, *J* = 8.3 Hz, 1H), 7.66 (t, *J* = 7.2 Hz, 1H), 7.49 (t, *J* = 7.6 Hz, 1H), 7.17 (s, 1H), 2.88 (tt, *J* = 12.0, 3.4 Hz, 2H), 2.68 (s, 3H), 2.05 – 1.97 (m, 2H), 1.93 – 1.85 (m, 2H), 1.82 – 1.75 (m, 1H), 1.68 – 1.57 (m, 1H), 1.53 – 1.41 (m, 2H), 1.35 (tt, *J* = 12.9, 3.3 Hz, 1H). ^{13}C NMR (101 MHz, $CDCl_3$) δ 166.6, 147.7, 144.4, 129.5, 129.1, 127.1, 125.5, 123.7, 120.3, 47.6, 32.9, 26.7, 26.2, 18.9.

These spectroscopic data correspond to reported data.[5]

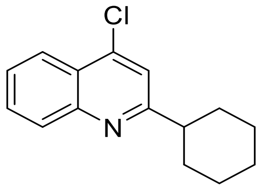

4-chloro-2-cyclohexylquinoline (6l). 6l was synthesized following the general procedure. The residue was purified by chromatography on silica gel, eluting with hexane/ethyl acetate (10:1) to give the title compound as colorless oil (39.8 mg, 81%).

6l: ^{1}H NMR (400 MHz, $CDCl_3$) δ 8.16 (d, *J* = 7.7 Hz, 1H), 8.05 (d, *J* = 8.4 Hz, 1H), 7.71 (t, *J* = 7.7 Hz, 1H), 7.55 (t, *J* = 7.6 Hz, 1H), 7.41 (s, 1H), 2.88 (tt, *J* = 12.0, 3.4 Hz, 1H), 2.05 – 1.97 (m, 2H), 1.93 – 1.85 (m, 2H), 1.82 – 1.74 (m, 1H), 1.66 – 1.54 (m, 2H), 1.51 – 1.39 (m, 2H), 1.33 (tt, *J* = 12.6, 3.3 Hz, 1H).^{13}C NMR (101 MHz, $CDCl_3$)

δ 166.9, 148.8, 142.7, 130.2, 129.4, 126.7, 125.2, 124.0, 119.9, 47.5, 32.8, 26.5, 26.1.

These spectroscopic data correspond to reported data.[5]

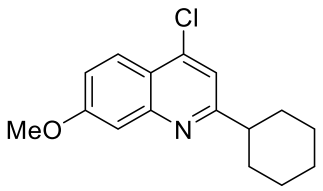

4-chloro-2-cyclohexyl-7-methoxyquinoline (6m). 6m was synthesized following the general procedure. The residue was purified by chromatography on silica gel, eluting with hexane/ethyl acetate (10:1) to give the title compound as pale yellow oil (52.9 mg, 96%).

6m: ^{1}H NMR (400 MHz, $CDCl_3$) δ 8.02 (d, *J* = 9.2 Hz, 1H), 7.37 (d, *J* = 2.5 Hz, 1H), 7.25 (s, 1H), 7.18 (dd, *J* = 9.1, 2.4 Hz, 1H), 3.93 (s, 3H), 2.83 (tt, *J* = 12.0, 3.4 Hz, 1H), 2.04 – 1.96 (m, 2H), 1.91 – 1.84 (m, 2H), 1.80 – 1.73 (m, 1H), 1.63 – 1.52 (m, 2H), 1.38 – 1.50 (m, 2H). 1.32 (tt, *J* = 12.4, 3.2 Hz, 1H). ^{13}C NMR (101 MHz, $CDCl_3$) δ 167.4, 161.4, 150.6, 142.6, 125.1, 120.2, 119.8, 117.7, 107.4, 55.7, 47.5, 32.9, 26.6, 26.1.

These spectroscopic data correspond to reported data.[8]

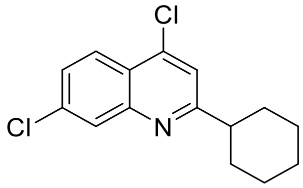

4,6-dichloro-2-cyclohexylquinoline (6n). 6n was synthesized following the general procedure. The residue was purified by chromatography on silica gel, eluting with hexane/ethyl acetate (10:1) to give the title compound as colorless oil (44.5 mg, 80%).

6n: ^{1}H NMR (400 MHz, $CDCl_3$) δ 8.06 (d, *J* = 8.9 Hz, 1H), 8.04 (d, *J* = 2.1 Hz, 1H), 7.49 (dd, *J* = 8.9, 2.1 Hz, 1H), 7.38 (s, 1H), 2.84 (tt, *J* = 11.9, 3.4 Hz, 1H), 2.04 – 1.96 (m, 2H), 1.93 – 1.85 (m, 2H), 1.81 – 1.74 (m, 1H), 1.65 – 1.54 (m, 2H), 1.50 – 1.38 (m, 2H), 1.33 (tt, *J* = 12.5, 3.3 Hz, 1H). ^{13}C NMR (101 MHz, $CDCl_3$) δ 168.2, 149.2, 142.6, 136.3, 128.5, 127.6, 125.4, 123.7, 120.2, 47.4, 32.7, 26.5, 26.1.

These spectroscopic data correspond to reported data.[6]

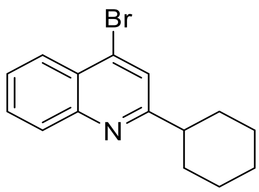

4-bromo-2-cyclohexylquinoline (6o). 6o was synthesized following the general procedure. The residue was purified by chromatography on silica gel, eluting with hexane/ethyl acetate (10:1) to give the title compound as pale yellow oil (41.7 mg, 72%).

6o: ^{1}H NMR (400 MHz, $CDCl_3$) δ 8.14 (d, *J* = 8.3 Hz, 1H), 8.05 (d, *J* = 8.6 Hz, 1H), 7.73 (t, *J* = 7.2 Hz, 1H), 7.64 (s, 1H), 7.58 (t, *J* = 7.6 Hz, 1H), 2.90 (tt, *J* = 12.0, 3.5 Hz, 1H), 2.08 – 2.00 (m, 2H), 1.96 – 1.87 (m, 2H), 1.85 – 1.76 (m, 1H), 1. 75 – 1.70 (m, 2H), 1.42 – 1.54 (m, 2H) 1.36 (tt, *J* = 12.5, 3.3 Hz, 1H). ^{13}C NMR (101 MHz, $CDCl_3$) δ 166.9, 148.6, 134.3, 130.3, 129.5, 127.0, 126.7, 126.6, 123.8, 47.4, 32.8, 26.6, 26.1.

These spectroscopic data correspond to reported data.[9]

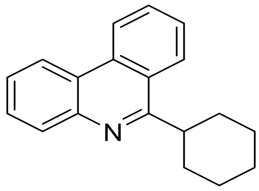

6-cyclohexylphenanthridine (6p). 6p was synthesized following the general procedure. The residue was purified by chromatography on silica gel, eluting with hexane/ethyl acetate (10:1) to give the title compound as white solid (43.4 mg, 83%).

6p: ^{1}H NMR (400 MHz, $CDCl_3$) δ 8.67 (d, *J* = 8.2 Hz, 1H), 8.56 (d, *J* = 8.2 Hz, 1H), 8.35 (d, *J* = 8.3 Hz, 1H), 8.19 (d, *J* = 8.0 Hz, 1H), 7.83 (ddd, *J* = 8.2, 6.9, 1.1 Hz, 1H), 7.77 – 7.68 (m, 2H), 7.63 (ddd, *J* = 8.2, 7.1, 1.3 Hz, 1H), 3.66 (tt, *J* = 11.2, 3.4 Hz, 1H), 2.17 – 2.09 (m, 2H), 2.06 – 1.94 (m, 4H), 1.92 – 1.86 (m, 1H), 1.68 – 1.56 (m, 2H), 1.50 (tt, *J* = 12.9, 3.2 Hz, 1H). ^{13}C NMR (101 MHz, $CDCl_3$) δ 165.4, 144.0, 133.1, 130.0, 130.0, 128.5, 127.1, 126.2, 125.7, 124.8, 123.4, 122.7, 121.9, 42.1, 32.4, 27.0, 26.4.

These spectroscopic data correspond to reported data.[5]

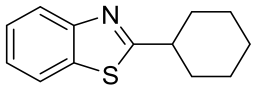

1-(4-(4-chlorobenzyl)phenyl)ethan-1-one (6q). 6q was synthesized following the

general procedure. The residue was purified by chromatography on silica gel, eluting with hexane/ethyl acetate (10:1) to give the title compound as colorless oil (26.6 mg, 61%).

6q: ^{1}H NMR (400 MHz, $CDCl_3$) δ 7.97 (d, *J* = 8.1 Hz, 1H), 7.84 (d, *J* = 7.9 Hz, 1H), 7.43 (t, *J* = 7.7 Hz, 1H), 7.32 (t, *J* = 7.6 Hz, 1H), 3.10 (tt, *J* = 11.7, 3.7 Hz, 1H), 2.25 – 2.16 (m, 2H), 1.93 – 1.84 (m, 2H), 1.80 – 1.72 (m, 1H), 1.70 – 1.58 (m, 2H), 1.51 – 1.38 (m, 2H), 1.33 (tt, *J* = 13.1, 3.8 Hz, 1H).^{13}C NMR (101 MHz, $CDCl_3$) δ 177.7, 153.2, 134.7, 125.9, 124.6, 122.7, 121.7, 43.6, 33.5, 26.2, 25.9.

These spectroscopic data correspond to reported data.[9]

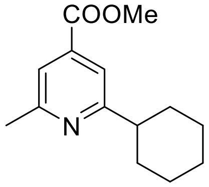

Methyl 2-cyclohexyl-6-methylisonicotinate (6r). 6r was synthesized following the general procedure. The residue was purified by chromatography on silica gel, eluting with hexane/ethyl acetate (5:1) to give the title compound as colorless oil (25.1 mg, 54%).

6r: ^{1}H NMR (400 MHz, $CDCl_3$) δ 7.50 (s, 2H), 3.92 (s, 3H), 2.74 (tt, *J* = 11.8, 3.3 Hz, 1H), 2.58 (s, 3H), 1.99 – 1.92 (m, 2H), 1.88 – 1.81 (m, 2H), 1.78 – 1.71 (m, 1H), 1.55 – 1.35 (m, 4H), 1.33 – 1.26 (m, 1H). ^{13}C NMR (101 MHz, $CDCl_3$) δ 167.4, 166.5, 158.7, 138.0, 119.9, 117.0, 52.6, 46.8, 33.1, 26.6, 26.1, 24.7.

These spectroscopic data correspond to reported data.[5]

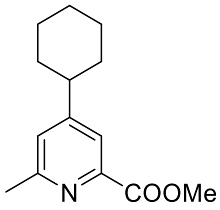

Methyl 4-cyclohexyl-6-methylpicolinate (6s). 6s was synthesized following the general procedure. The residue was purified by chromatography on silica gel, eluting with hexane/ethyl acetate (5:1) to give the title compound as pale yellow oil (31.0 mg, 67%).

6s: ^{1}H NMR (400 MHz, $CDCl_3$) δ 7.78 (s, 1H), 7.13 (s, 1H), 3.95 (s, 3H), 2.58 (s, 3H), 2.54 – 2.45 (m, 1H), 1.88 – 1.77 (m, 4H), 1.76 – 1.69 (m, 1H), 1.45 – 1.29 (m, 4H), 1.28 – 1.17 (m, 1H). ^{13}C NMR (101 MHz, $CDCl_3$) δ 165.5, 159.5, 158.6, 146.4, 126.1, 121.8, 53.1, 44.0, 33.4, 26.4, 25.8, 24.2.

HRMS (ESI/QTOF) m/z: $[M + H]^+$ Calcd for $C_{14}H_{20}NO_2^+$ 234.1489; Found 234.1491.

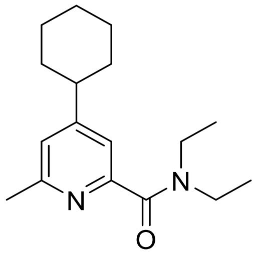

4-cyclohexyl-*N,N*-diethyl-6-methylpicolinamide (6t). 6t was synthesized following the general procedure. The residue was purified by chromatography on silica gel, eluting with hexane/ethyl acetate (2:1) to give the title compound as colorless oil (27.6 mg, 50%).

6t: ^{1}H NMR (400 MHz, $CDCl_3$) δ 7.15 (s, 1H), 6.98 (s, 1H), 3.53 (q, *J* = 7.1 Hz, 2H), 3.29 (q, *J* = 7.1 Hz, 2H), 2.42 – 2.57 (m, 4H), 1.88 – 1.79 (m, 4H), 1.77 – 1.70 (m, 1H), 1.42 – 1.30 (m, 4H), 1.28 – 1.19 (m, 4H), 1.13 (t, *J* = 7.1 Hz, 3H). ^{13}C NMR (101 MHz, $CDCl_3$) δ 169.5, 157.8, 157.4, 154.7, 122.3, 118.4, 44.0, 43.3, 40.2, 33.5, 26.6, 26.0, 24.4, 14.3, 13.0.

HRMS (ESI/QTOF) m/z: $[M + H]^+$ Calcd for $C_{17}H_{27}N_2O^+$ 275.2118; Found 275.2114.

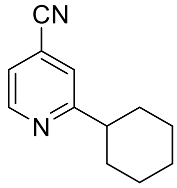

2-cyclohexylisonicotinonitrile (6u). 6u was synthesized following the general procedure. The residue was purified by chromatography on silica gel, eluting with hexane/ethyl acetate (10:1) to give the title compound as colorless oil (15.3 mg, 41%).

6u: ^{1}H NMR (400 MHz, $CDCl_3$) δ 8.69 (d, *J* = 5.0 Hz, 1H), 7.37 (s, 1H), 7.32 (d, *J* = 5.0 Hz, 1H), 2.75 (tt, *J* = 11.7, 3.6 Hz, 1H), 1.98 – 1.91 (m, 2H), 1.90 – 1.83 (m, 2H), 1.79 – 1.75 (m, 1H), 1.56 – 1.36 (m, 4H), 1.33 – 1.24 (m, 1H). ^{13}C NMR (101 MHz, $CDCl_3$) δ 168.3, 150.2, 123.0, 122.5, 120.7, 117.0, 46.5, 32.7, 26.4, 26.0.

These spectroscopic data correspond to reported data.[10]

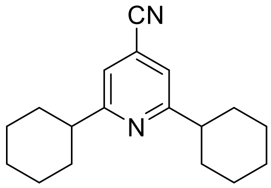

2,6-dicyclohexylisonicotinonitrile (6u’). 6u’ was synthesized following the general procedure. The residue was purified by chromatography on silica gel, eluting with hexane/ethyl acetate (10:1) to give the title compound as colorless oil (10.5 mg, 20%).

6u’: ^{1}H NMR (400 MHz, $CDCl_3$) δ 7.16 (s, 2H), 2.77 – 2.67 (m, 2H), 1.98 – 1.91 (m, 4H), 1.89 – 1.82 (m, 4H), 1.80 – 1.72 (m, 2H), 1.54 – 1.36 (m, 8H), 1.34 – 1.25 (m, 2H). 13C NMR (101 MHz, $CDCl_3$) δ 167.4, 120.7, 119.8, 117.7, 46.5, 32.8, 26.5, 26.1.

HRMS (ESI/QTOF) m/z: $[M + H]^+$ Calcd for $C_{18}H_{25}N_2^+$ 269.2012; Found 269.2012.

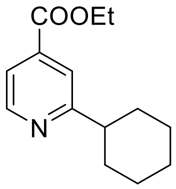

Methyl 2-cyclohexylisonicotinate (6v). 6v was synthesized following the general procedure. The residue was purified by chromatography on silica gel, eluting with hexane/ethyl acetate (10:1) to give the title compound as colorless oil (22.2 mg, 47%).

6v: ^{1}H NMR (400 MHz, $CDCl_3$) δ 8.67 (d, *J* = 5.0 Hz, 1H), 7.72 (s, 1H), 7.65 (d, *J* = 5.0 Hz, 1H), 4.41 (q, *J* = 7.1 Hz, 2H), 2.79 (tt, *J* = 12.0, 3.6 Hz, 1H), 2.01 – 1.94 (m, 2H), 1.92 – 1.84 (m, 2H), 1.81 – 1.73 (m, 1H), 1.91 – 1.51 (m, 2H), 1.49 – 1.38 (m, 5H), 1.36 – 1.27 (m, 1H). ^{13}C NMR (101 MHz, $CDCl_3$) δ 167.8, 165.7, 149.9, 138.2, 120.5, 120.3, 61.8, 46.7, 32.9, 26.6, 26.1, 14.3.

These spectroscopic data correspond to reported data.[11]

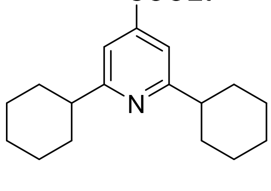

Methyl 2,6-dicyclohexylisonicotinate (6v’). 6v’ was synthesized following the

general procedure. The residue was purified by chromatography on silica gel, eluting with hexane/ethyl acetate (10:1) to give the title compound as colorless oil (19.9 mg, 32%).

6v: ^{1}H NMR (400 MHz, $CDCl_3$) δ 7.52 (s, 2H), 4.41 (q, *J* = 7.1 Hz, 2H), 2.77 (tt, *J* = 11.8, 3.5 Hz, 2H), 2.03 – 1.94 (m, 4H), 1.91 – 1.83 (m, 4H), 1.81 – 1.73 (m, 2H), 1.60 – 1.48 (m, 4H), 1.47 – 1.39 (m, 6H), 1.37 – 1.28 (m, 2H). ^{13}C NMR (101 MHz, $CDCl_3$) δ 166.9, 166.3, 138.4, 117.3, 61.6, 46.7, 33.1, 26.7, 26.2, 14.4.

These spectroscopic data correspond to reported data.[6]

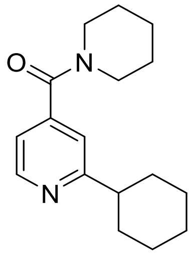

(2-cyclohexylpyridin-4-yl)(piperidin-1-yl)methanone (6w). 6w was synthesized following the general procedure. The residue was purified by chromatography on silica gel, eluting with hexane/ethyl acetate (2:1) to give the title compound as colorless oil (21.8 mg, 40%).

6w: ^{1}H NMR (400 MHz, $CDCl_3$) δ 8.56 (d, *J* = 5.0 Hz, 1H), 7.12 (s, 1H), 7.05 (dd, *J* = 5.0, 1.5 Hz, 1H), 3.69 (t, *J* = 5.4 Hz, 2H), 3.25 (t, *J* = 5.5 Hz, 2H), 2.71 (tt, *J* = 11.8, 3.5 Hz, 1H), 1.98 – 1.90 (m, 2H), 1.88 – 1.80 (m, 2H), 1.77 – 1.61 (m, 5H), 1.56 – 1.33 (m, 6H), 1.31 – 1.23 (m, 1H). ^{13}C NMR (101 MHz, $CDCl_3$) δ 168.2, 167.3, 149.4, 144.7, 118.5, 118.4, 48.6, 46.6, 43.1, 32.9, 26.6, 26.1, 25.6, 24.5.

HRMS (ESI/QTOF) m/z: $[M + H]^+$ Calcd for $C_{17}H_{25}N_2O^+$ 273.1961; Found 273.1961

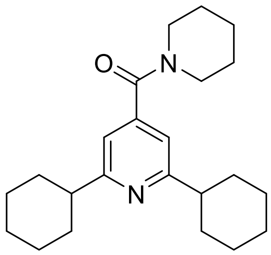

(2,6-dicyclohexylpyridin-4-yl)(piperidin-1-yl)methanone (6w’). 6w’ was synthesized following the general procedure. The residue was purified by

chromatography on silica gel, eluting with hexane/ethyl acetate (2:1) to give the title compound as colorless oil (6.0 mg, 8%).

6w': ^{1}H NMR (400 MHz, $CDCl_3$) δ 6.91 (s, 1H), 3.71 (t, *J* = 5.0 Hz, 2H), 3.26 (t, *J* = 5.5 Hz, 2H), 2.74 – 2.65 (m, 2H), 1.99 – 1.92 (m, 4H), 1.86 – 1.79 (m, 4H), 1.77 – 1.65 (m, 6H), 1.52 – 1.25 (m, 12H). ^{13}C NMR (101 MHz, $CDCl_3$) δ 169.1, 166.4, 144.8, 115.2, 48.7, 46.7, 43.1, 33.1, 26.7, 26.3, 25.7, 24.7.

HRMS (ESI/QTOF) m/z: $[M + H]^+$ Calcd for $C_{23}H_{35}N_2O^+$ 355.2744; Found 355.2741

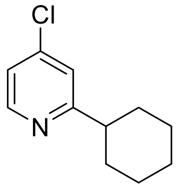

4-chloro-2-cyclohexylpyridine (6x). 6x was synthesized following the general procedure. The residue was purified by chromatography on silica gel, eluting with hexane/ethyl acetate (10:1) to give the title compound as colorless oil (19.2 mg, 49%).

6x: ^{1}H NMR (400 MHz, $CDCl_3$) δ 8.41 (d, *J* = 5.3 Hz, 1H), 7.16 (d, *J* = 2.0 Hz, 1H), 7.13 (dd, *J* = 5.3, 2.0 Hz, 1H)., 2.68 (tt, *J* = 11.8, 3.4 Hz, 1H), 1.97 – 1.90 (m, 2H), 1.89 – 1.82 (m, 2H), 1.78 – 1.71 (m, 1H), 1.54 – 1.34 (m, 4H), 1.32 – 1.25 (m, 1H). ^{13}C NMR (101 MHz, $CDCl_3$) δ 168.4, 150.1, 144.4, 121.6, 121.5, 46.5, 32.8, 26.6, 26.1.

These spectroscopic data correspond to reported data.[12]

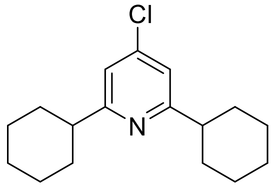

4-chloro-2,6-dicyclohexylpyridine (6x'). 6x' was synthesized following the general procedure. The residue was purified by chromatography on silica gel, eluting with hexane/ethyl acetate (10:1) to give the title compound as colorless oil (10.1 mg, 18%).

6x': ^{1}H NMR (400 MHz, $CDCl_3$) δ 6.95 (s, 2H), 2.66 (tt, *J* = 11.6, 3.4 Hz, 2H), 1.99 – 1.91 (m, 4H), 1.87 – 1.79 (m, 4H), 1.77 – 1.70 (m, 2H), 1.51 – 1.34 (m, 8H), 1.32 – 1.23 (m, 2H). ^{13}C NMR (101 MHz, $CDCl_3$) δ 167.5, 144.3, 118.3, 46.6, 33.0, 26.6, 26.2.

These spectroscopic data correspond to reported data.[12]

3. UV-vis Spectra

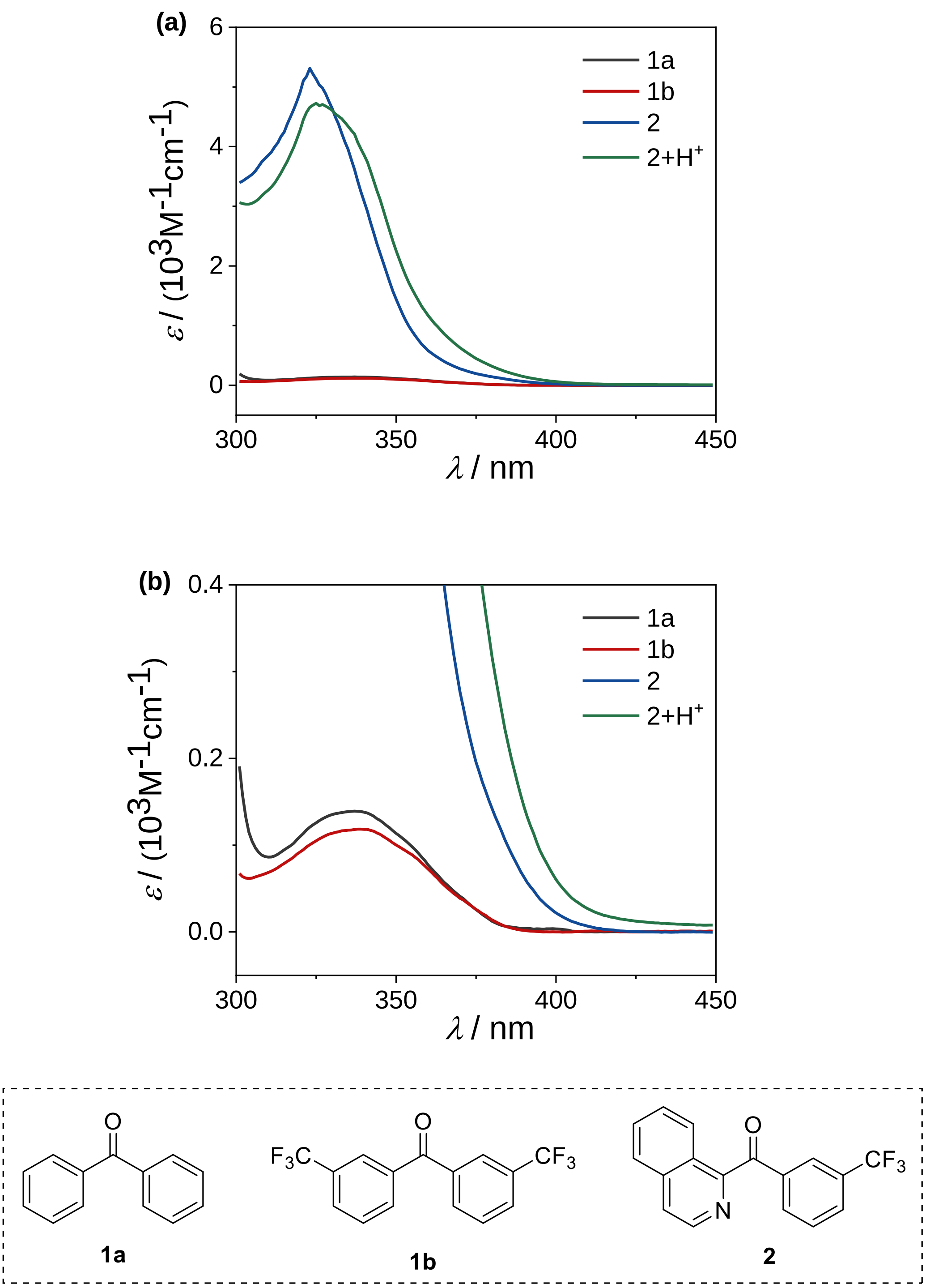

Figure S1. (a) UV-vis spectra of several representative diaryl ketone-type photocatalysts (0.5 mM in CH_3CN): **1a**. benzophenone; **1b**. trifluoromethyl-substituted benzophenone; **2**. the present photocatalyst. (b) Zoom-in of Fig. S1(a) showing the absorption of **1a** and **1b** on a magnified y-scale.

4. Time Dependent Spectroscopic Measurements

An LP920-KS apparatus from Edinburgh Instruments was used for transient absorption spectroscopy, using 355 nm and 410 nm as excitation wavelengths. For the excitation with 355 nm, a Q-smart 450 pulsed laser (10 ns pulse width, 12 mJ) from Lumibird was used, whereas excitation at 410 nm was achieved by a Quantel Brilliant laser (10 ns pulse duration, 6 mJ), which was equipped with an optical parametric oscillator (OPO) from Opotek. The transient absorption spectra were detected with an iCCD camera from Andor, whereas the ESA kinetics at single wavelengths were recorded using a photomultiplier tube. All laser flash photolysis experiments were carried out at 293 K. For some measurements under de-aerated conditions, the samples were purged with Argon (4.6, PanGas, 1 atm) for five minutes and then sealed with a septum cap.

The excited state absorption (ESA) spectra and the temporal evolution of the ESA of the neutral **2** (Figure S2a - c) and protonated **2H$^+$** (Figure S2d - f, addition of 200 mM CF_3COOH) sensitizer were measured in acetonitrile upon 355 nm excitation with laser pulses (12 mJ, 10 Hz). The neutral sensitizer **2** shows an ESA band at 525 nm with a shoulder at 395 nm (Figure S2a). The protonated sensitizer **2H$^+$** (Figure S2d) shows somewhat red-shifted ESA bands at 535 nm and 402 nm. Furthermore, a ground state absorption (GSA) bleach at 360 nm was observed for **2H$^+$**, matching with the absorption band at 355 nm (Figure S2a, blue solid line). The kinetic traces monitoring the ESA band (at 530 nm) of **2** are shown in Figure S2b (air–saturated) and c (argon–saturated), whereas analogous data sets for **2H$^+$** are shown in Figure S2e (air–saturated) and f (argon–saturated). The sensitizer **2** shows an ESA lifetime of 340 ns under air (Figure S2b), whereas **2H$^+$** exhibits a slightly longer lifetime of 470 ns (Figure S2e). The same trend was observed under argon-saturated conditions, where **2** exhibits a triplet lifetime of 9.66 μs and **2H$^+$** of 37.4 μs. When comparing the excited-state decays of **2H$^+$** and **2** under aerobic and inert conditions, a large difference in excited state lifetime (up to a factor of 80) is observed. This drastic decrease in triplet lifetime when going from argon-saturated to air-saturated solution is attributable to quenching by oxygen, likely resulting in the formation singlet oxygen.

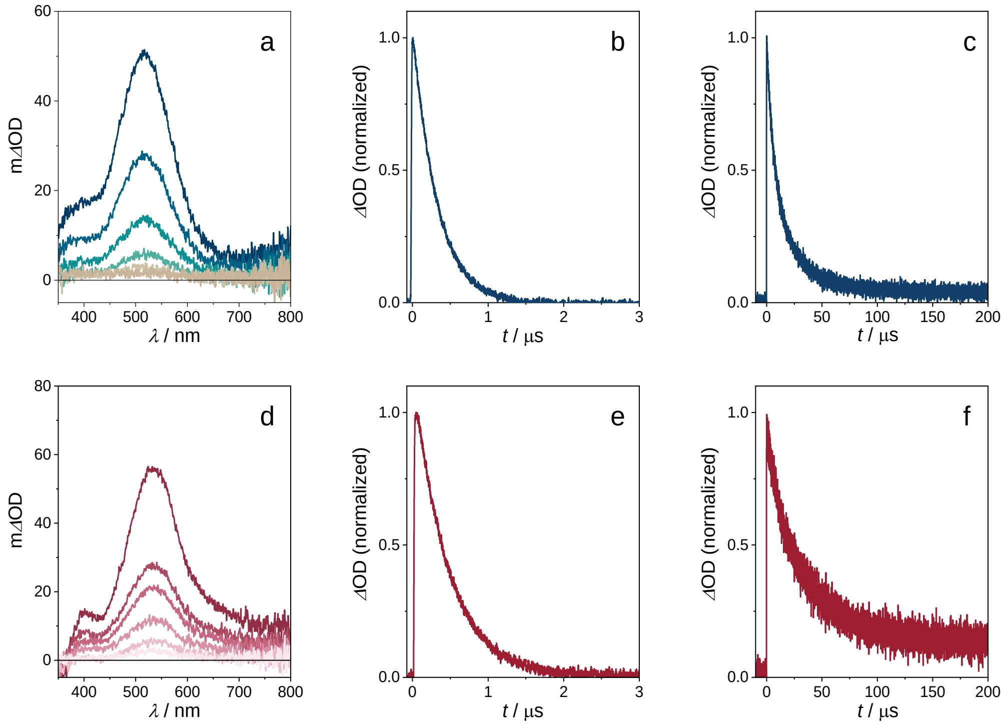

Figure S2. Spectroscopic investigation of the triplet excited state of the sensitizer in acetonitrile in absence of CF_3COOH (**2**, 300 µM, blue lines, a-c) and after the addition of 200 mM CF_3COOH (**2H$^+$**, 100 µM, red lines, d-f) by laser flash photolysis upon 355 nm pulsed excitation (12 mJ per pulse) at 20 °C. Panels a & d show the transient absorption spectra of **2** (a) and **2H$^+$** (d) under air recorded after different time delays (50 ns, 250 ns, 500 ns, 750 ns, 1000 ns 1500 ns). Panels b & e represents the kinetic decays of the excited state absorption at 530 nm under air, whereas panel c & f shows the kinetic decay of the excited triplet state under argon.

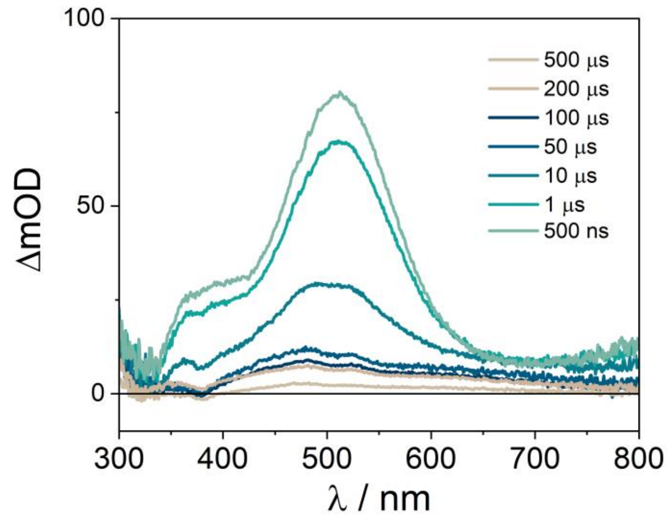

Figure S3. Transient absorption spectra of the triplet excited state of the sensitizer in acetonitrile with 200 mM CF_3COOH (**$2H^+$**, 100 μM). The spectra were recorded upon 355 nm pulsed excitation (15 mJ per pulse) at 20 °C under argon with different time delays (up to 500 μs) after excitation.

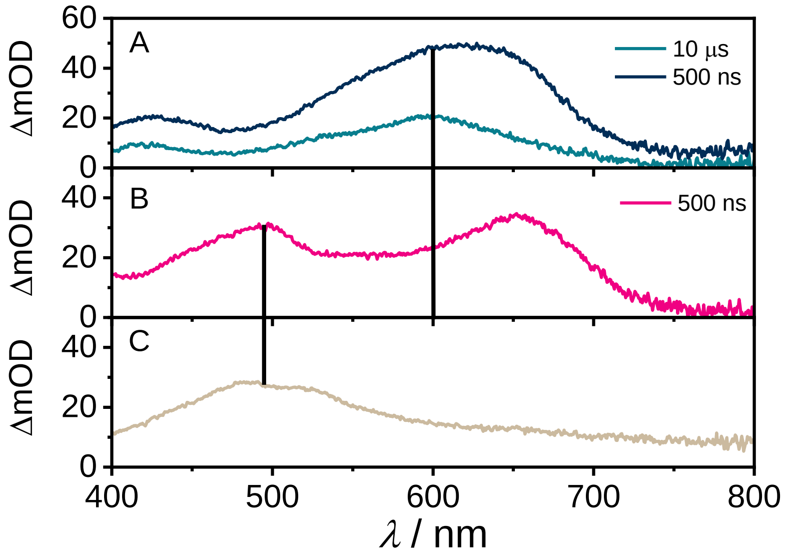

Figure S4. A) Transient absorption spectra of the sensitizer (200 µM) in acetonitrile without CF_3COOH (**2,** 200 µM) in the presence of 5 mM triphenylamine (TPA), recorded with two different time delays. B) Transient absorption spectrum of the sensitizer in acetonitrile with CF_3COOH (**2H**$^+$, 200 µM) in the presence of 5 mM TPA, recorded with a delay time of 500 ns. C) Transient absorption spectrum of the triplet excited state of the sensitizer in acetonitrile with 200 mM CF_3COOH (**2H**$^+$, 100 µM), recorded with time delay of 50 µs. All spectra were recorded upon 355 nm pulsed excitation (15 mJ per pulse) at 20 °C under argon.

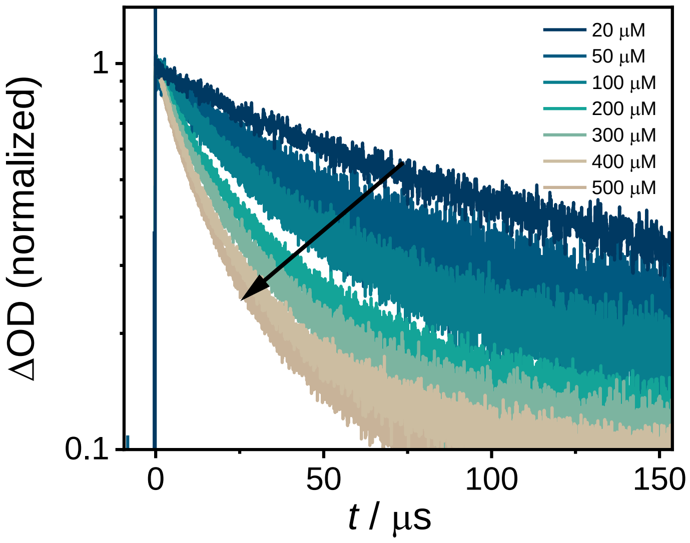

Figure S5. Excited state decay of **2H**$^+$ at different concentrations between 20 μM and 500 μM, recorded by monitoring the excited state absorption (ESA) signal at 540 nm after 355 nm pulsed excitation (20 mJ per pulse). All measurements occurred in the presence of 1500 mM CF_3COOH in deaerated CH_3CN at 20 °C.

5. Cyclic Voltammetry and Determination of Excited State Potential

Cyclic voltammograms were recorded on a Gamry Interface 1010E Potentiostat using a glassy carbon working electrode, a Pt counter electrode, and a saturated Ag/AgCl reference electrode. The voltammograms were measured at room temperature in a 0.1 M CH_3CN solution of $LiClO_4$ containing 3 mM of **2** and 6 mM of CF_3COOH. The potential scan rate was 100 mV/s. A internal Fc/Fc^+ reference couple was used.

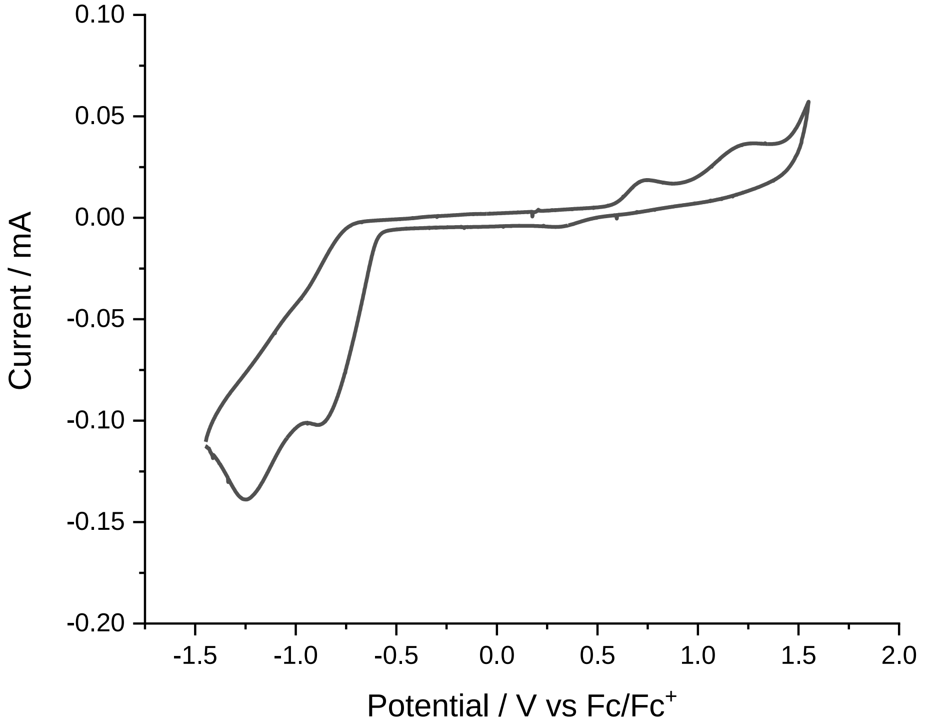

Figure S6: Cyclic voltammogram of **$2H^+$** in CH_3CN in presence of 6 mM CF_3COOH.

$E_{1/2}$ is approximated by E_{red} = ca. -0.71 V vs Fc/Fc^+

Excited state potentials are estimated using the Rehm-Weller equation as given[13]:

$$E_{1/2}(I^*/I^-) = E_{1/2}\ (I/I^-) + E^{0\text{-}0}$$

For conversion from Fc/Fc^+ to SCE, it is known that Fc/Fc^+ is 380 mV more positive than SCE in MeCN.[14]

$$E_{1/2}(I^*/I^-) = 2.18 \text{ V vs SCE}$$

6. Mass Spectrometry Studies

Mass spectrometry analyses were performed on an LTQ Orbitrap FTMS instrument (LTQ Orbitrap Elite FTMS, Thermo Scientific, Bremen, Germany) operated in the positive mode coupled with an APPI ionization source with a VUV Kr lamp (Syagen, Tustin, CA, USA) used with the commercial Thermo Scientific Ion Max APCI/APPI source bodies. A standard data acquisition and instrument control system was utilized (Thermo Scientific). Typical nebulizer temperature was fixed at 350°C whereas the infusion rate was 10 µL/min. The temperature of ion transfer capillary was 120 °C. FTMS spectra were obtained in the 100-200 *m/z* range in the reduce profile mode with a resolution set to 120,000. In all spectra, 10 microscan was acquired with a maximum injection time value of 1000 ms. Data treatment were performed using the Mass spectra Deconvolution free software created by the EPFL (https://ms.epfl.ch/applications/mass-spectra-deconvolution/).

Table S1. The mass spectrometry in the presence of deuterated cyclohexane with APPI as the ion source.

List of the various species

Part 1	Part 2	Part 3	Part 4	MF	Charge	Exp MS	Percent	Amount
	H^+	C_9H_7N		C9H8N(+1)	1	130.0651	96.97%	15735393...
$((H_{-1}D))$	H^+	C_9H_7N		C9H7[2H]N(+1)	1	131.0714	3.01%	488288.510
$((H_{-1}D))_2$	H^+	C_9H_7N		C9H6[2H]2N(+1)	1	132.0777	0.03%	4129.021
$((H_{-1}D))_3$	H^+	C_9H_7N		C9H5[2H]3N(+1)	1	133.0840	0.00%	0.000
$((H_{-1}D))_4$	H^+	C_9H_7N		C9H4[2H]4N(+1)	1	134.0902	0.00%	0.000
$((H_{-1}D))_5$	H^+	C_9H_7N		C9H3[2H]5N(+1)	1	135.0965	0.00%	0.000

Table S2. The mass spectrometry in the presence of general cyclohexane with APPI as the ion source.

List of the various species

Part 1	Part 2	Part 3	Part 4	MF	Charge	Exp MS	Percent	Amount
	H^+	C_9H_7N		C9H8N(+1)	1	130.0651	100.00%	7383822....
$((H_{-1}D))$	H^+	C_9H_7N		C9H7[2H]N(+1)	1	131.0714	0.00%	139.549
$((H_{-1}D))_2$	H^+	C_9H_7N		C9H6[2H]2N(+1)	1	132.0777	0.00%	0.000
$((H_{-1}D))_3$	H^+	C_9H_7N		C9H5[2H]3N(+1)	1	133.0840	0.00%	0.000
$((H_{-1}D))_4$	H^+	C_9H_7N		C9H4[2H]4N(+1)	1	134.0902	0.00%	0.000
$((H_{-1}D))_5$	H^+	C_9H_7N		C9H3[2H]5N(+1)	1	135.0965	0.00%	0.000

Table S2. The mass spectrometry in the presence of deuterated cyclohexane with ESI as the ion source.

List of the various species

Part 1	Part 2	Part 3	Part 4	MF	Charge	Exp MS	Percent	Amount
	H^+	C_9H_7N		C9H8N(+1)	1	130.0651	99.94%	17661825...
$(H_{-1}D)$	H^+	C_9H_7N		C9H7[2H]N(+1)	1	131.0714	0.00%	0.000
$(H_{-1}D)_2$	H^+	C_9H_7N		C9H6[2H]2N(+1)	1	132.0777	0.03%	57389.908
$(H_{-1}D)_3$	H^+	C_9H_7N		C9H5[2H]3N(+1)	1	133.0840	0.00%	0.000
$(H_{-1}D)_4$	H^+	C_9H_7N		C9H4[2H]4N(+1)	1	134.0902	0.00%	4693.069
$(H_{-1}D)_5$	H^+	C_9H_7N		C9H3[2H]5N(+1)	1	135.0965	0.03%	49511.695

7. References

[1] S. Batori, G. Hajos, P. Sandor, A. Messmer, *J. Org. Chem.* **1989**, *54*, 3062-3068.

[2] G.-X. Li, X. Hu, G. He, G. Chen, *ACS Catal.* **2018**, *8*, 11847-11853.

[3] J. K. Matsui, D. N. Primer, G. A. Molander, *Chem. Sci.* **2017**, *8*, 3512-3522.

[4] S. Liu, A. Liu, Y. Zhang, W. Wang, *Chem. Sci.* **2017**, *8*, 4044-4050.

[5] H. Zhao, J. Jin, *Org. Lett.* **2019**, *21*, 6179-6184.

[6] C. Huang, J.-H. Wang, J. Qiao, X.-W. Fan, B. Chen, C.-H. Tung, L.-Z. Wu, *J. Org. Chem.* **2019**, *84*, 12904-12912.

[7] F. J. R. Klauck, M. J. James, F. Glorius, *Angew. Chem. Int. Ed.* **2017**, *56*, 12336-12339.

[8] X.-L. Lai, X.-M. Shu, J. Song, H.-C. Xu, *Angew. Chem. Int. Ed.* **2020**, *59*, 10626-10632.

[9] J. Dong, F. Yue, H. Song, Y. Liu, Q. Wang, *Chem. Commun.* **2020**, *56*, 12652-12655.

[10] J. Zhou, Y. Zou, P. Zhou, Z. Chen, J. Li, *Org. Chem. Front.* **2019**, *6*, 1594-1598.

[11] C.-Y. Huang, J. Li, W. Liu, C.-J. Li, *Chem. Sci.* **2019**, *10*, 5018-5024.

[12] T. C. Sherwood, N. Li, A. N. Yazdani, T. G. M. Dhar, *J. Org. Chem.* **2018**, *83*, 3000-3012.

[13] J. L. Brennan, T. E. Keyes, R. J. Forster, *Langmuir*. **2006**, *22*, 10754.

[14] N. G. Connelly, W. E.Geiger, *Chem. Rev.* **1996**, *96*, 877.

8. NMR Spectra

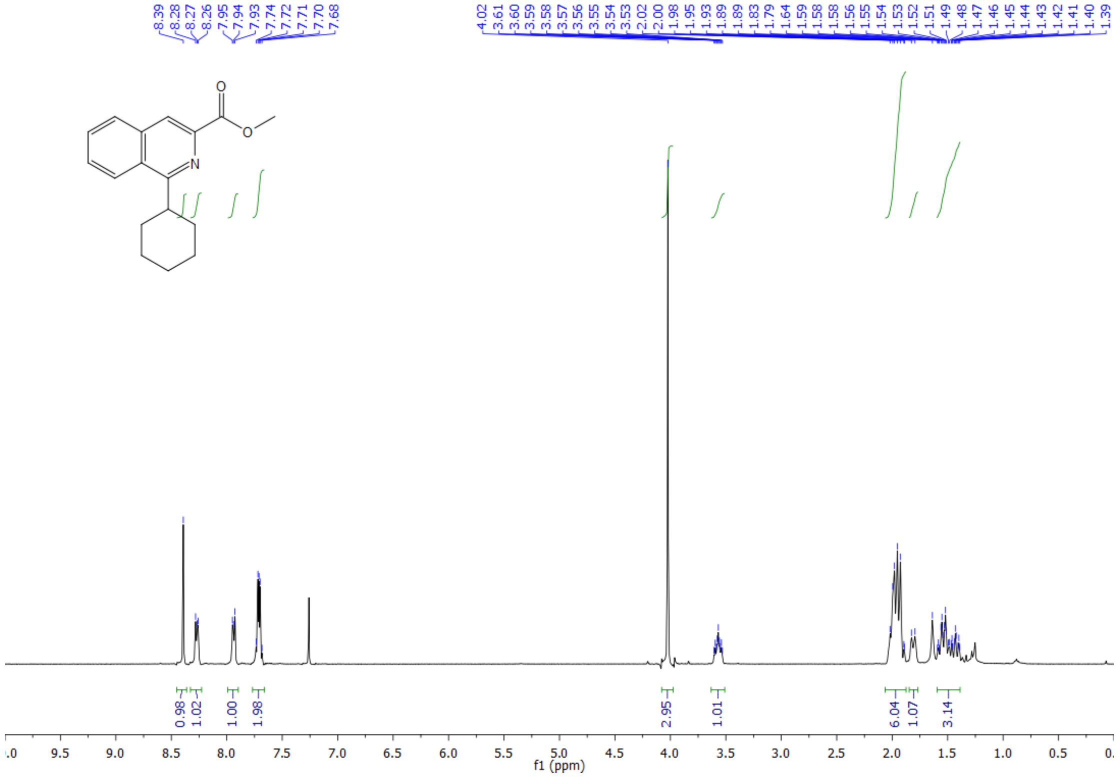

^{1}H NMR (400 MHz, $CDCl_3$) of **5a**

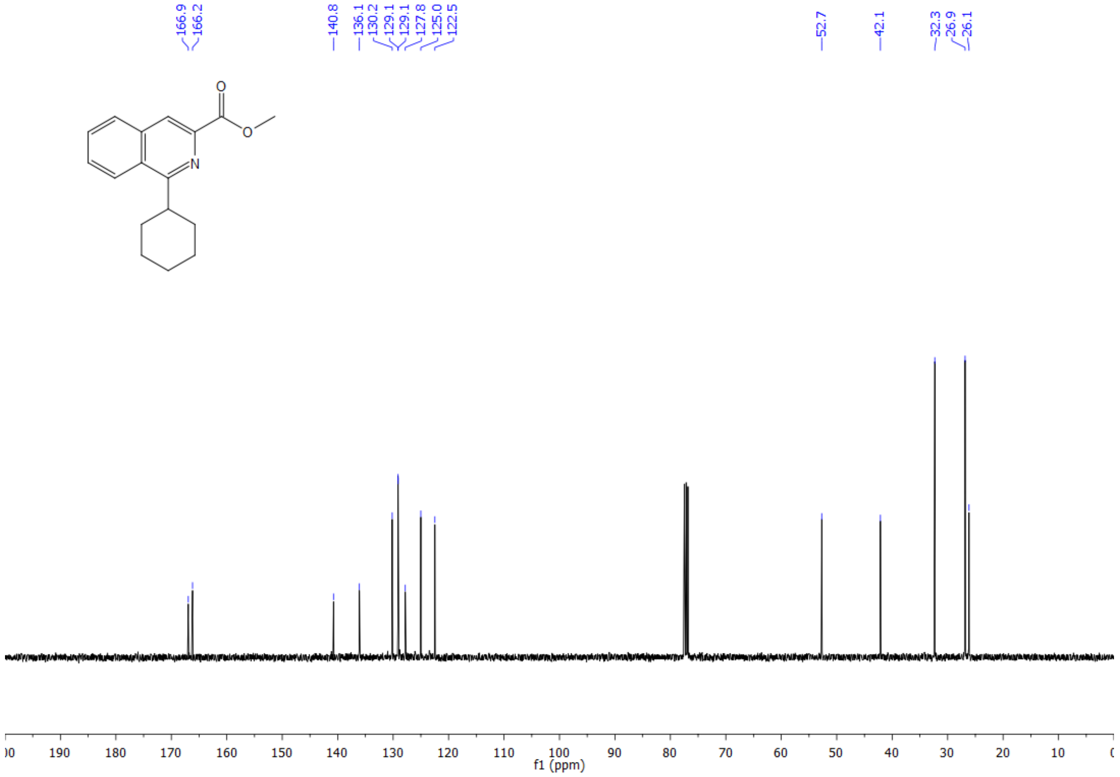

^{13}C NMR (101 MHz, $CDCl_3$) of **5a**

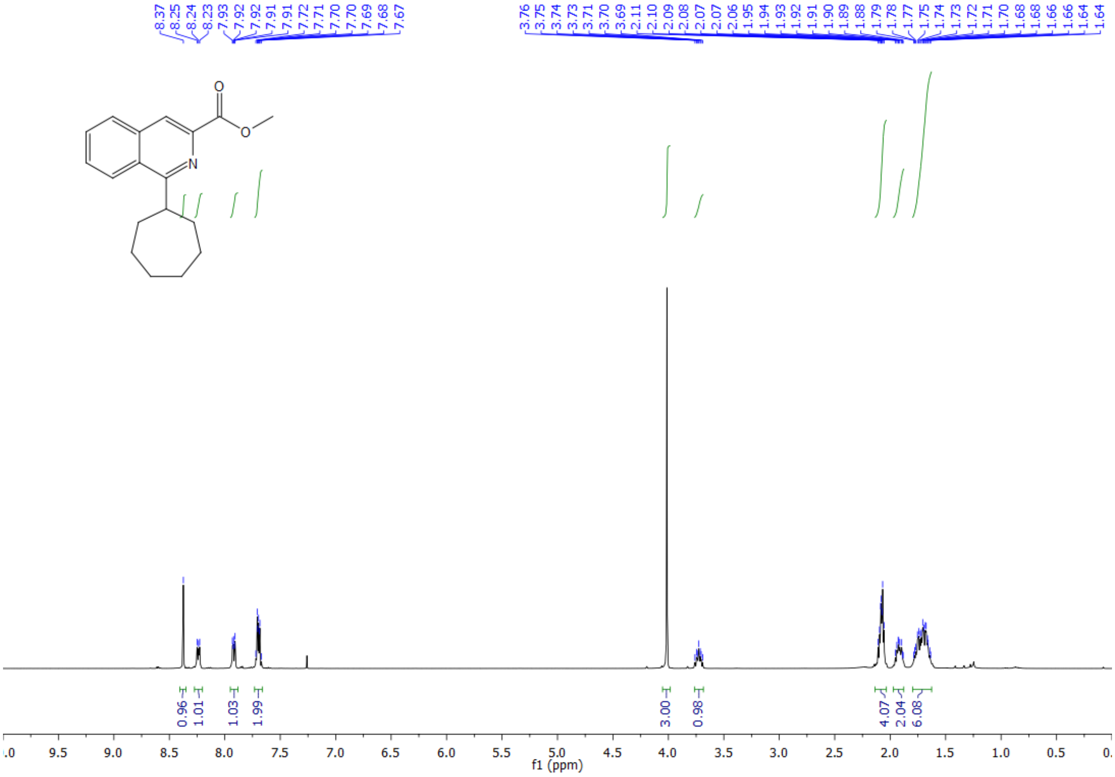

^{1}H NMR (400 MHz, $CDCl_3$) of **5b**

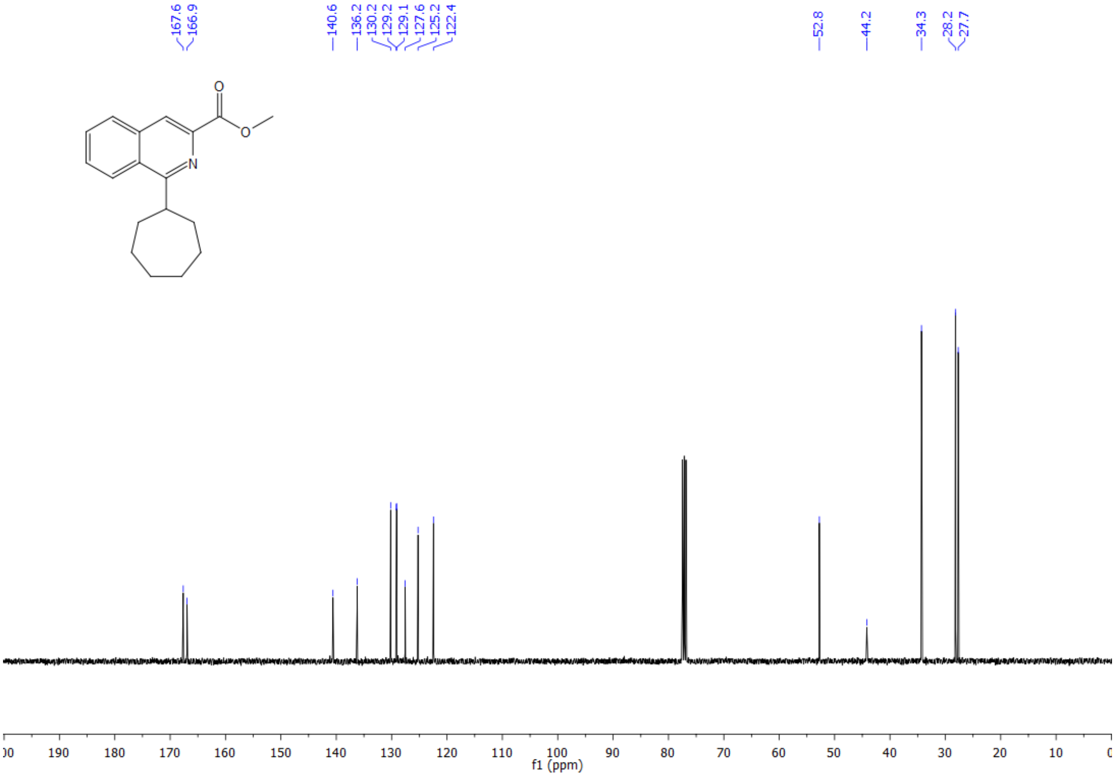

^{13}C NMR (101 MHz, $CDCl_3$) of **5b**

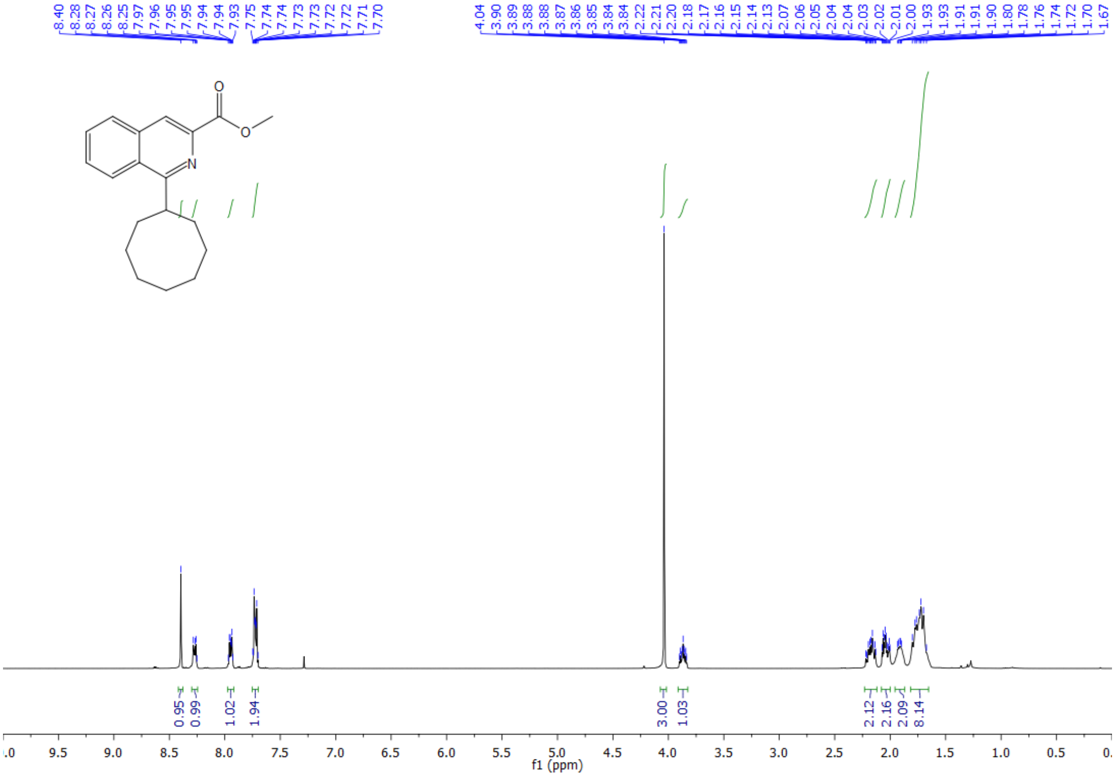

^{1}H NMR (400 MHz, $CDCl_3$) of **5c**

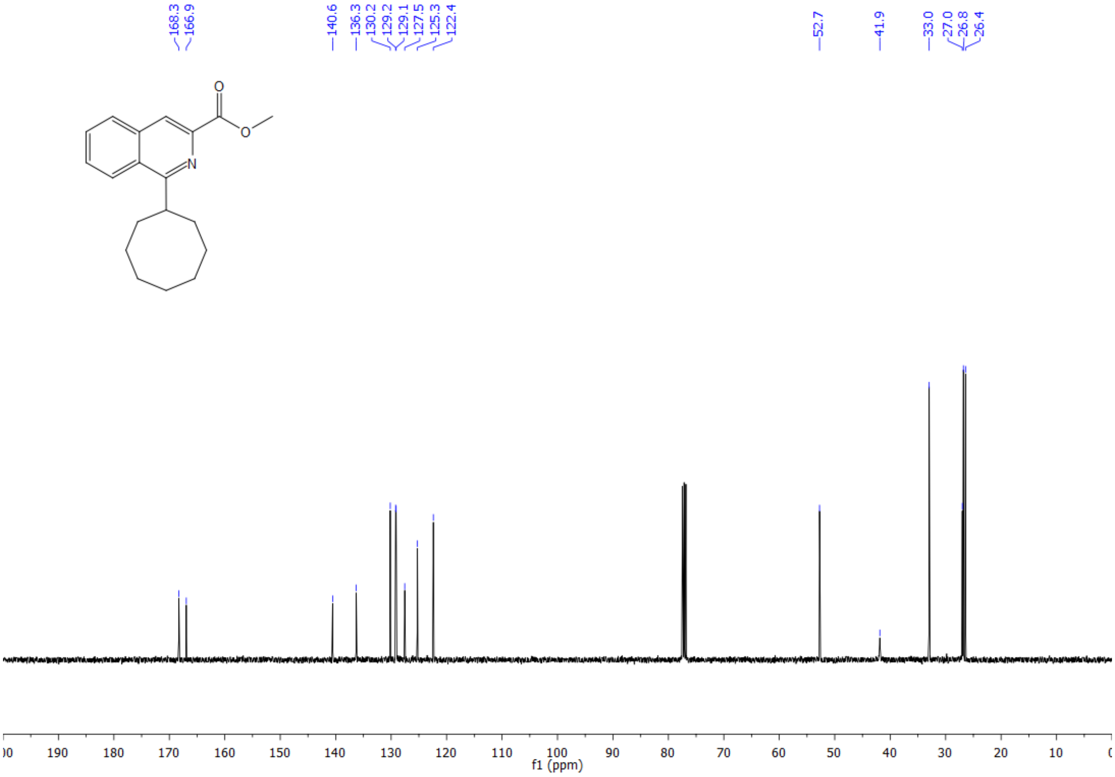

^{13}C NMR (101 MHz, $CDCl_3$) of **5c**

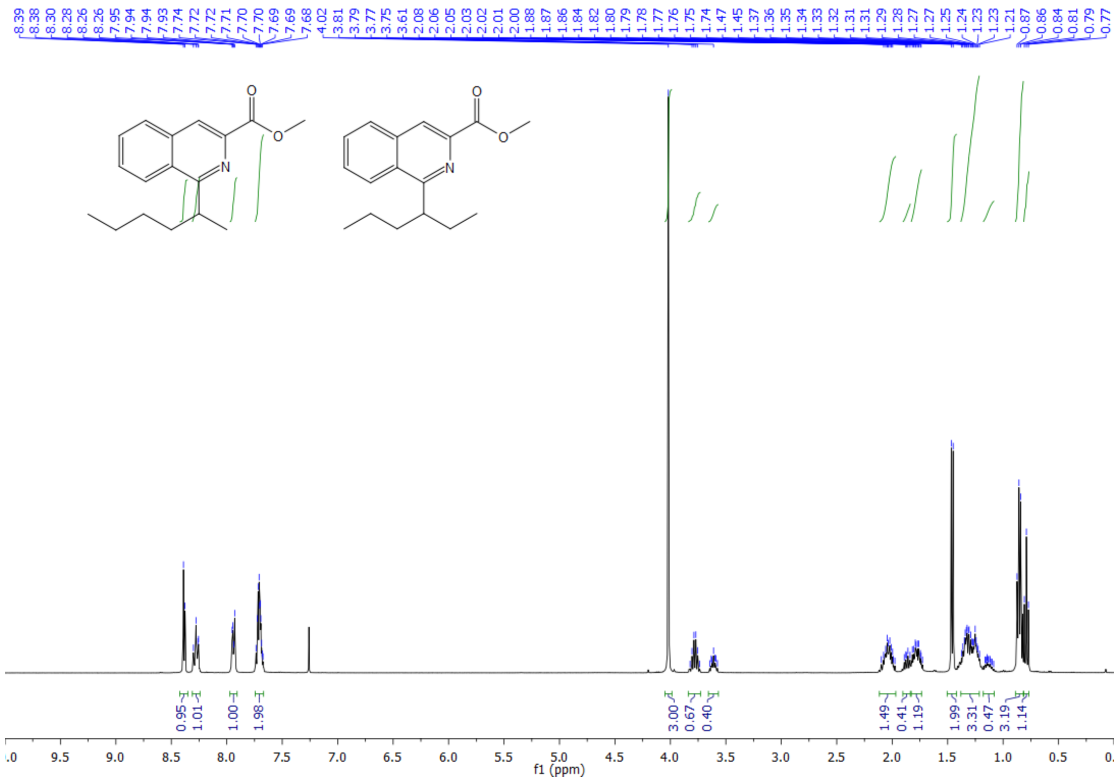

^{1}H NMR (400 MHz, $CDCl_3$) of **5d+5d'**

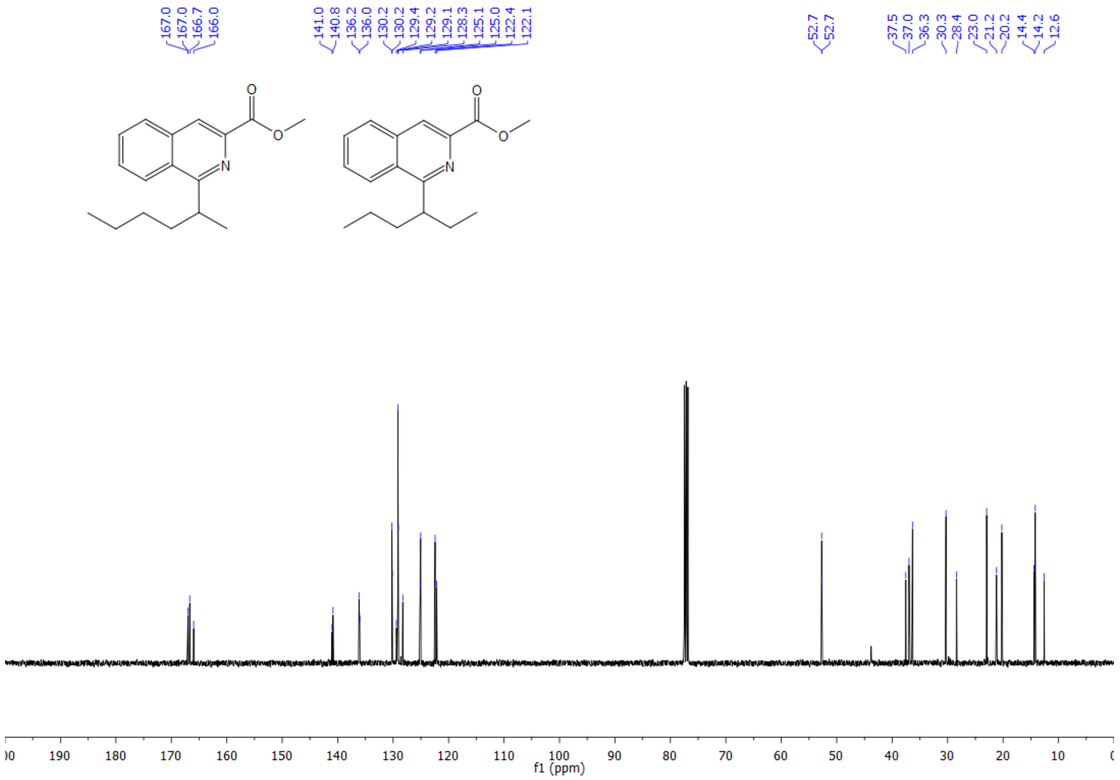

^{13}C NMR (101 MHz, $CDCl_3$) of **5d+5d'**

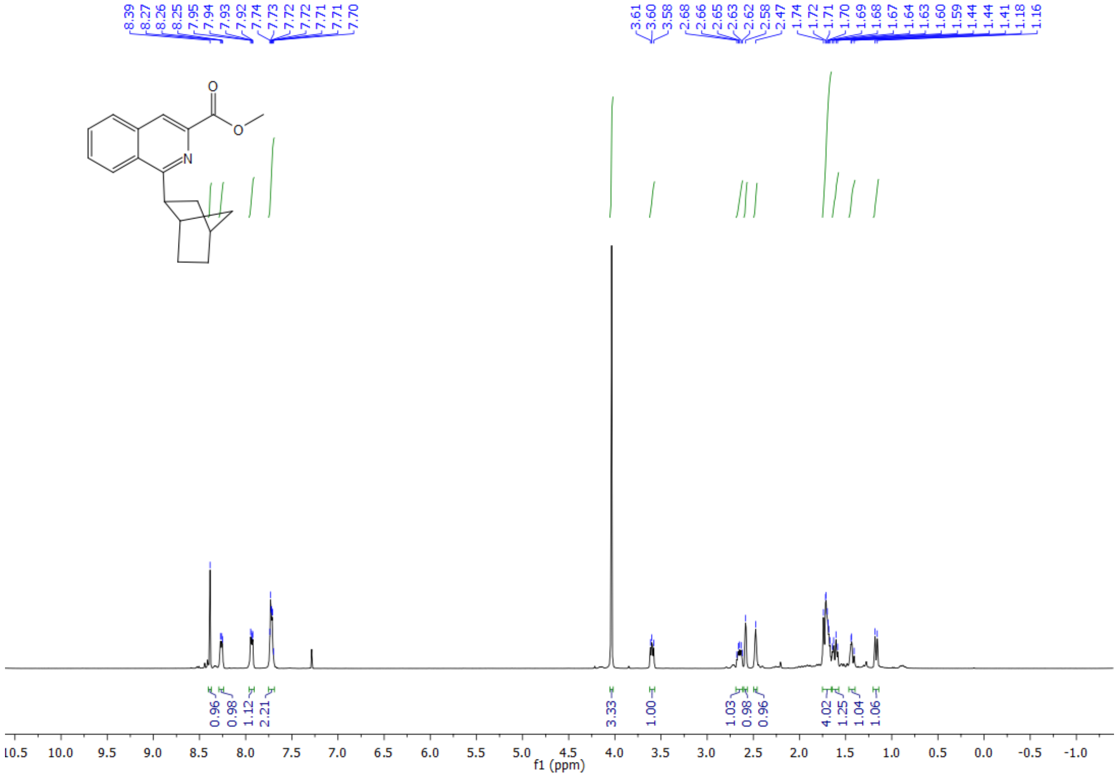

^{1}H NMR (400 MHz, $CDCl_3$) of **5e**

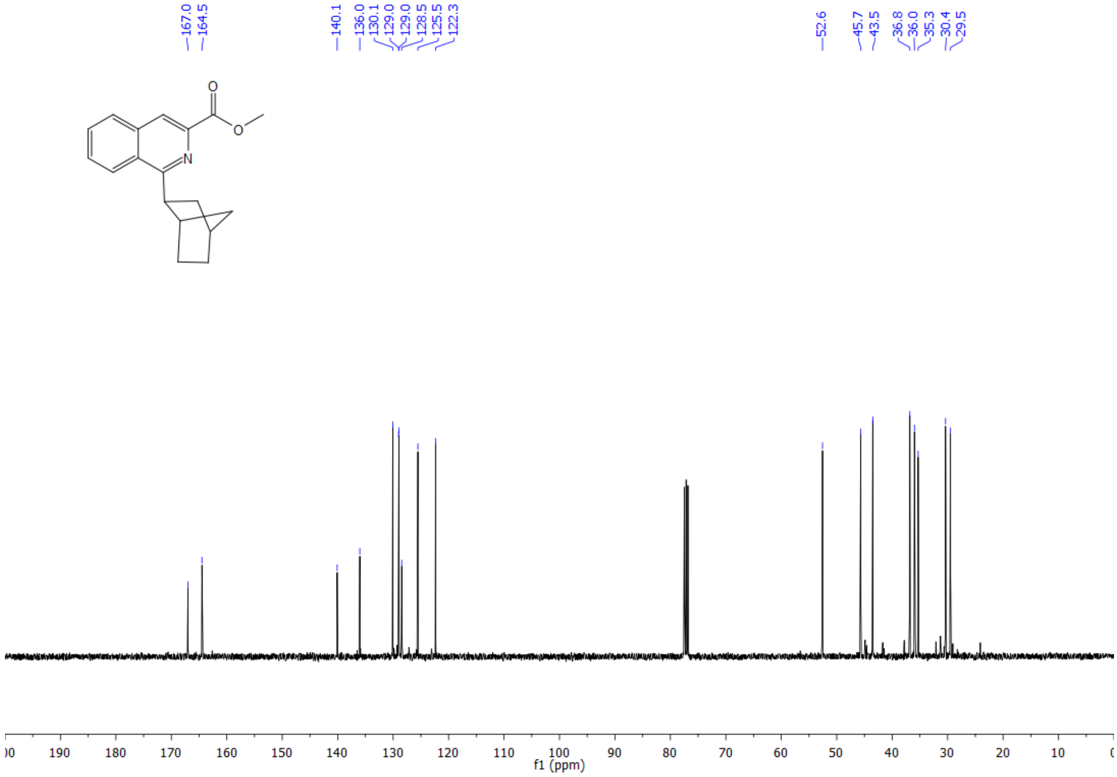

^{13}C NMR (101 MHz, $CDCl_3$) of **5e**

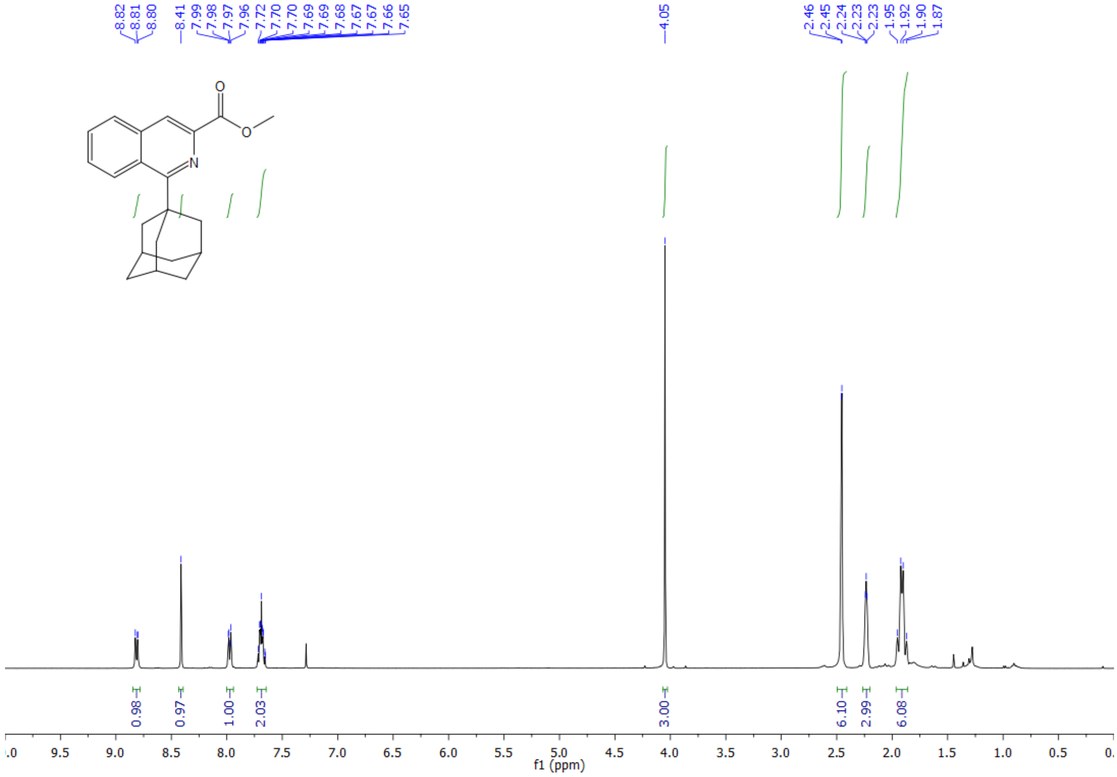

^{1}H NMR (400 MHz, CDCl$_3$) of **5f**

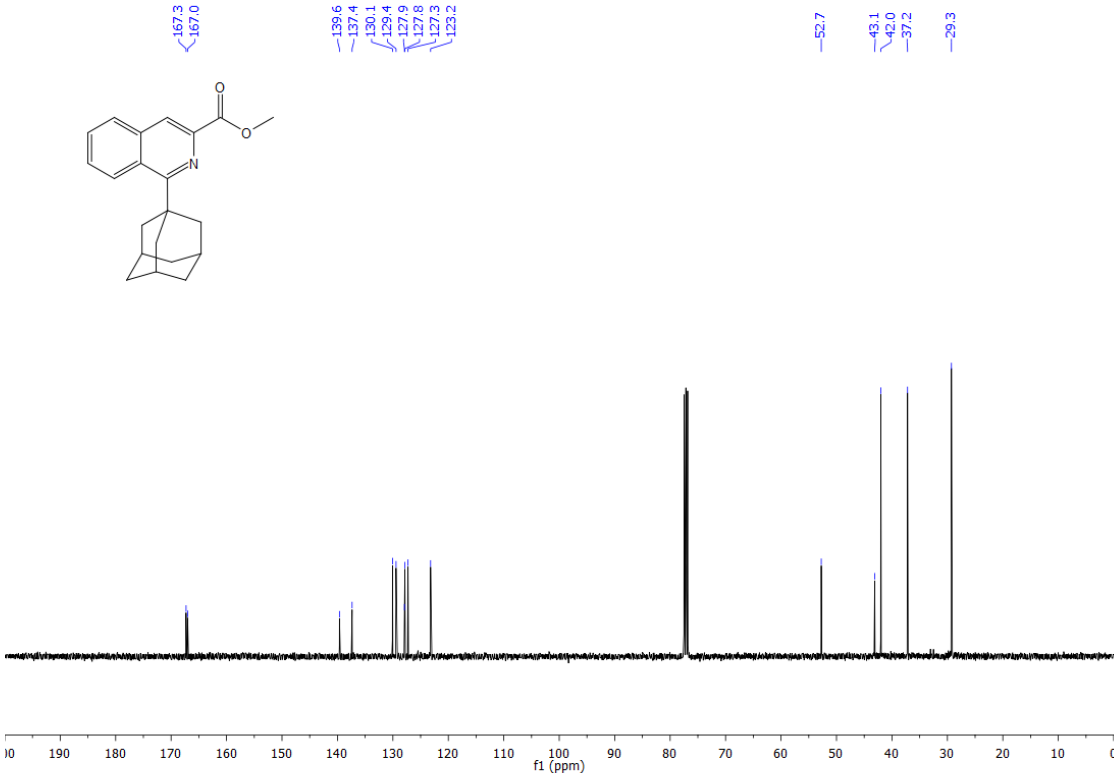

^{13}C NMR (101 MHz, CDCl$_3$) of **5f**

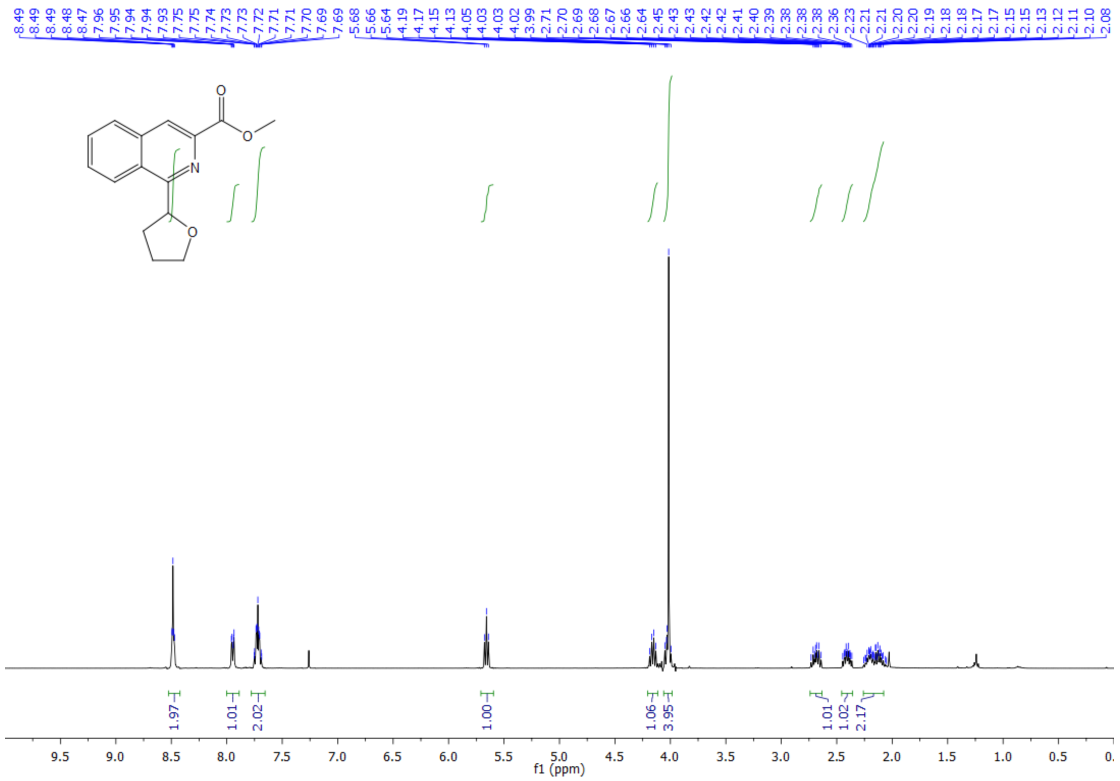

^{1}H NMR (400 MHz, CDCl$_3$) of **5g**

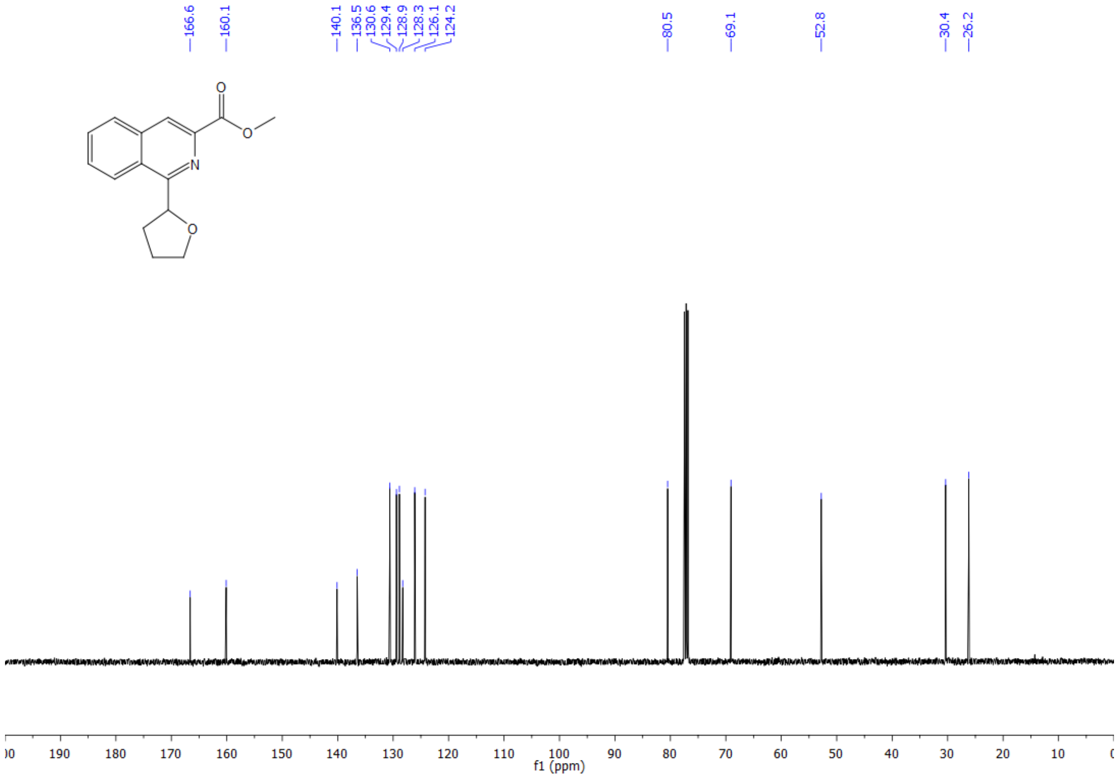

^{13}C NMR (101 MHz, CDCl$_3$) of **5g**

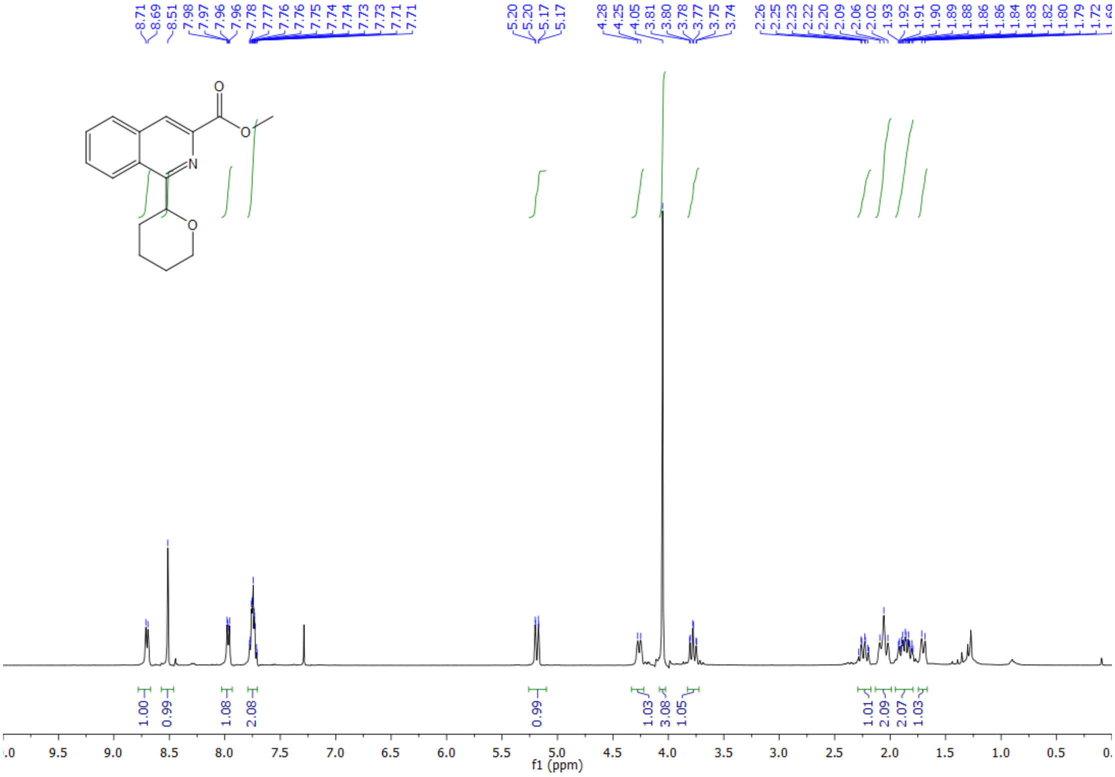

^{1}H NMR (400 MHz, $CDCl_3$) of **5h**

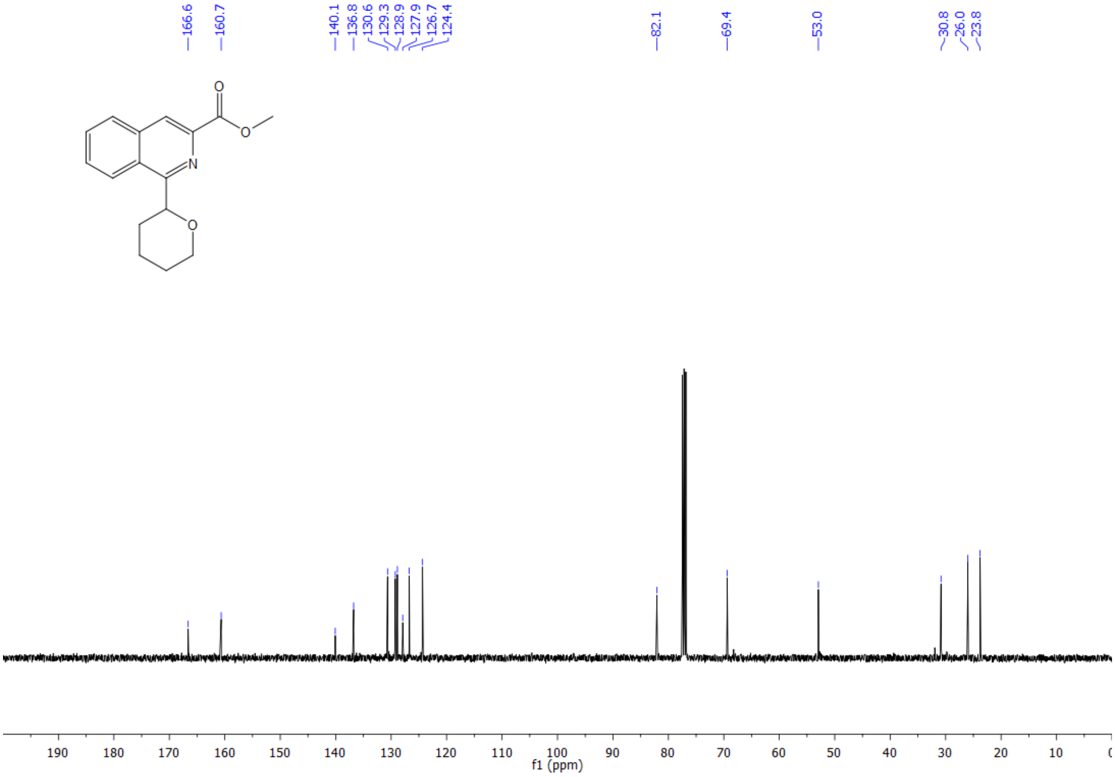

^{13}C NMR (101 MHz, $CDCl_3$) of **5h**

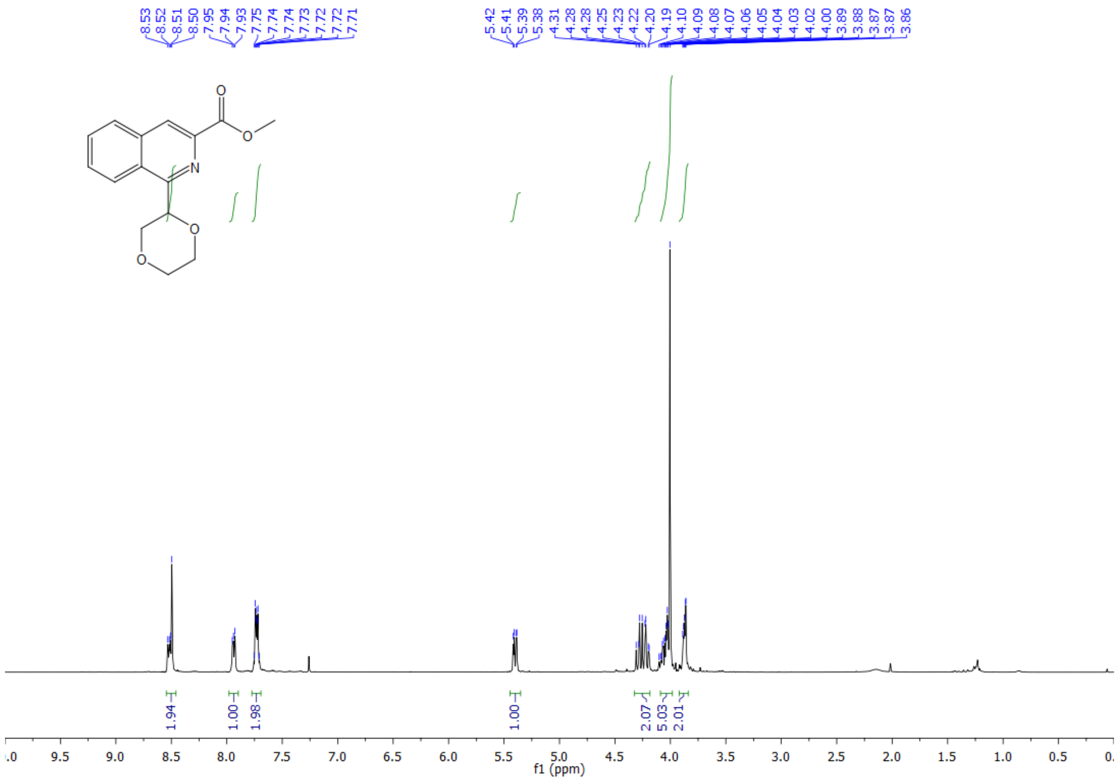

^{1}H NMR (400 MHz, $CDCl_3$) of **5i**

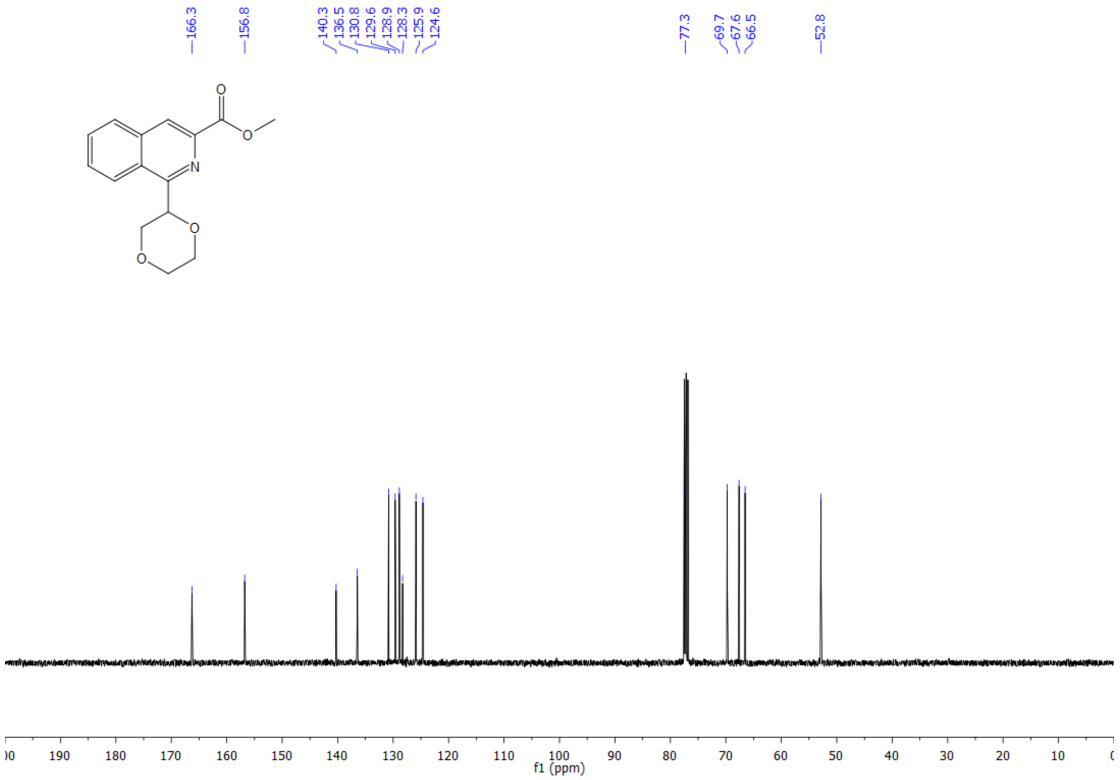

^{13}C NMR (101 MHz, $CDCl_3$) of **5i**

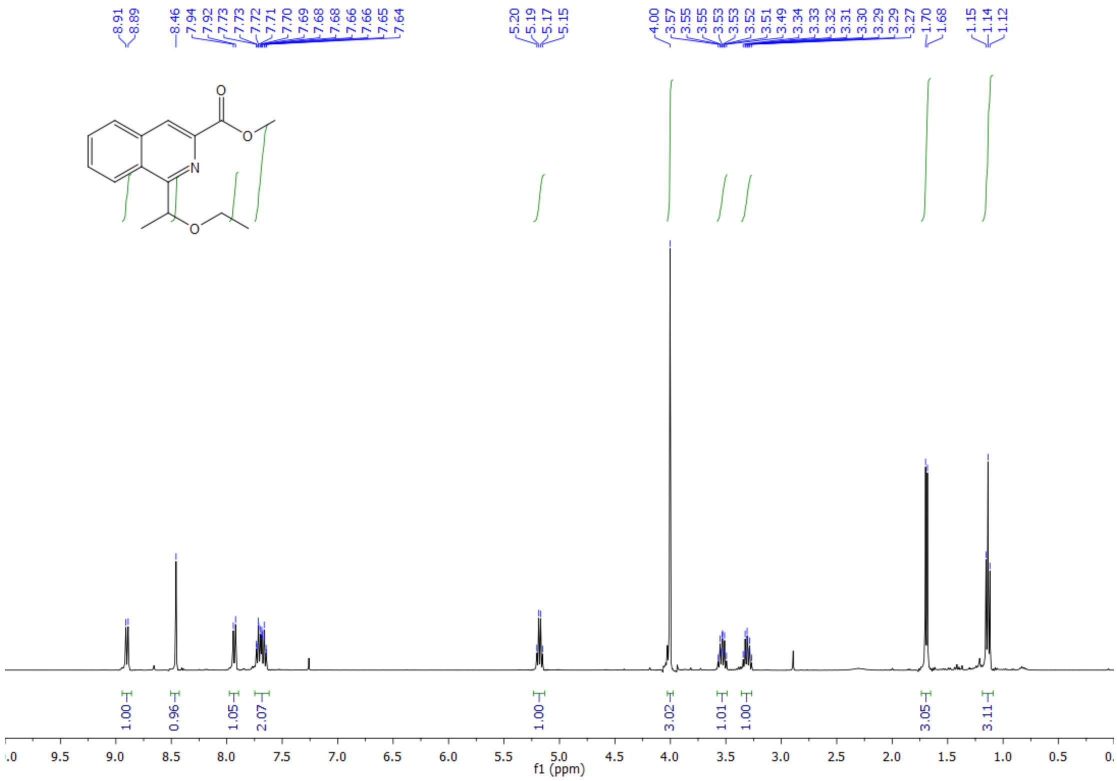

^{1}H NMR (400 MHz, $CDCl_3$) of **5j**

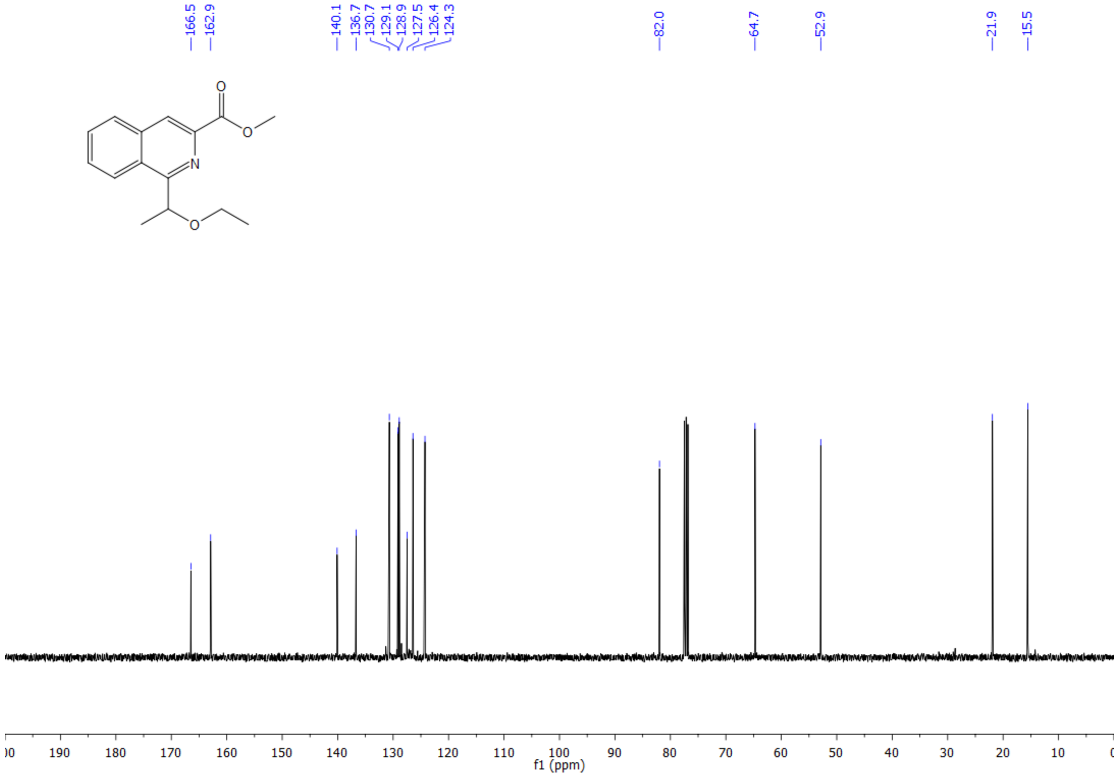

^{13}C NMR (101 MHz, $CDCl_3$) of **5j**

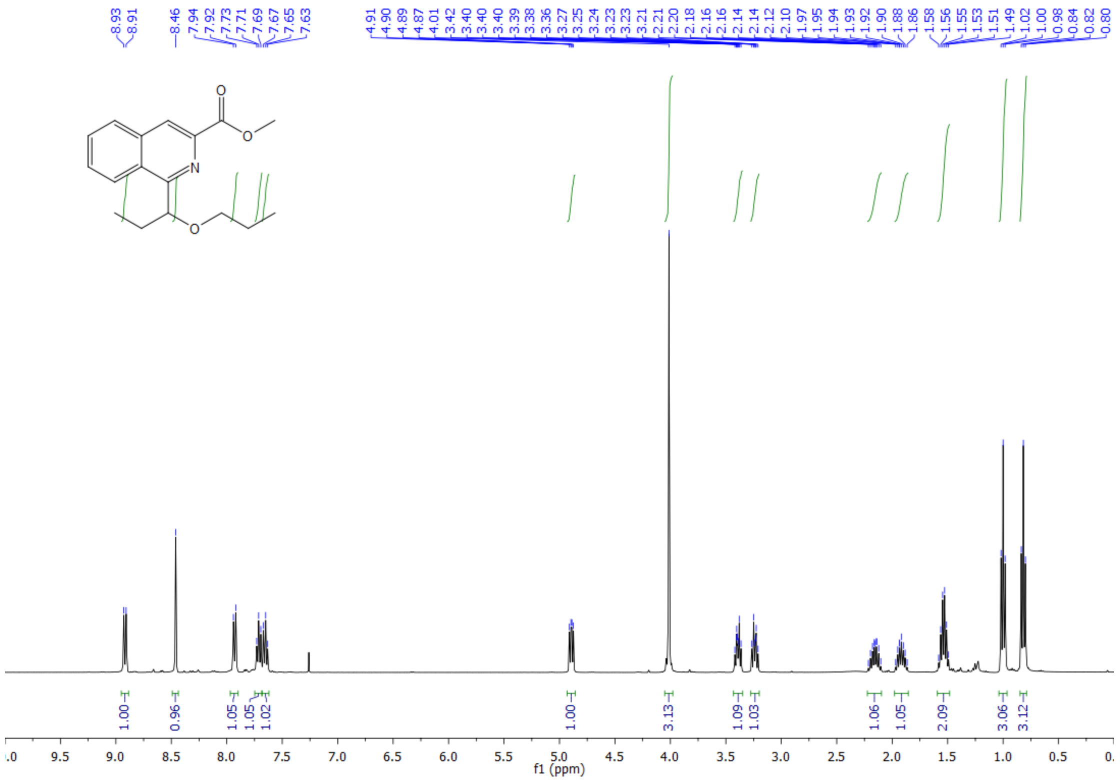

^{1}H NMR (400 MHz, $CDCl_3$) of **5k**

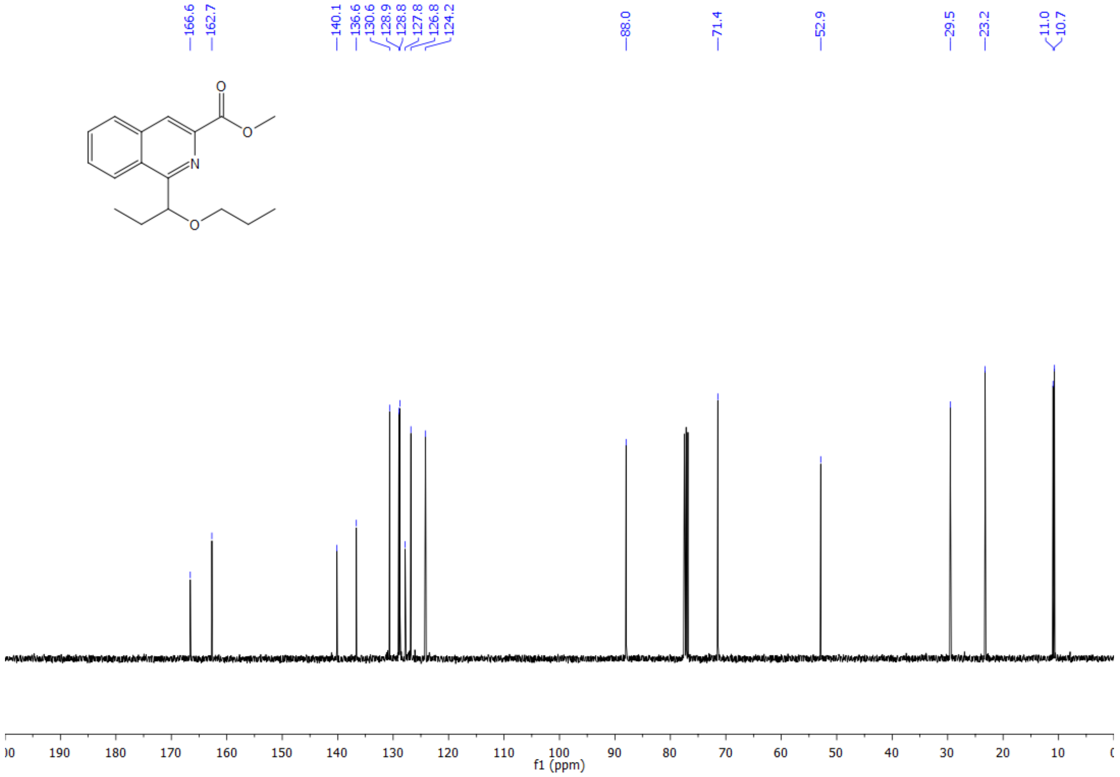

^{13}C NMR (101 MHz, $CDCl_3$) of **5k**

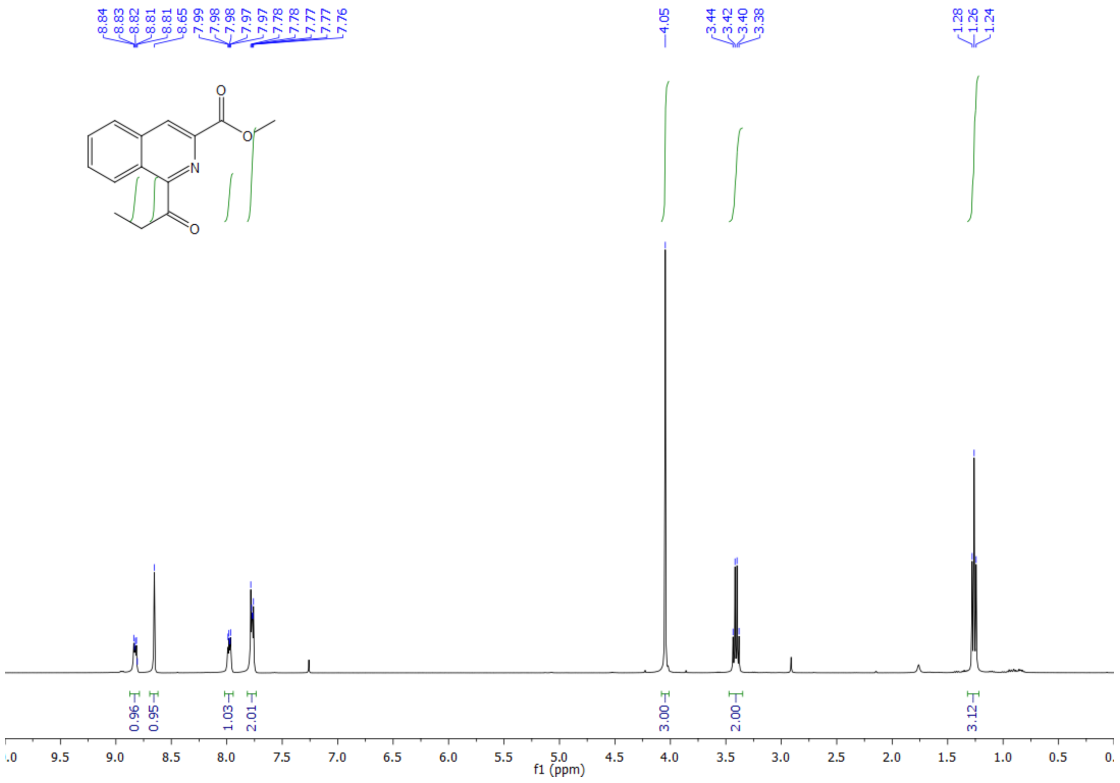

^{1}H NMR (400 MHz, $CDCl_3$) of **5l**

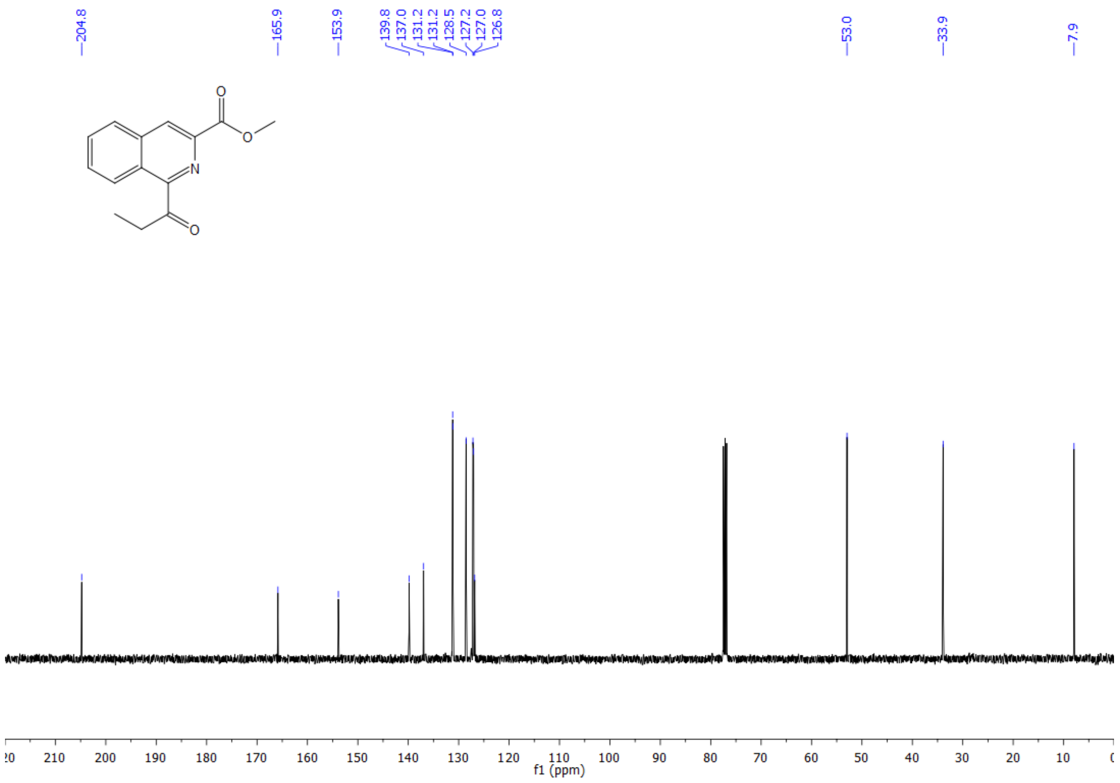

^{13}C NMR (101 MHz, $CDCl_3$) of **5l**

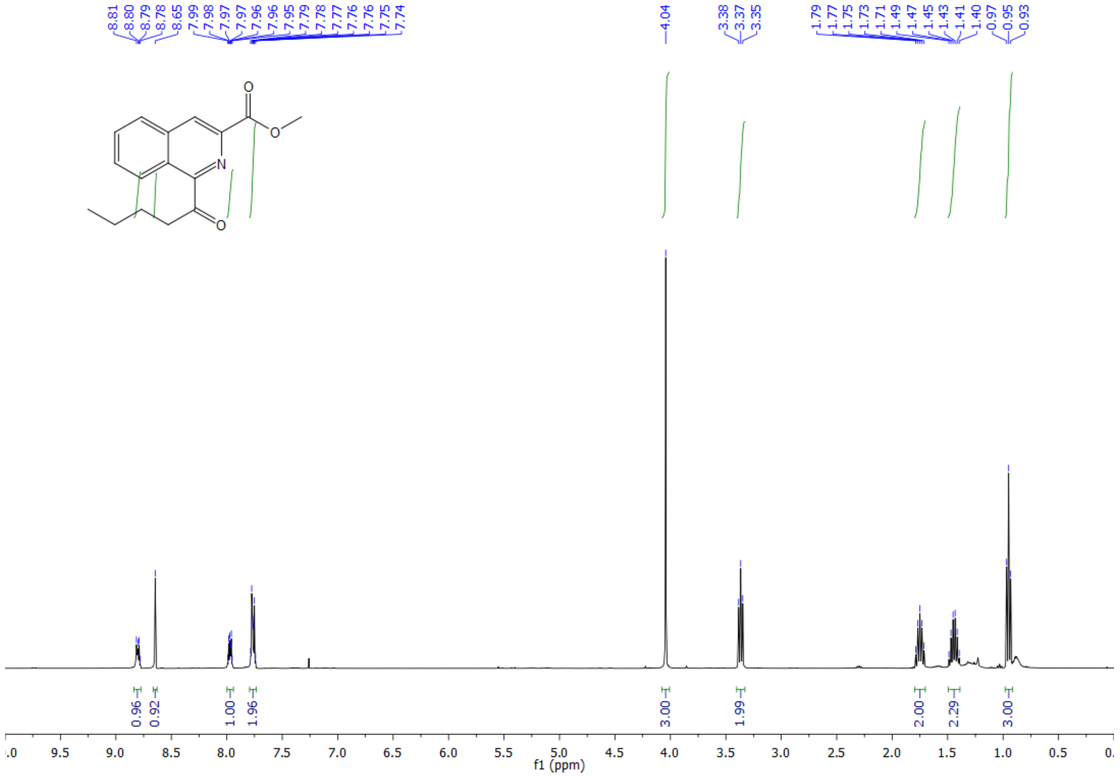

^{1}H NMR (400 MHz, $CDCl_3$) of **5m**

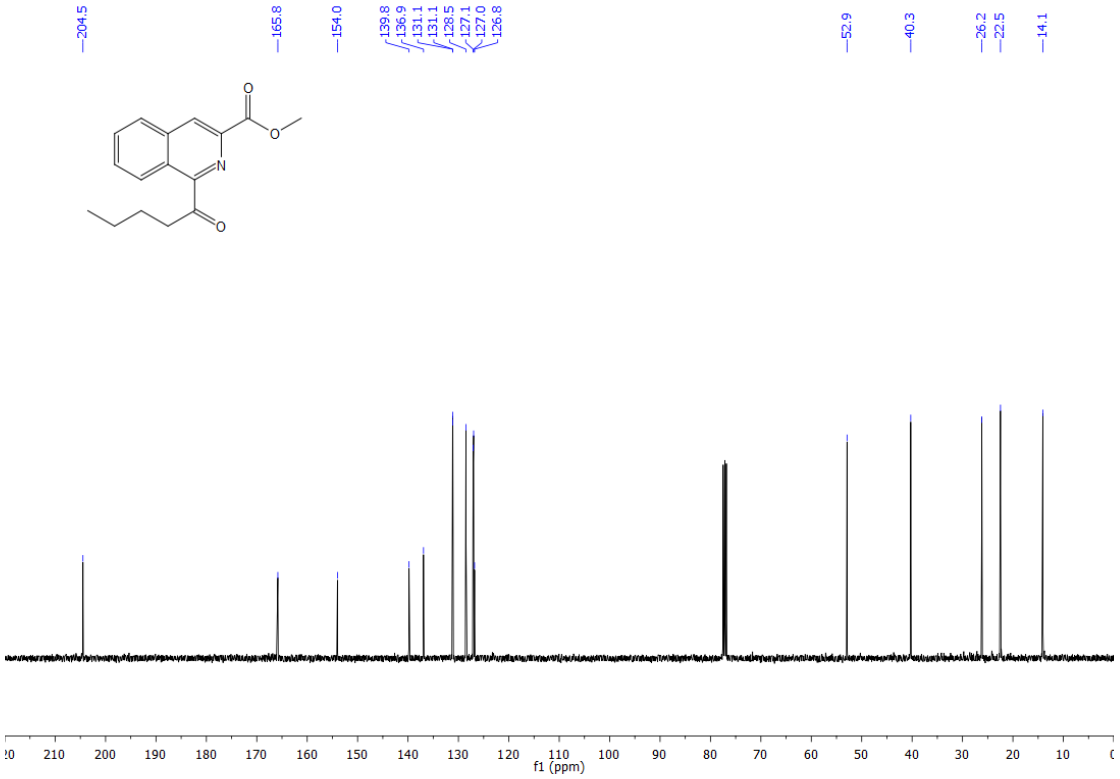

^{13}C NMR (101 MHz, $CDCl_3$) of **5m**

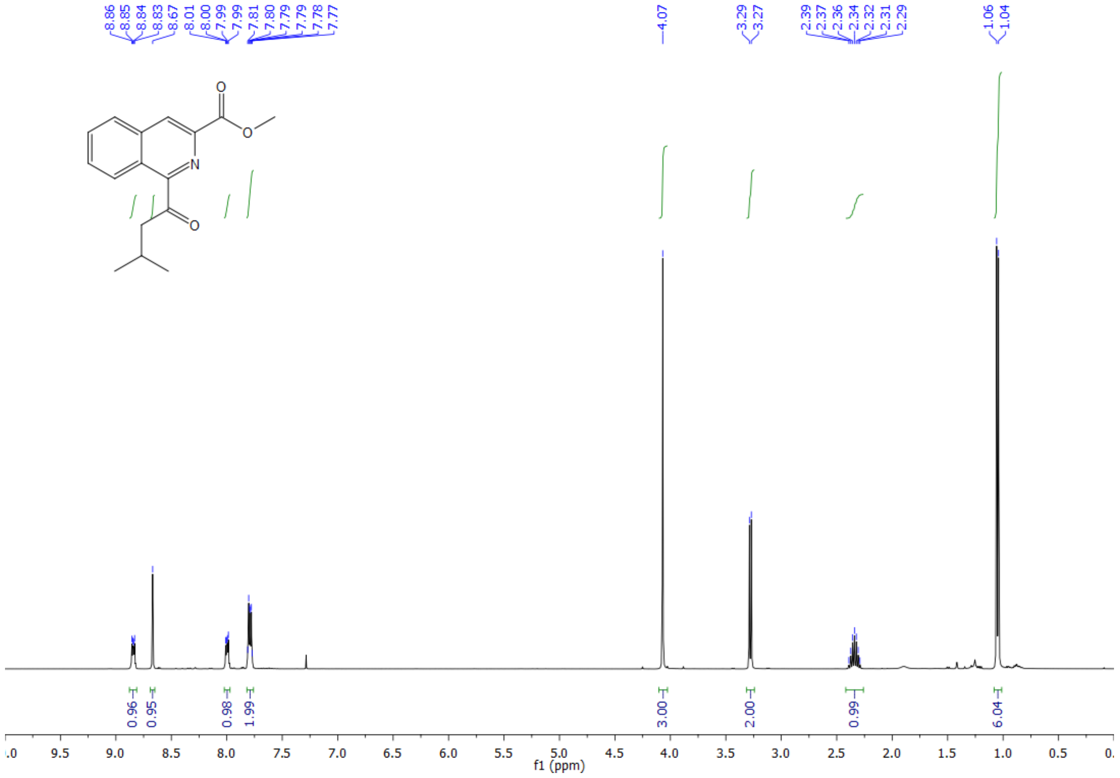

^{1}H NMR (400 MHz, $CDCl_3$) of **5n**

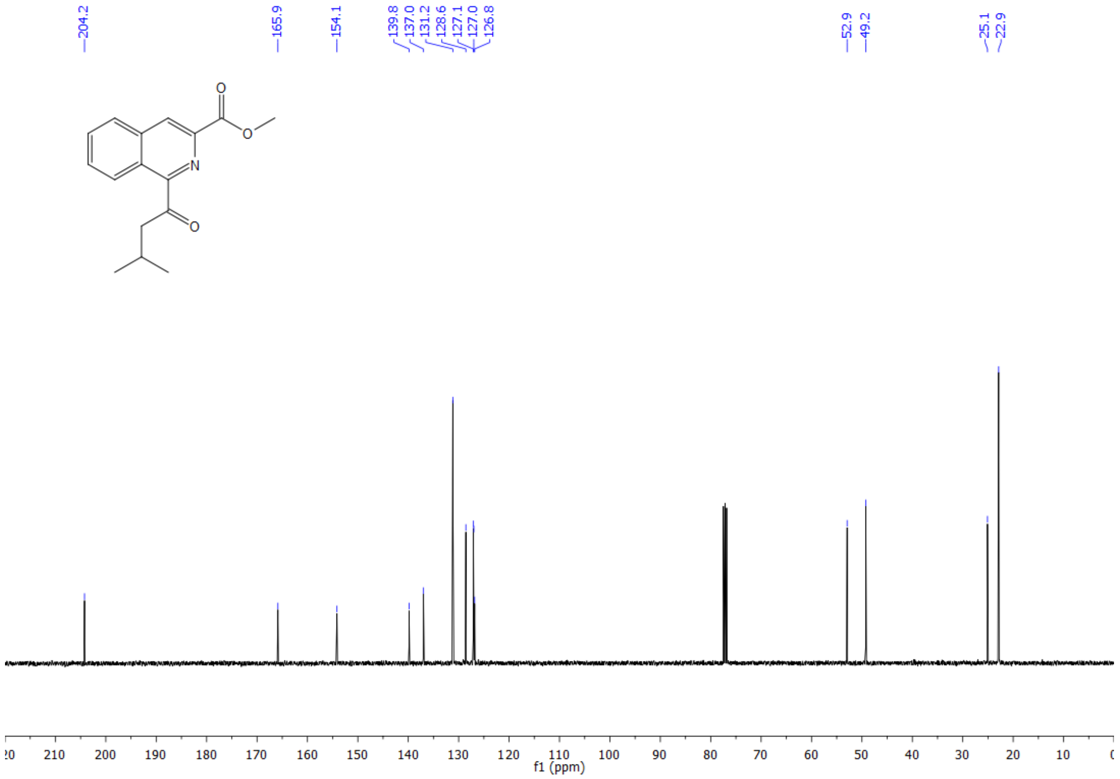

^{13}C NMR (101 MHz, $CDCl_3$) of **5n**

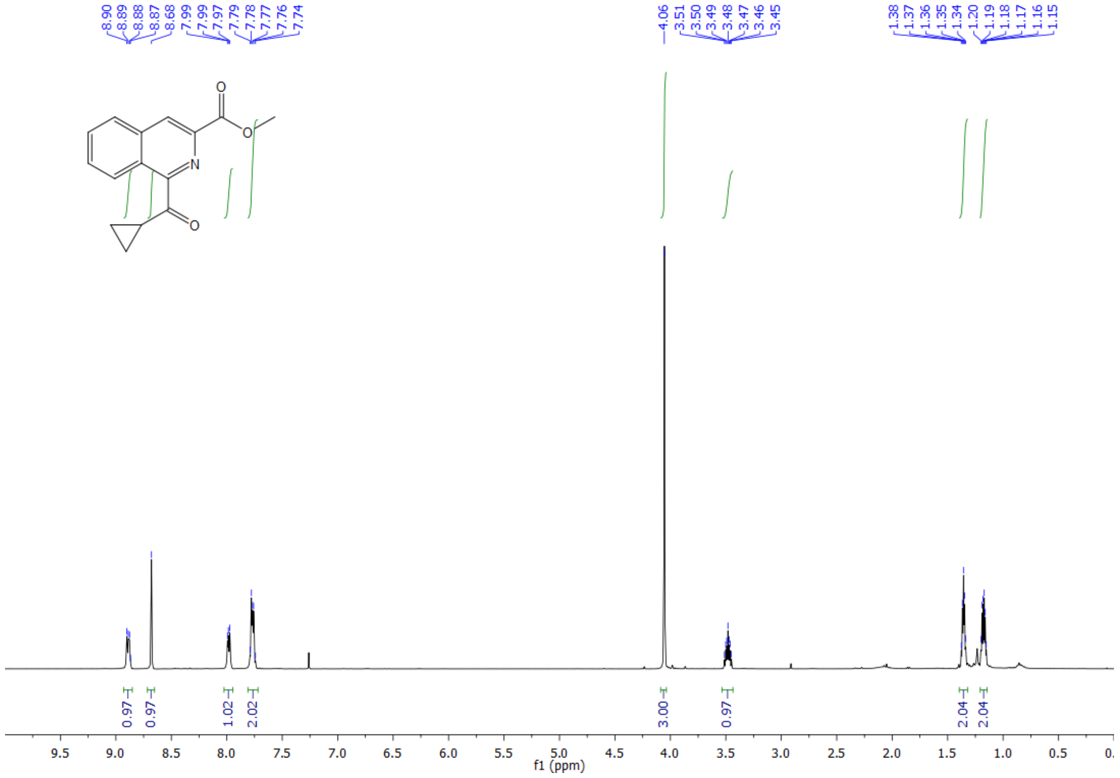

^{1}H NMR (400 MHz, $CDCl_3$) of **5o**

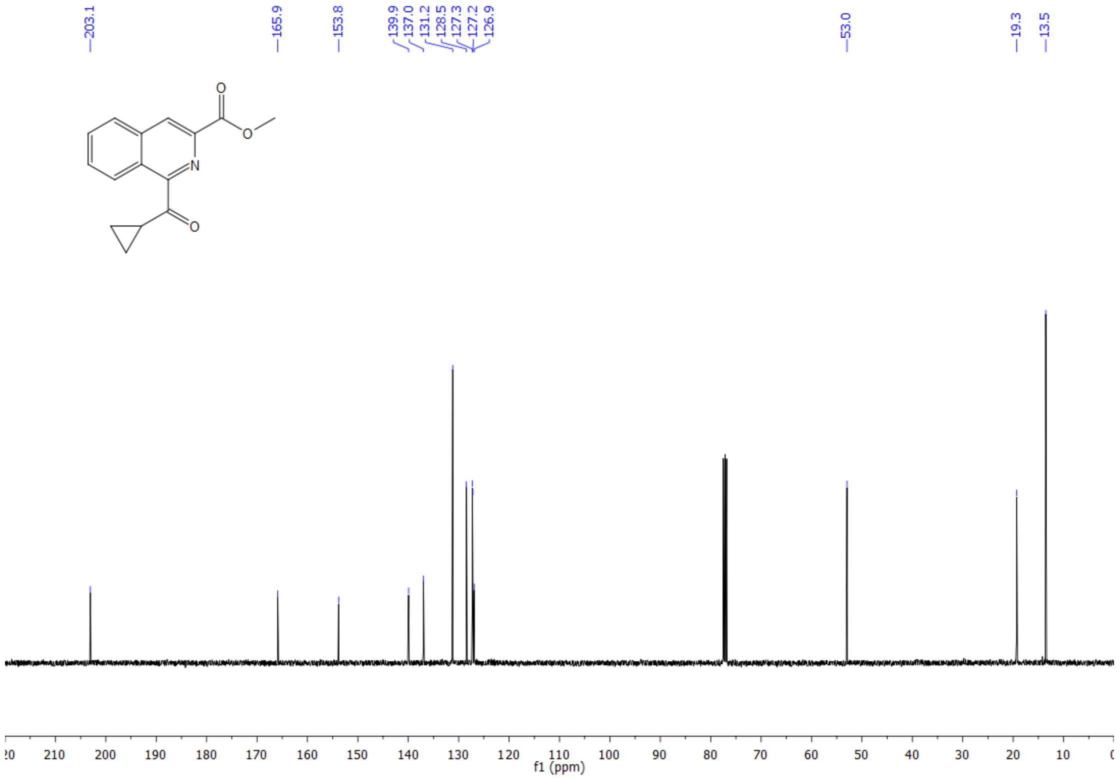

^{13}C NMR (101 MHz, $CDCl_3$) of **5o**

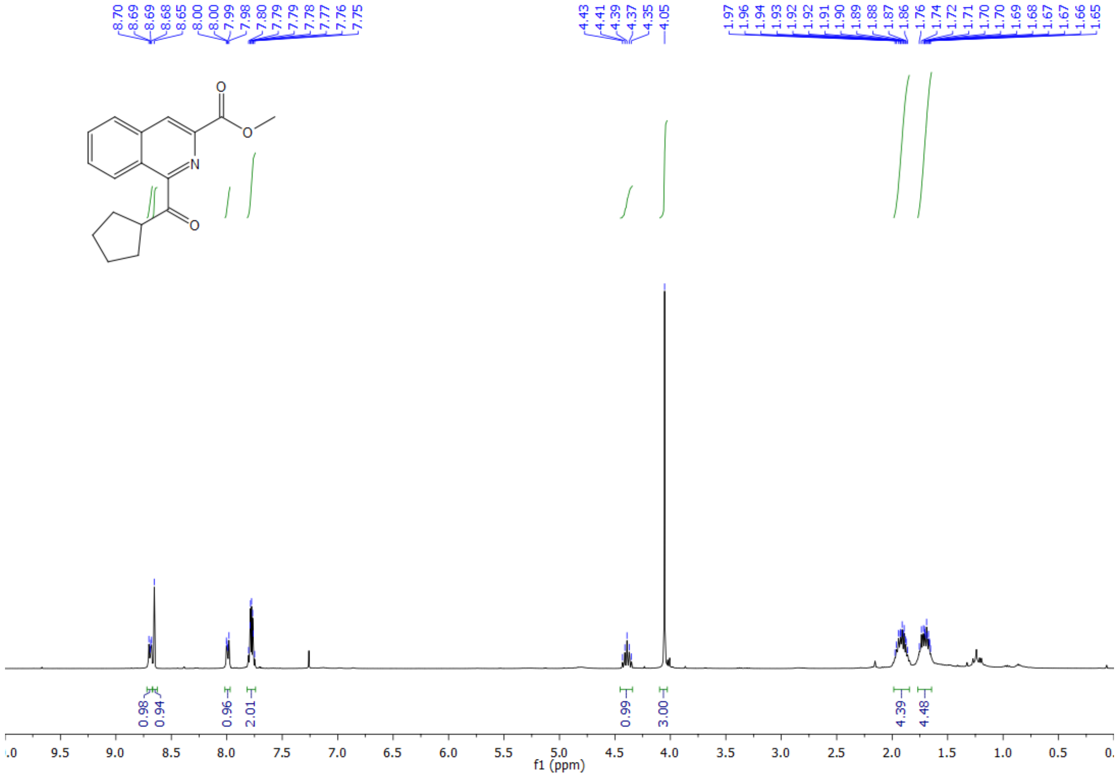

^{1}H NMR (400 MHz, $CDCl_3$) of **5p**

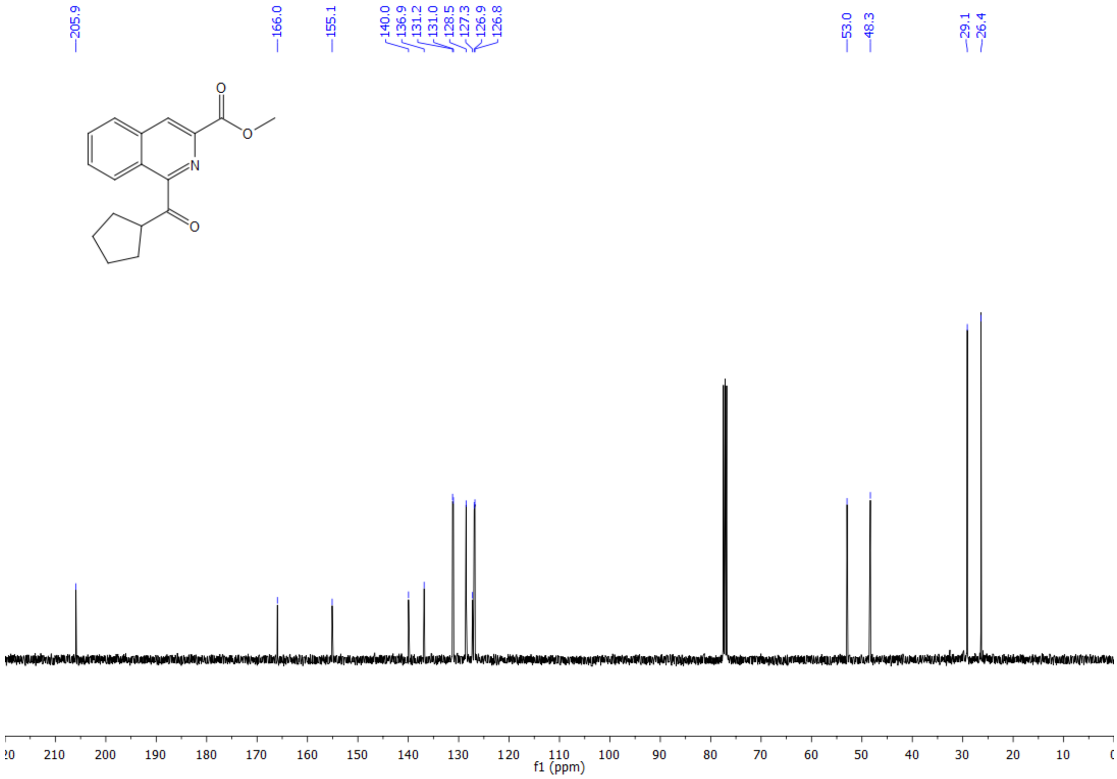

^{13}C NMR (101 MHz, $CDCl_3$) of **5p**

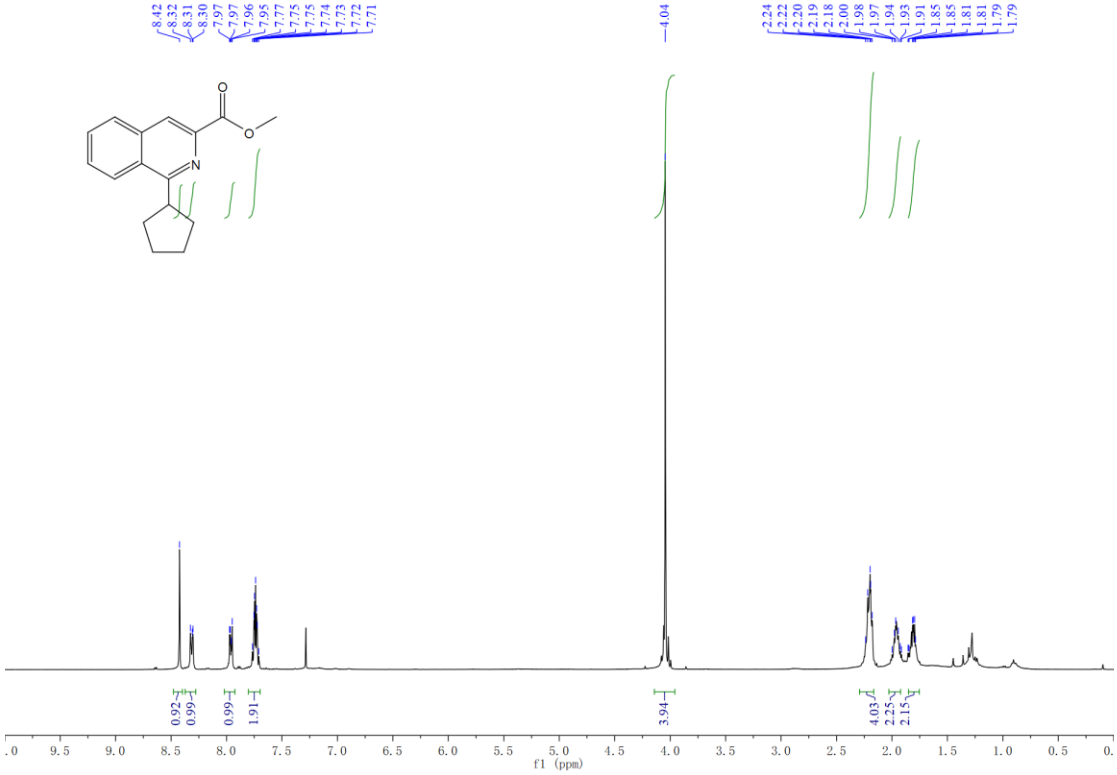

^{1}H NMR (400 MHz, $CDCl_3$) of **5p'**

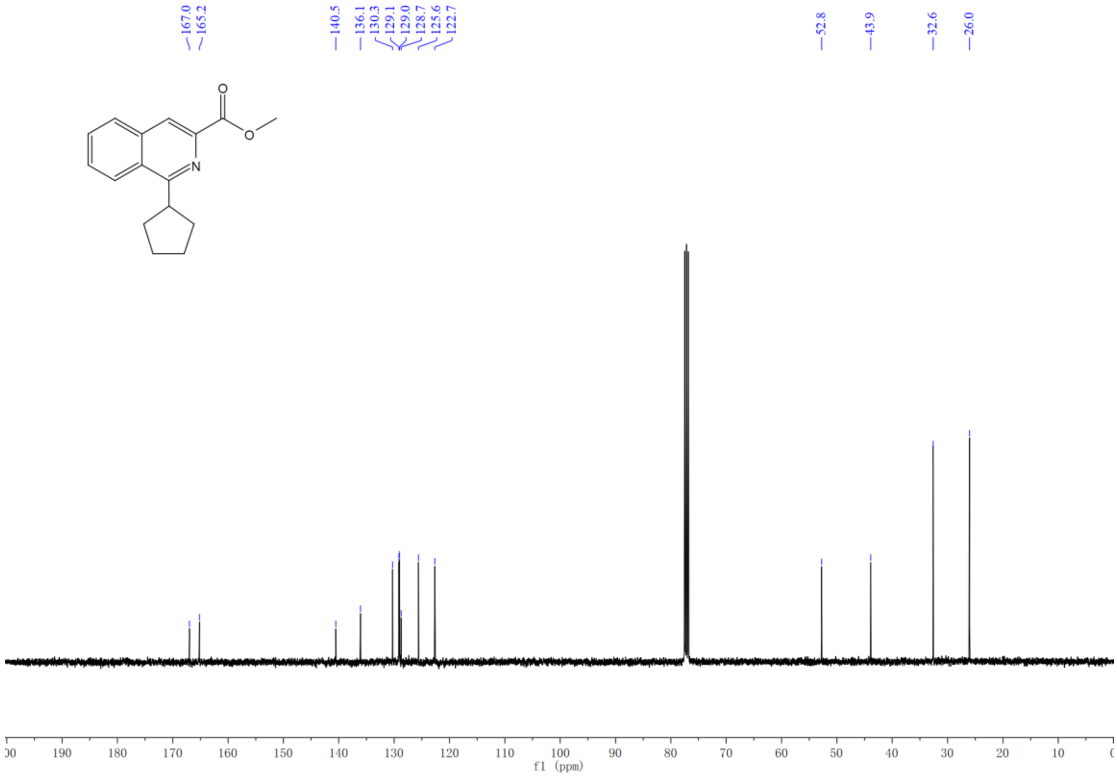

^{13}C NMR (101 MHz, $CDCl_3$) of **5p'**

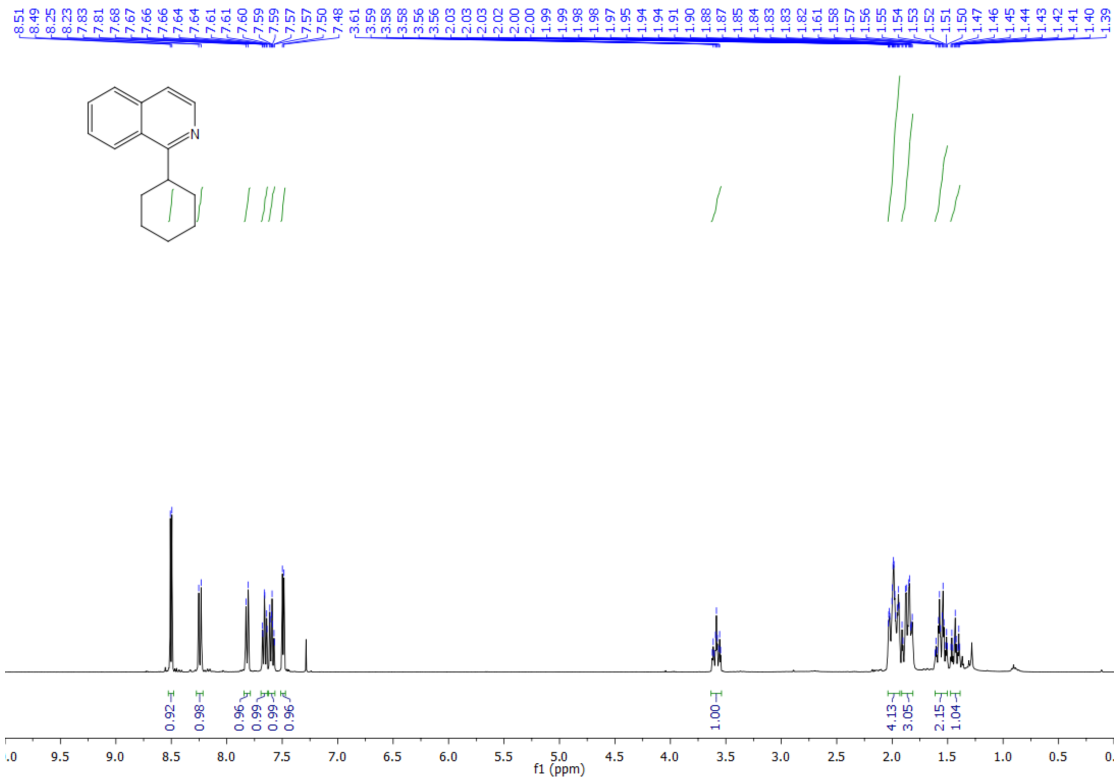

^{1}H NMR (400 MHz, $CDCl_3$) of **6a**

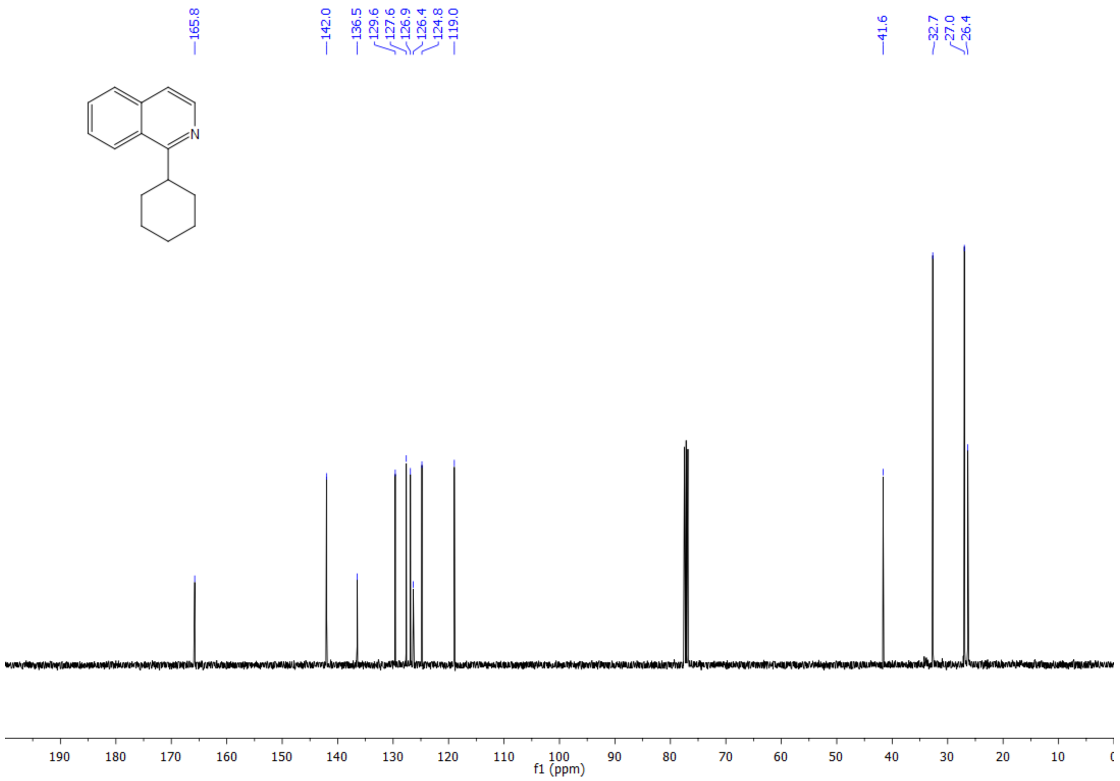

^{13}C NMR (101 MHz, $CDCl_3$) of **6a**

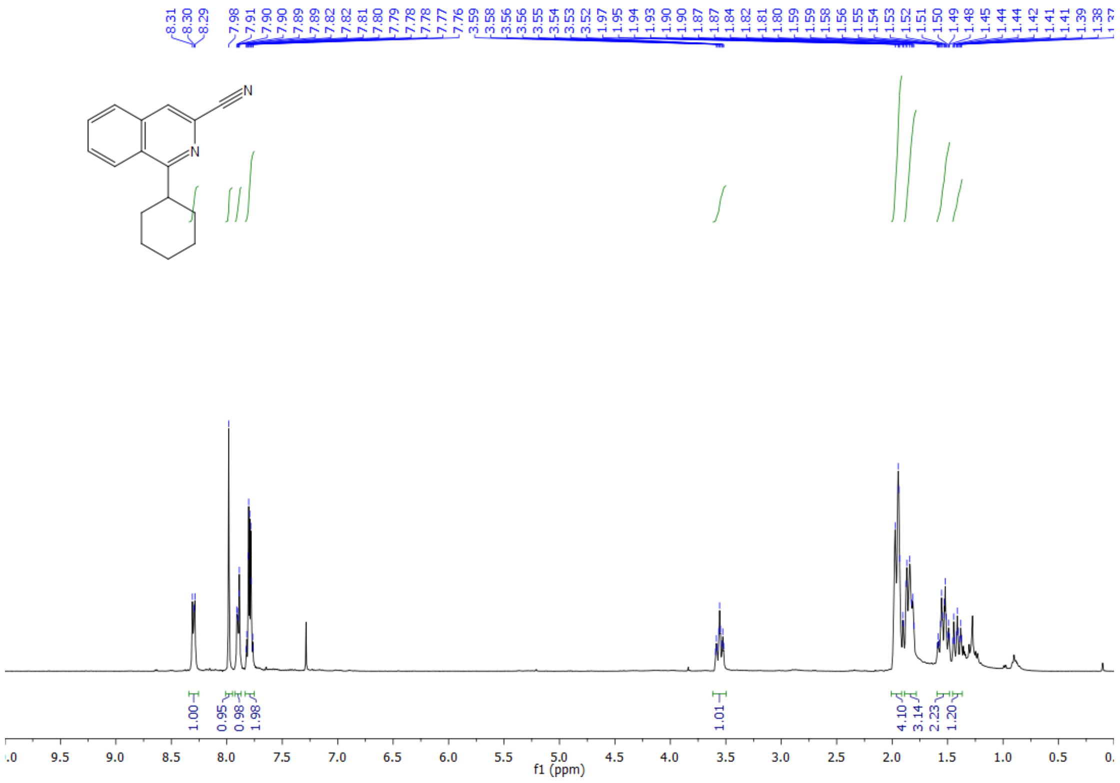

^{1}H NMR (400 MHz, $CDCl_3$) of **6b**

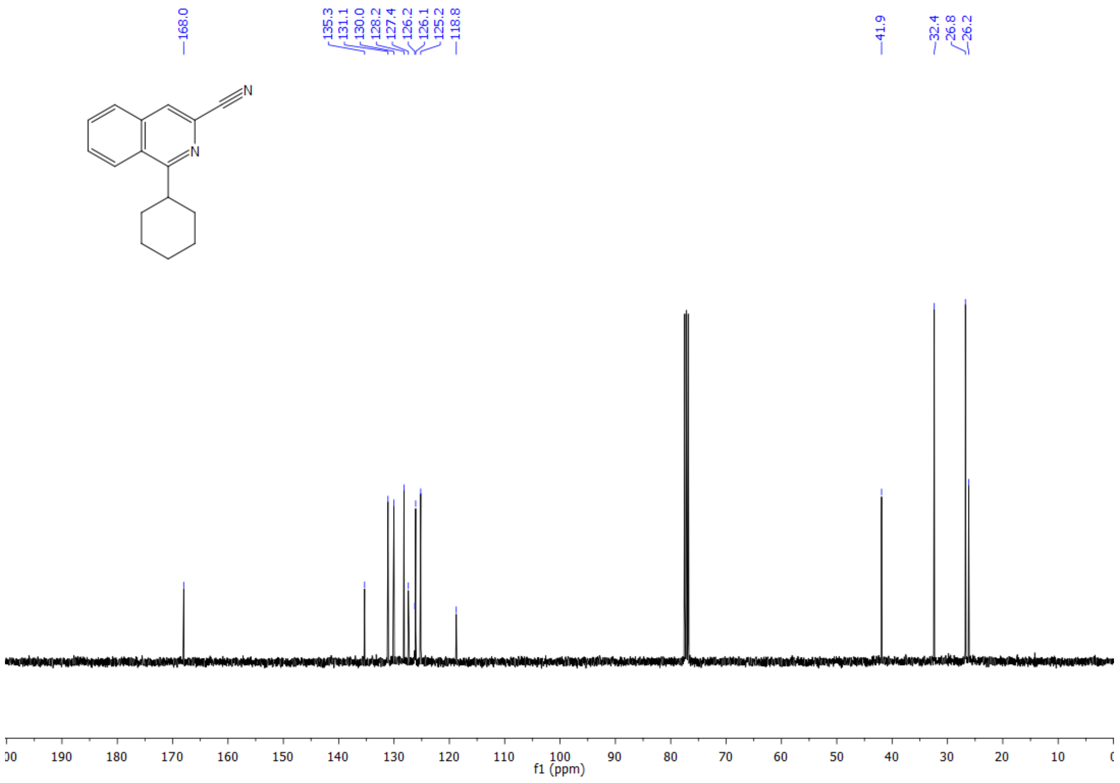

^{13}C NMR (101 MHz, $CDCl_3$) of **6b**

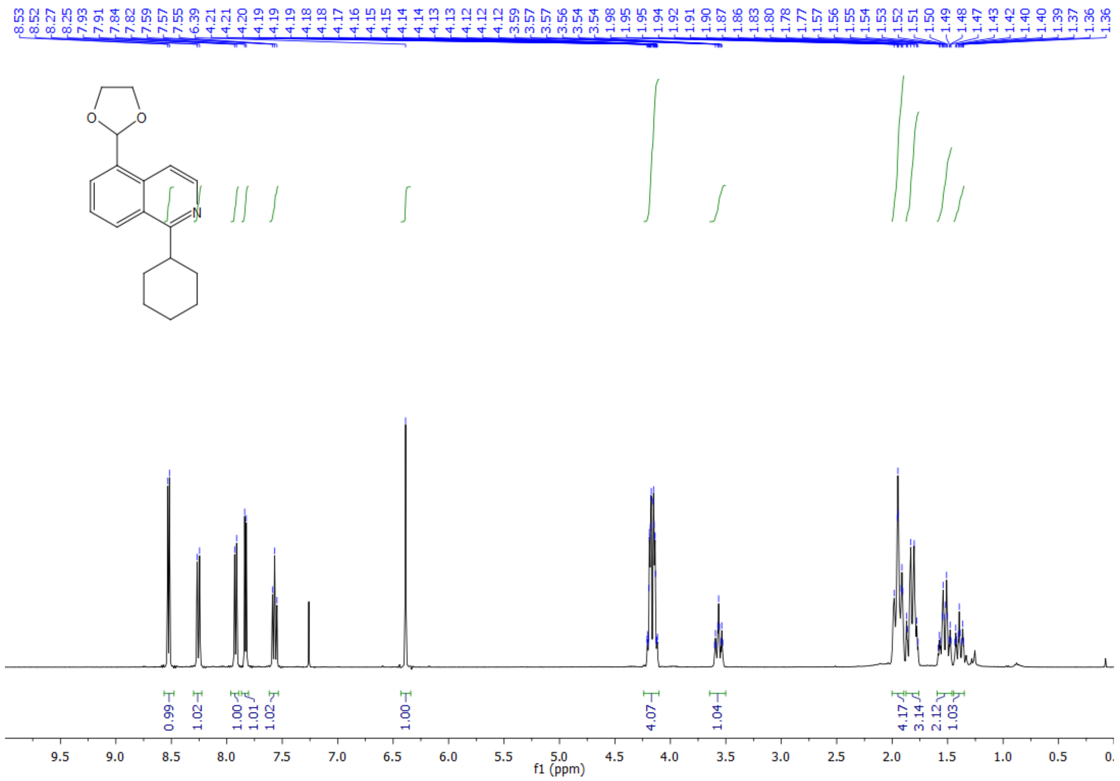

^{1}H NMR (400 MHz, $CDCl_3$) of **6c**

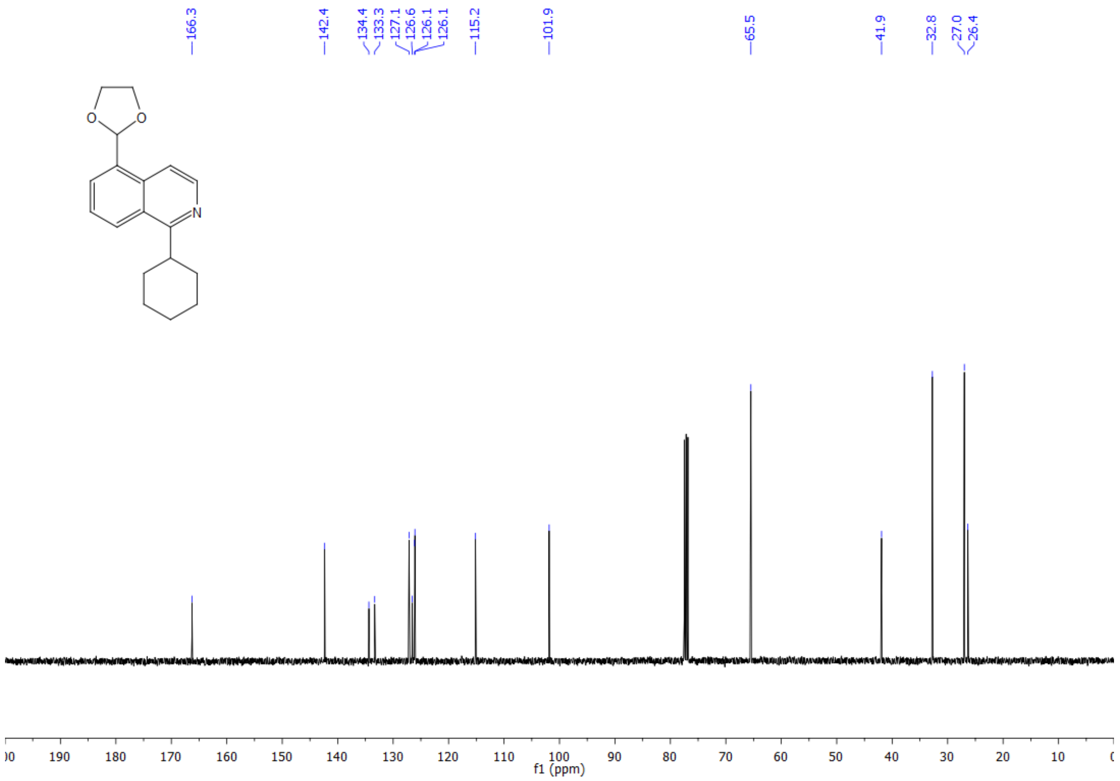

^{13}C NMR (101 MHz, $CDCl_3$) of **6c**

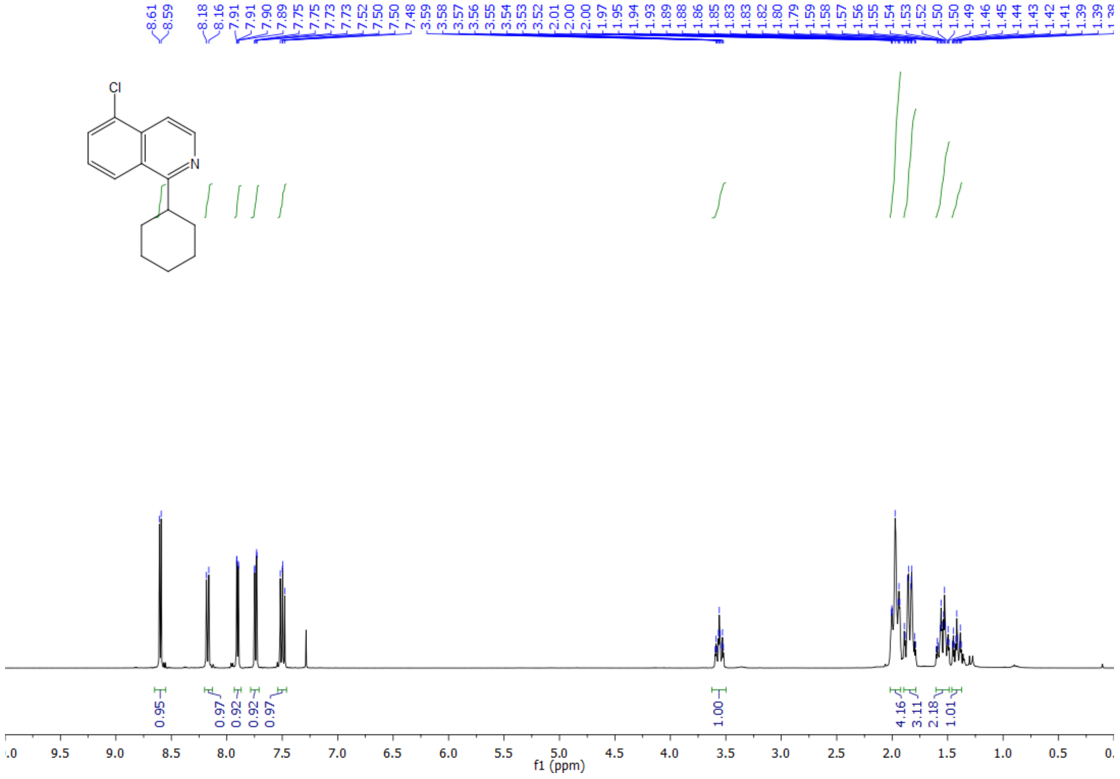

^{1}H NMR (400 MHz, $CDCl_3$) of **6d**

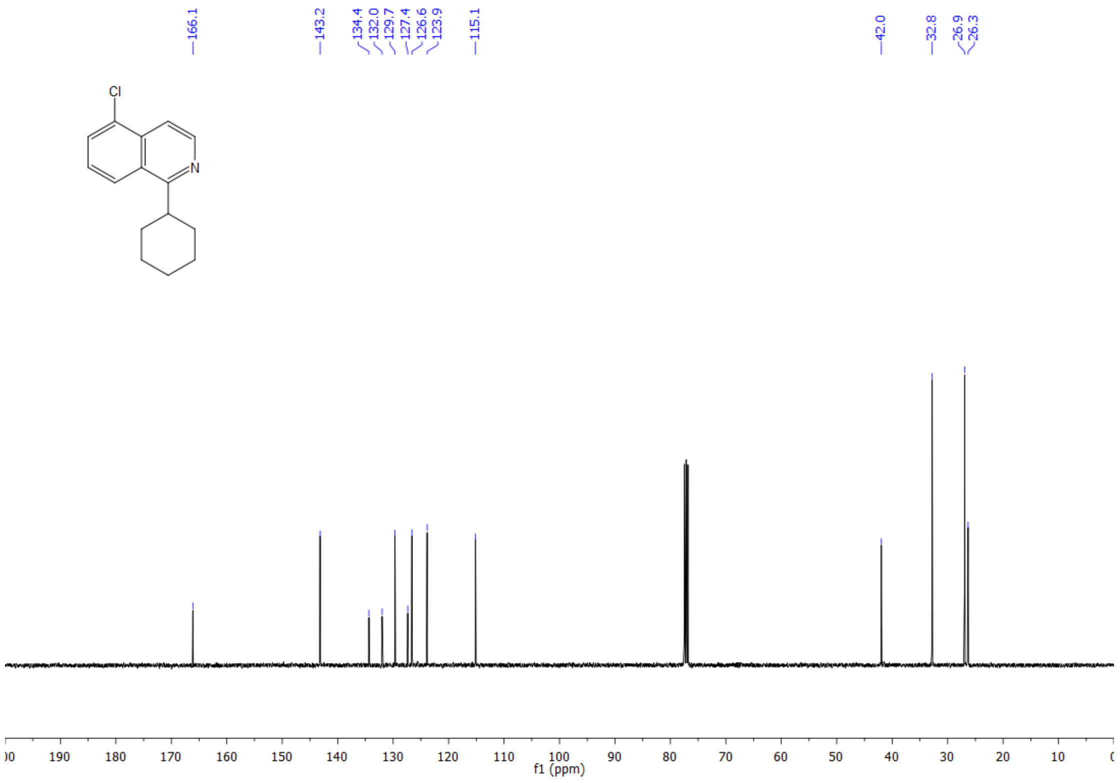

^{13}C NMR (101 MHz, $CDCl_3$) of **6d**

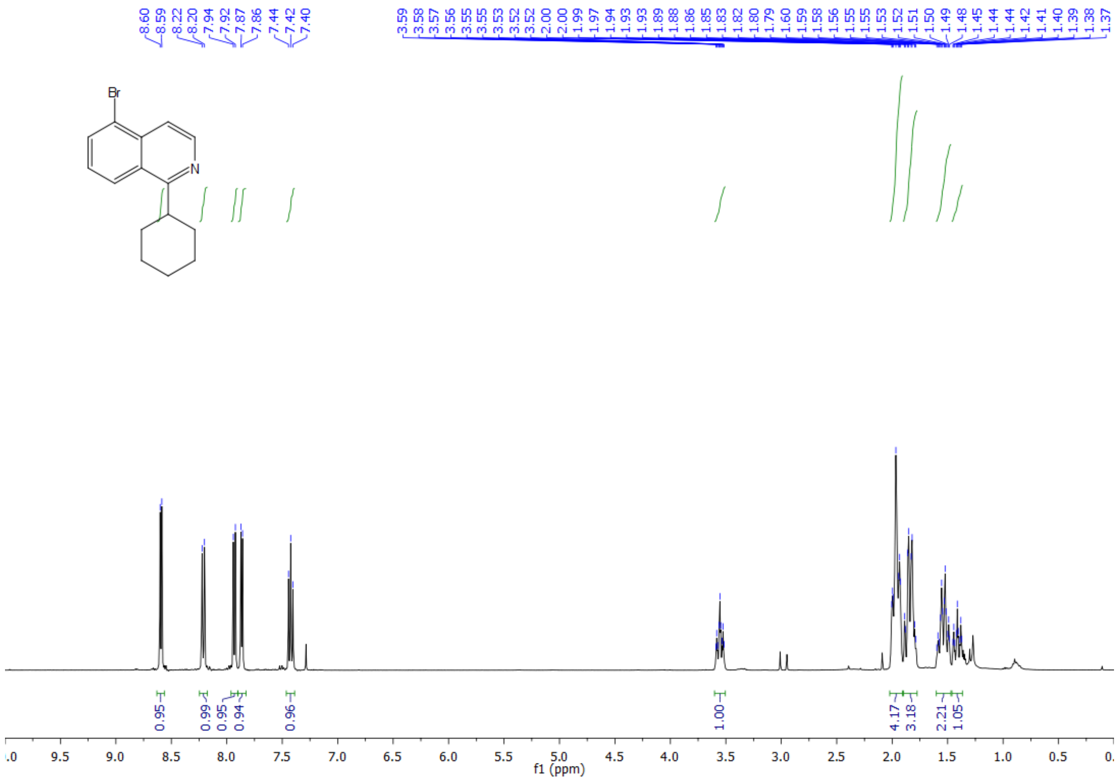

^{1}H NMR (400 MHz, $CDCl_3$) of **6e**

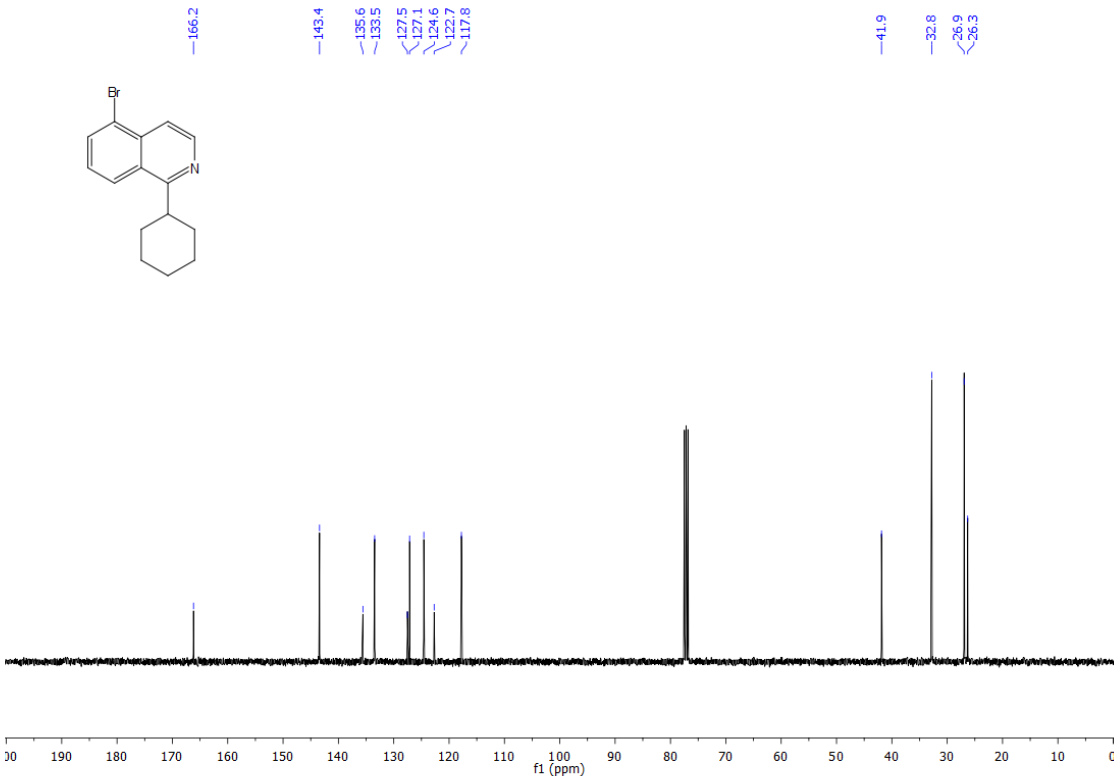

^{13}C NMR (101 MHz, $CDCl_3$) of **6e**

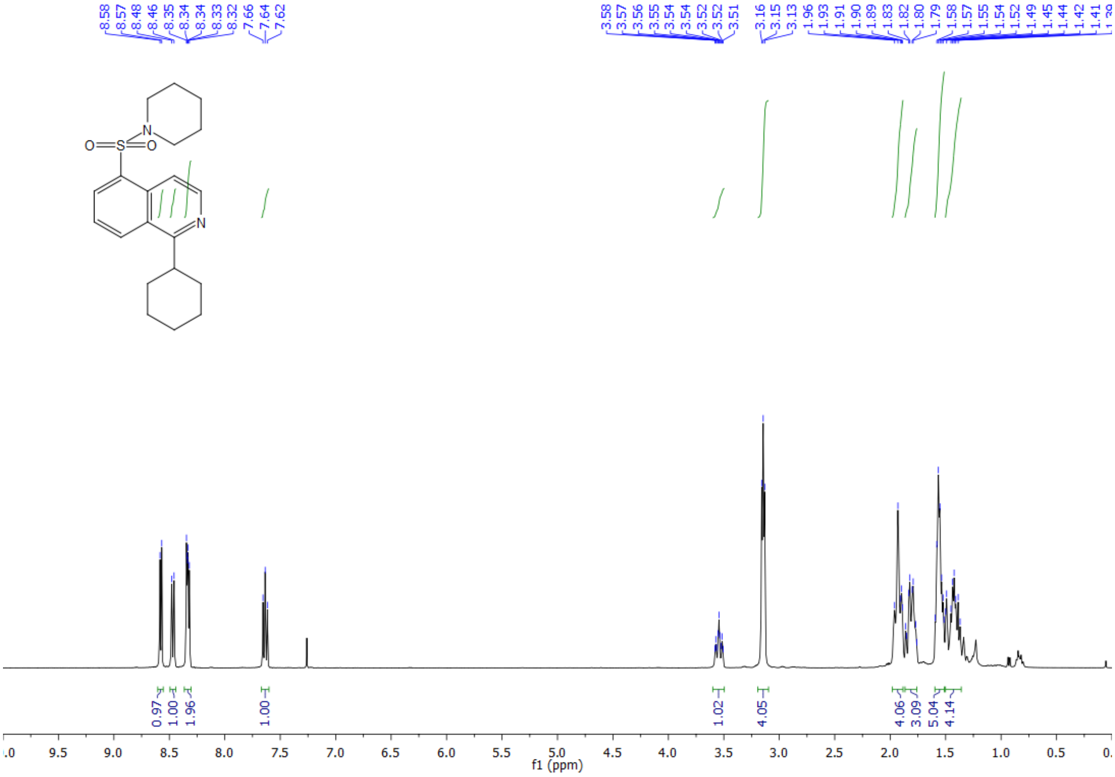

^{1}H NMR (400 MHz, $CDCl_3$) of **6f**

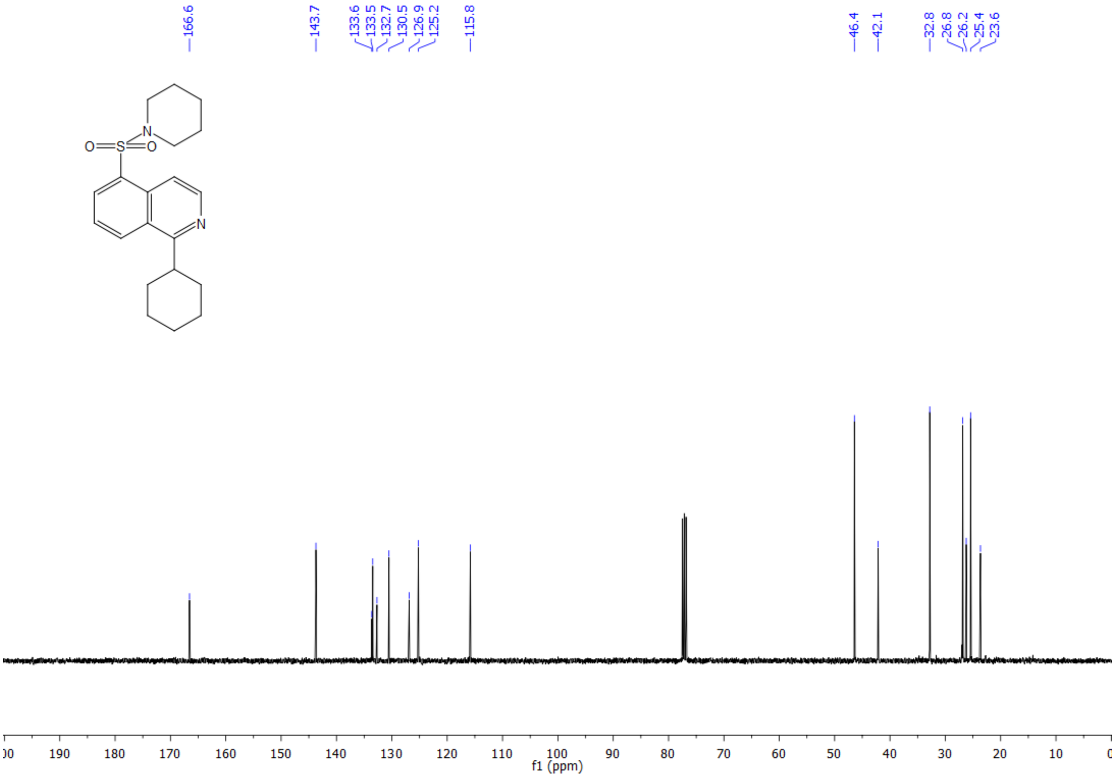

^{13}C NMR (101 MHz, $CDCl_3$) of **6f**

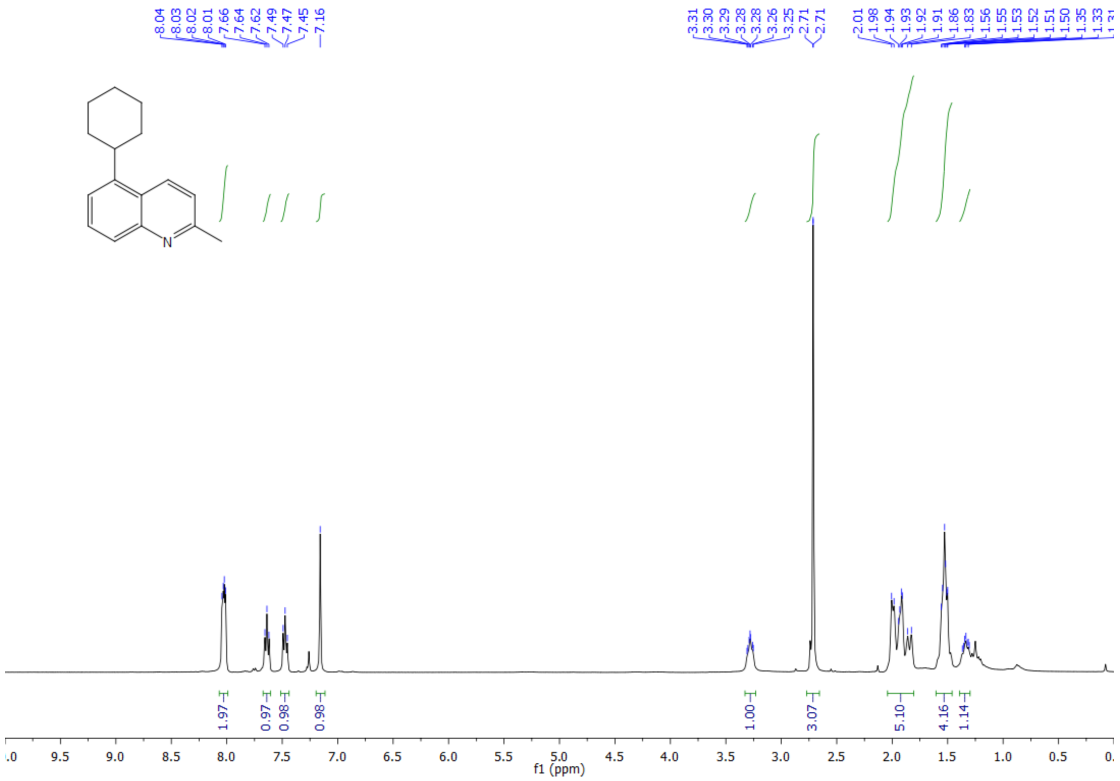

^{1}H NMR (400 MHz, $CDCl_3$) of **6g**

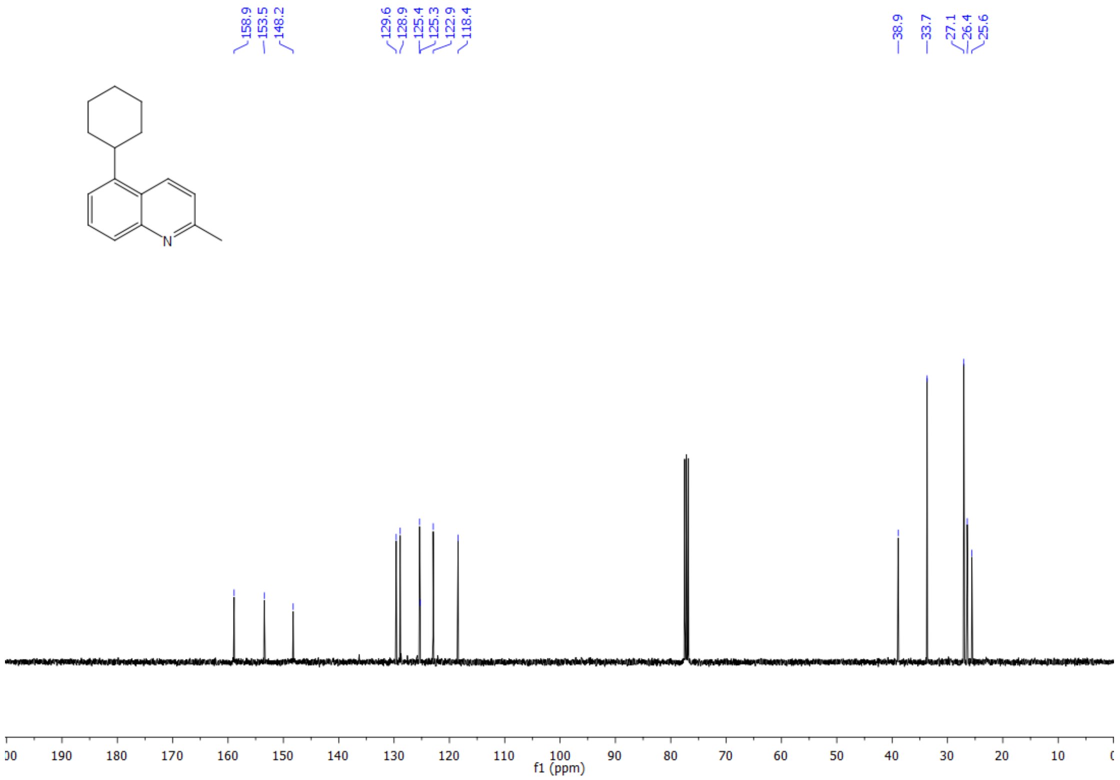

^{13}C NMR (101 MHz, $CDCl_3$) of **6g**

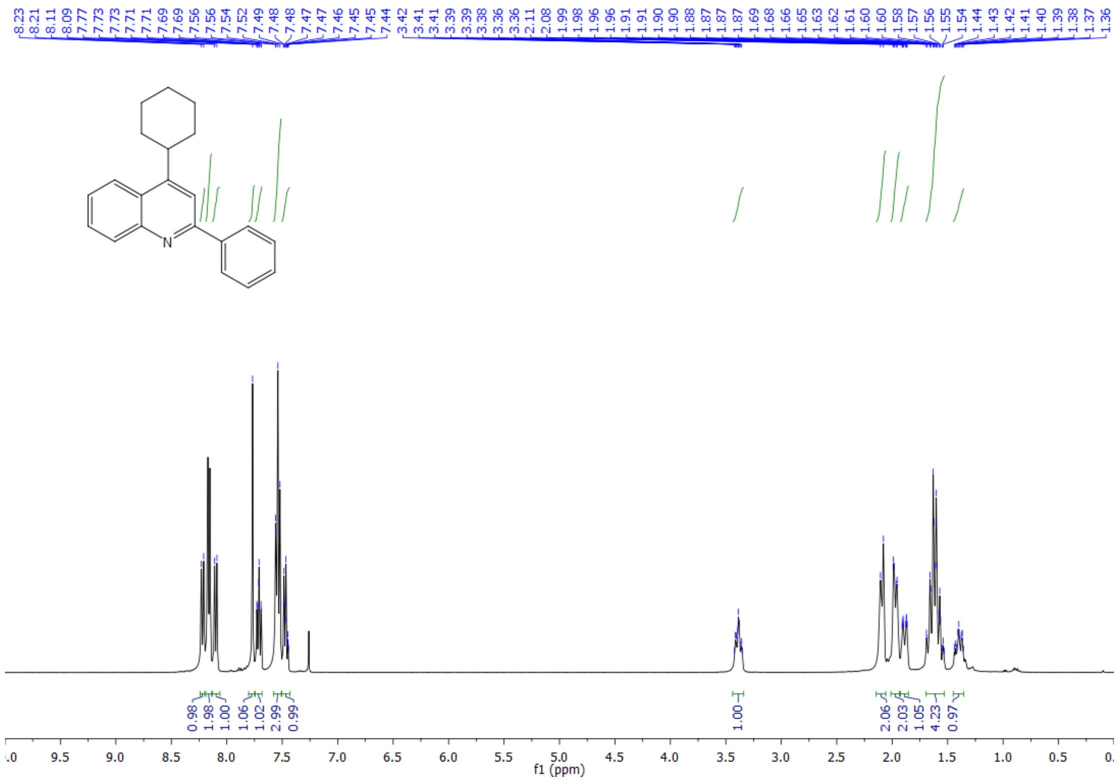

^{1}H NMR (400 MHz, $CDCl_3$) of **6h**

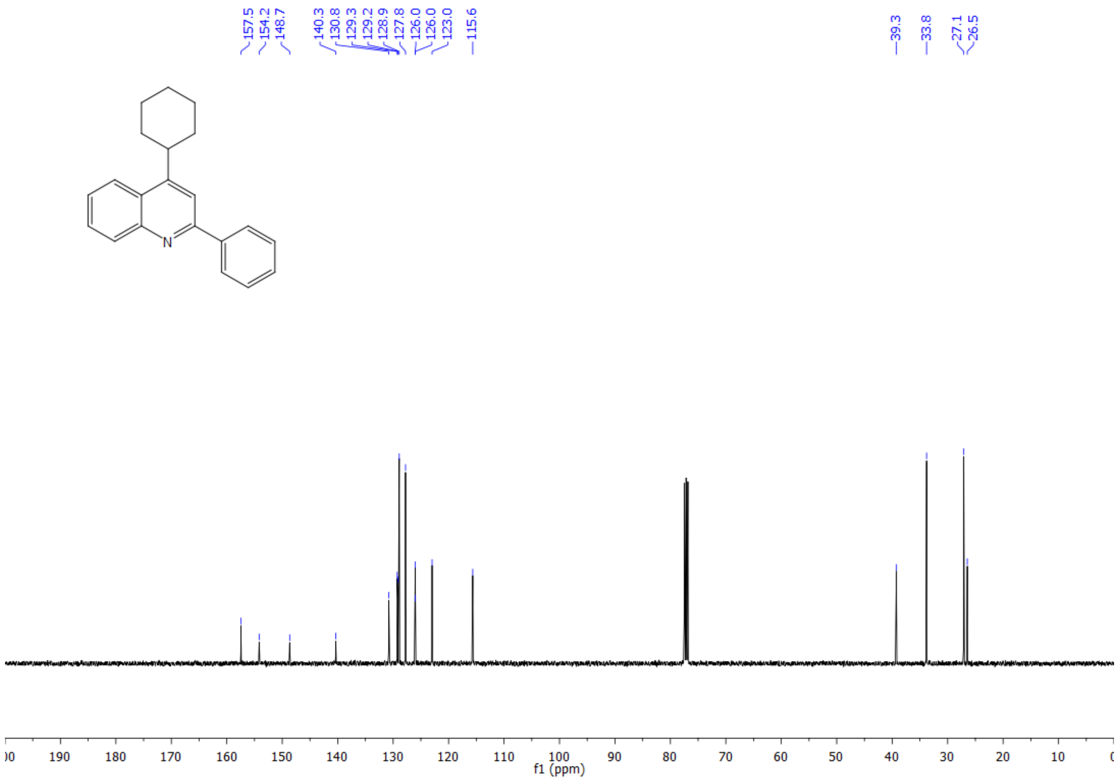

^{13}C NMR (101 MHz, $CDCl_3$) of **6h**

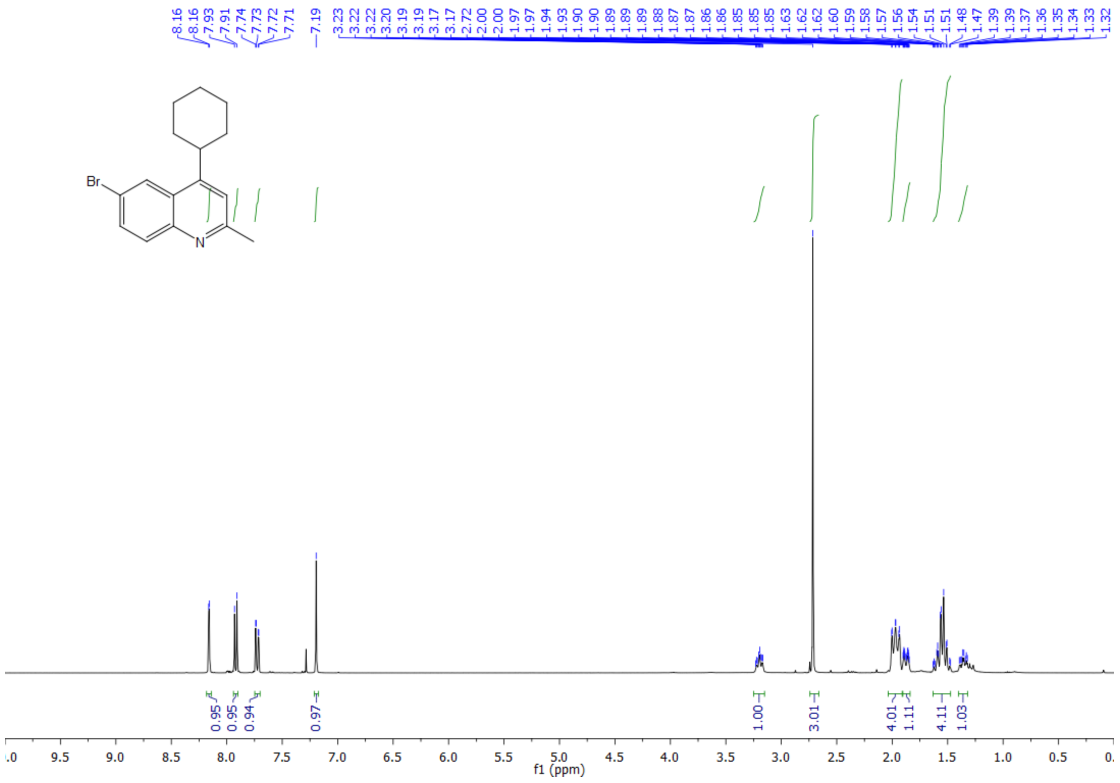

^{1}H NMR (400 MHz, $CDCl_3$) of **6i**

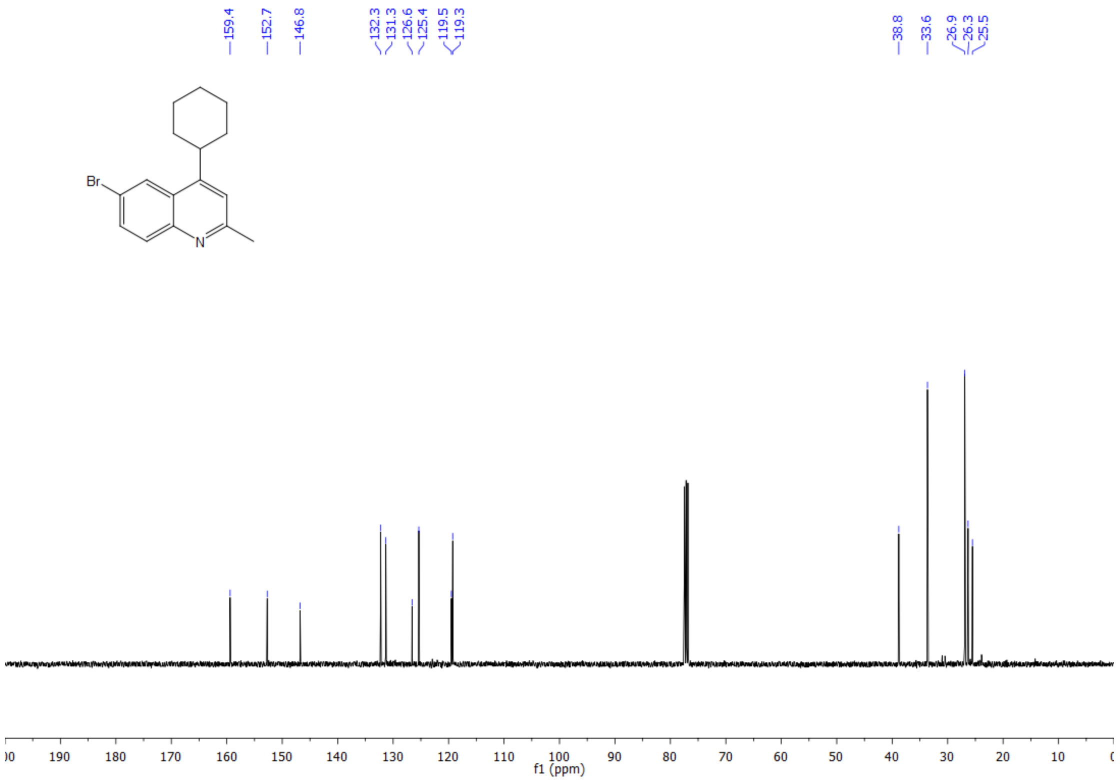

^{13}C NMR (101 MHz, $CDCl_3$) of **6i**

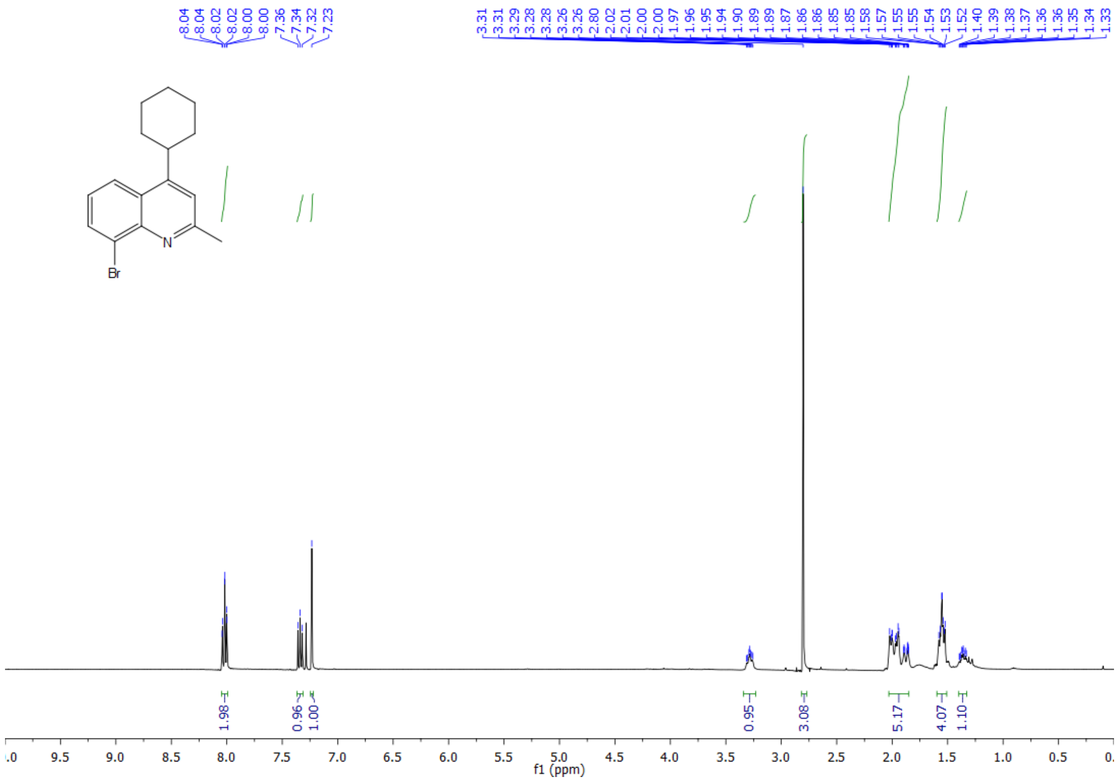

^{1}H NMR (400 MHz, $CDCl_3$) of **6j**

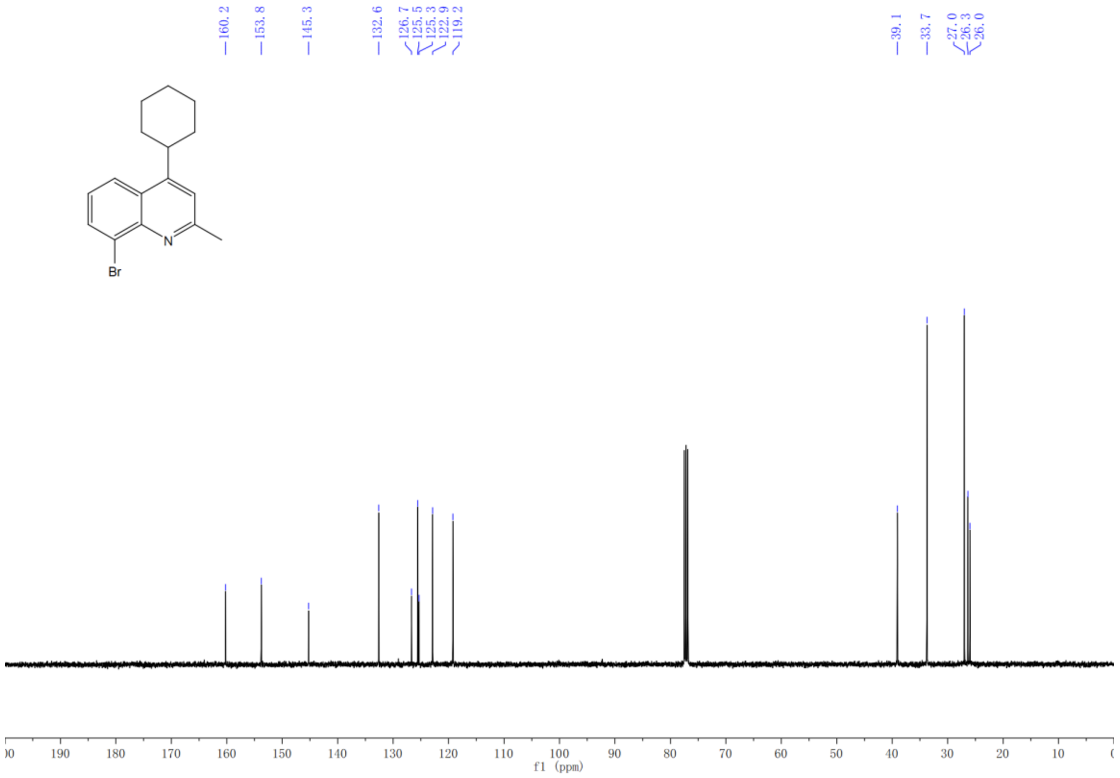

^{13}C NMR (101 MHz, $CDCl_3$) of **6j**

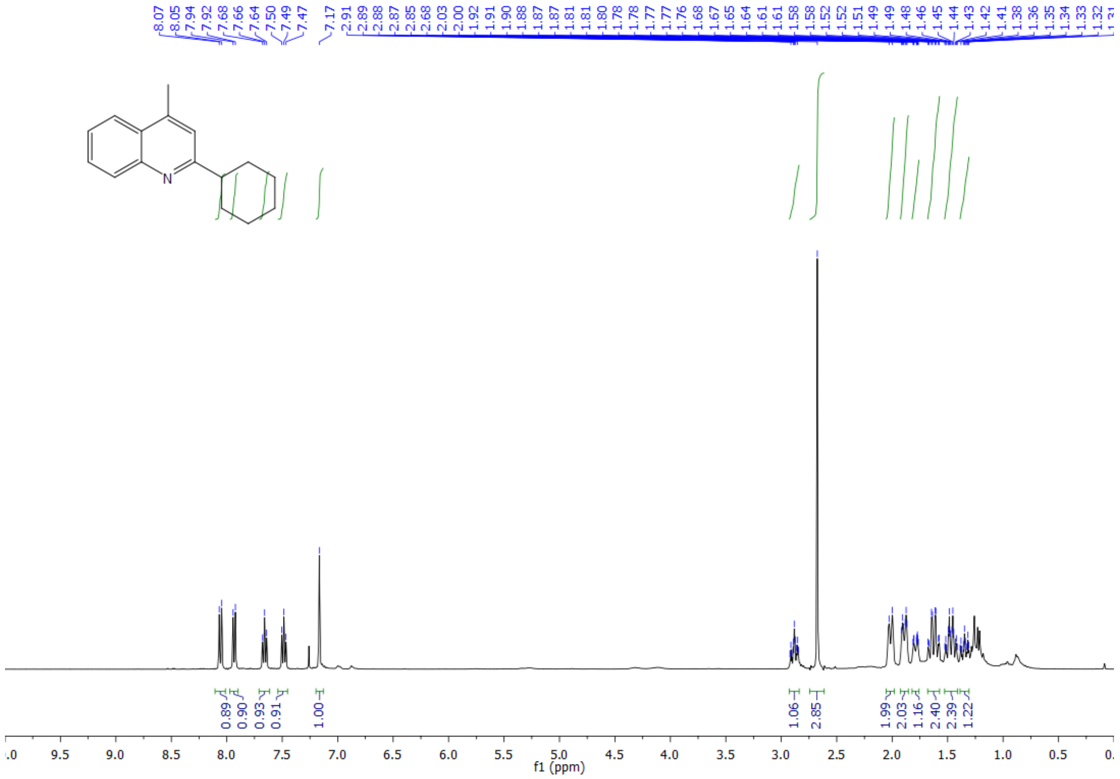

^{1}H NMR (400 MHz, $CDCl_3$) of **6k**

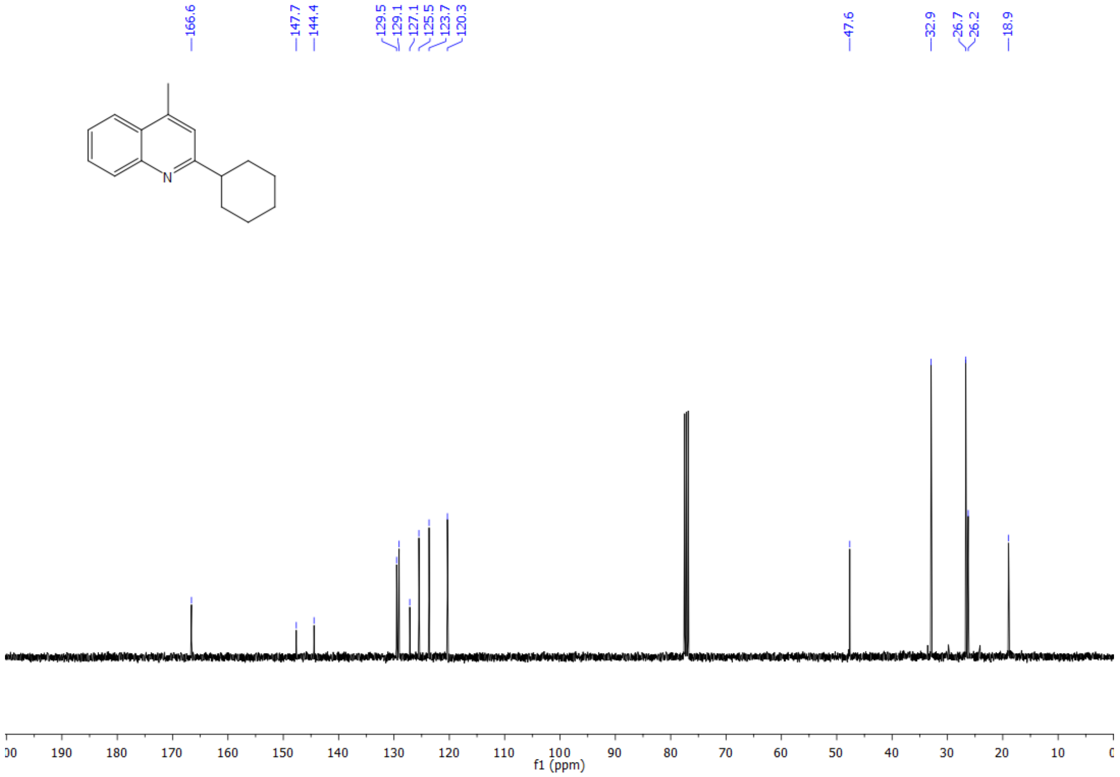

^{13}C NMR (101 MHz, $CDCl_3$) of **6k**

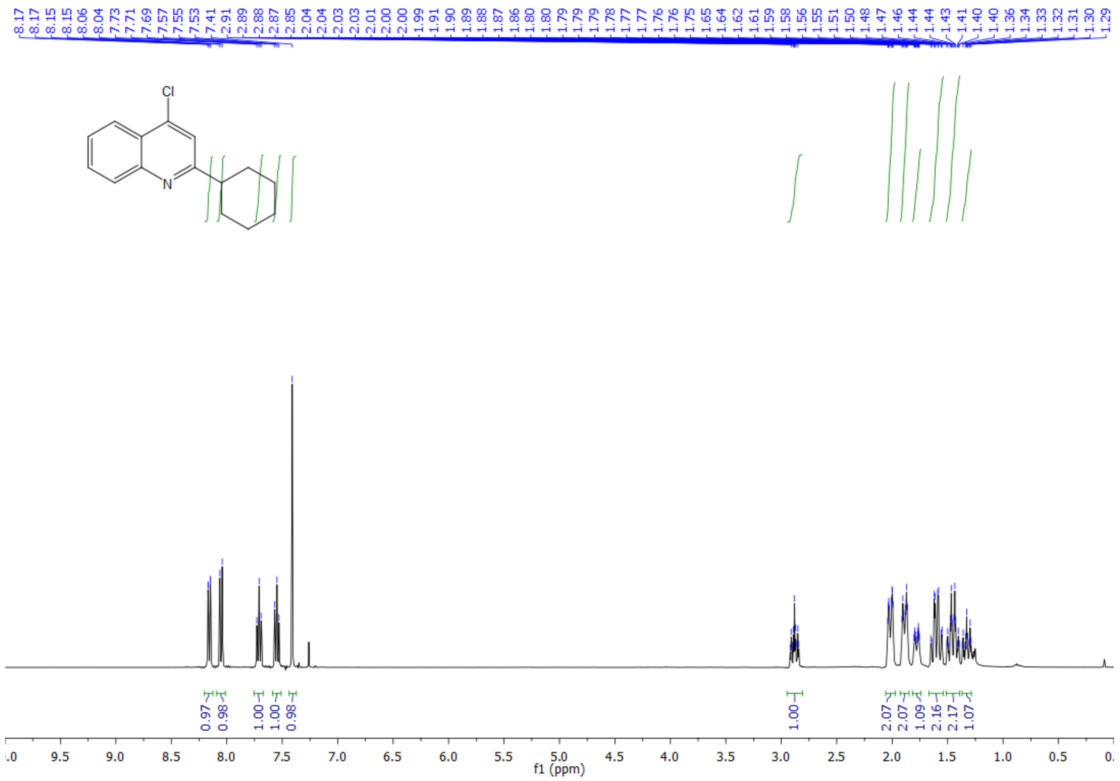

^{1}H NMR (400 MHz, CDCl$_3$) of **6l**

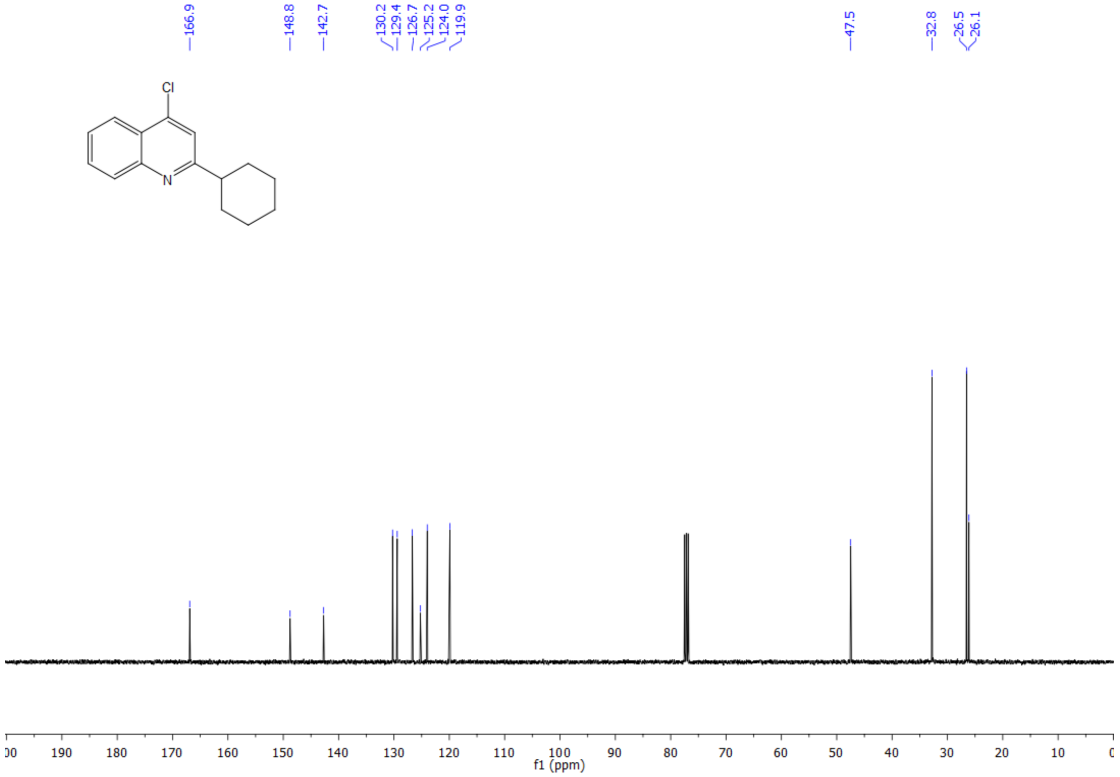

^{13}C NMR (101 MHz, CDCl$_3$) of **6l**

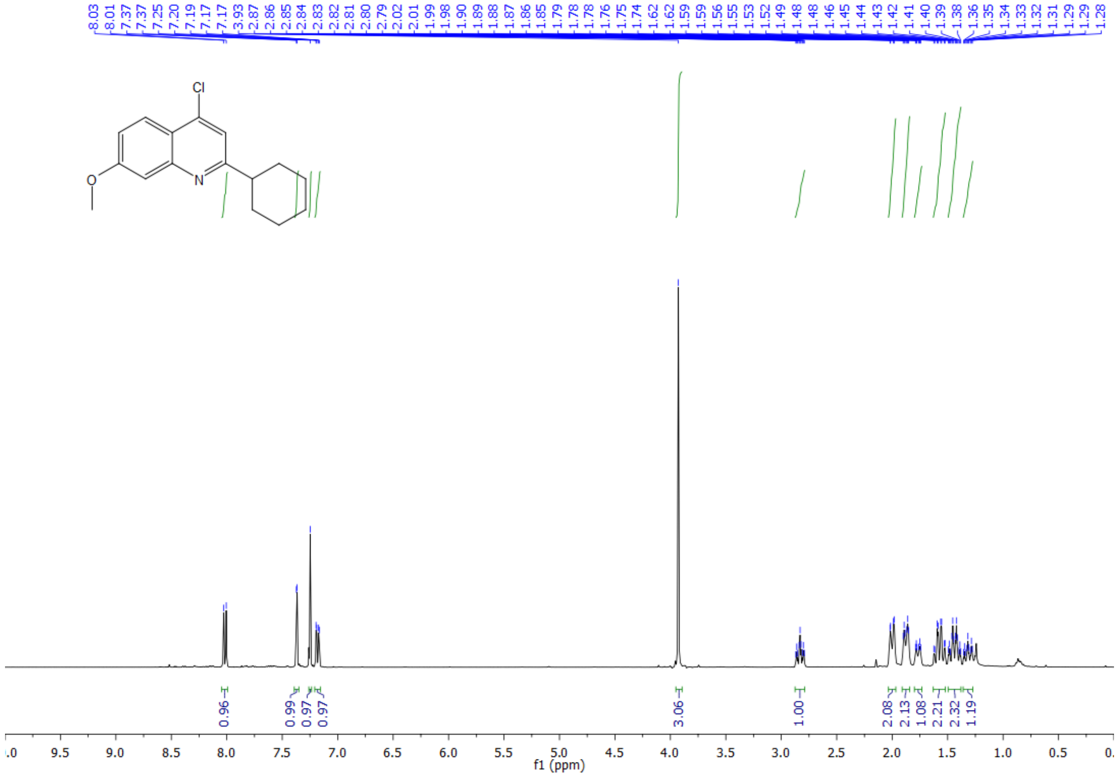

^{1}H NMR (400 MHz, $CDCl_3$) of **6m**

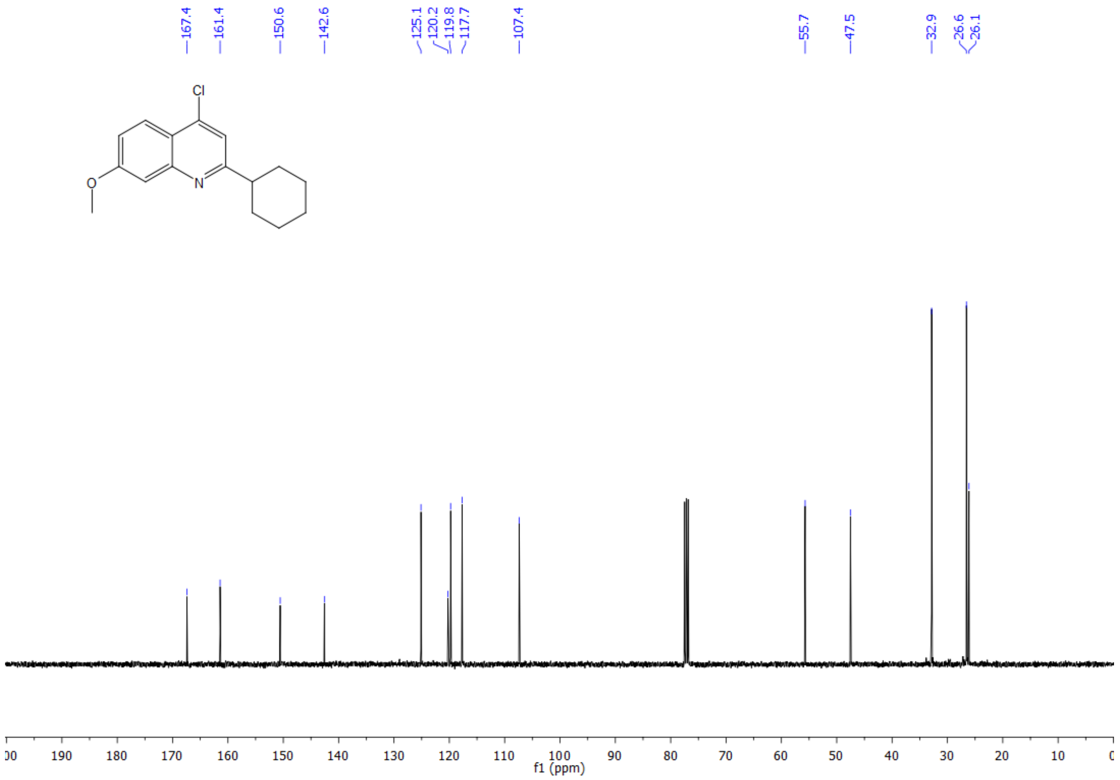

^{13}C NMR (101 MHz, $CDCl_3$) of **6m**

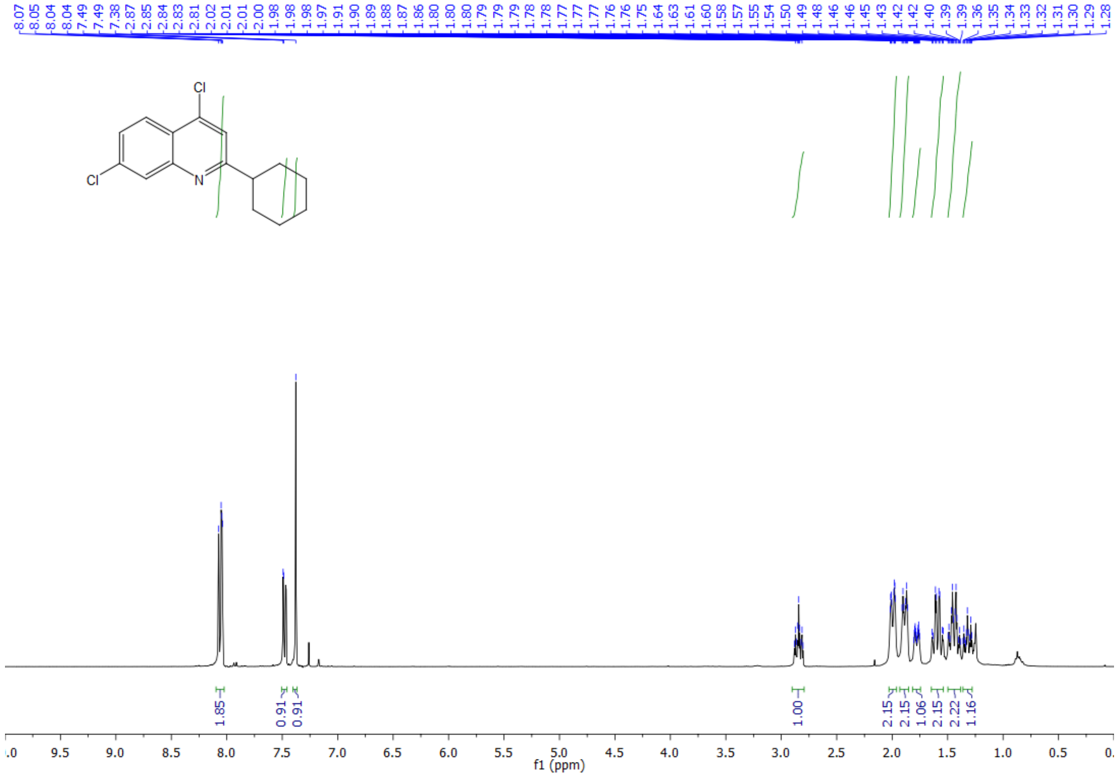

^{1}H NMR (400 MHz, $CDCl_3$) of **6n**

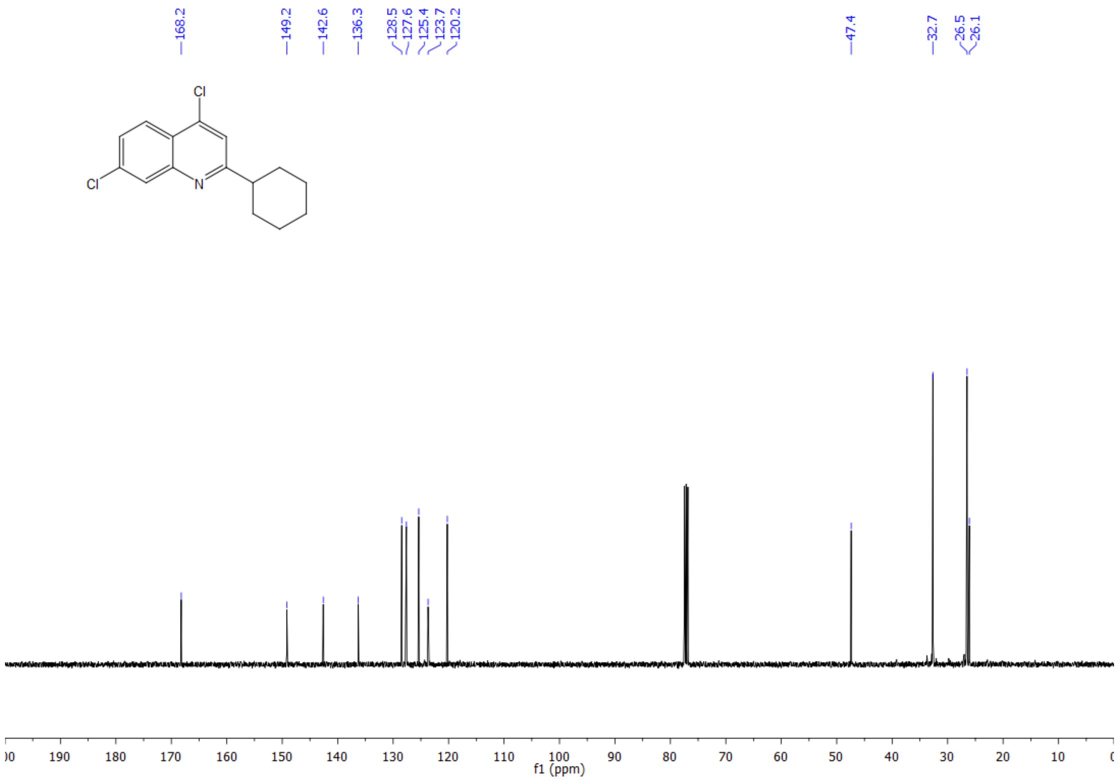

^{13}C NMR (101 MHz, $CDCl_3$) of **6n**

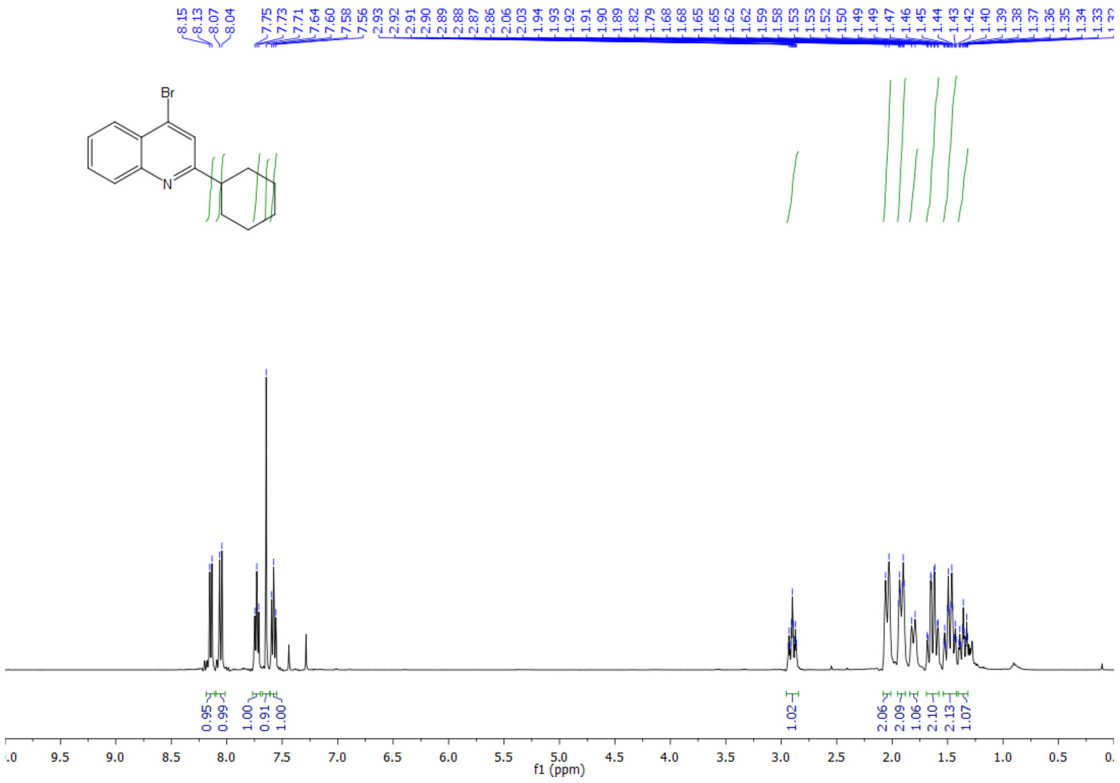

^1H NMR (400 MHz, $CDCl_3$) of **6o**

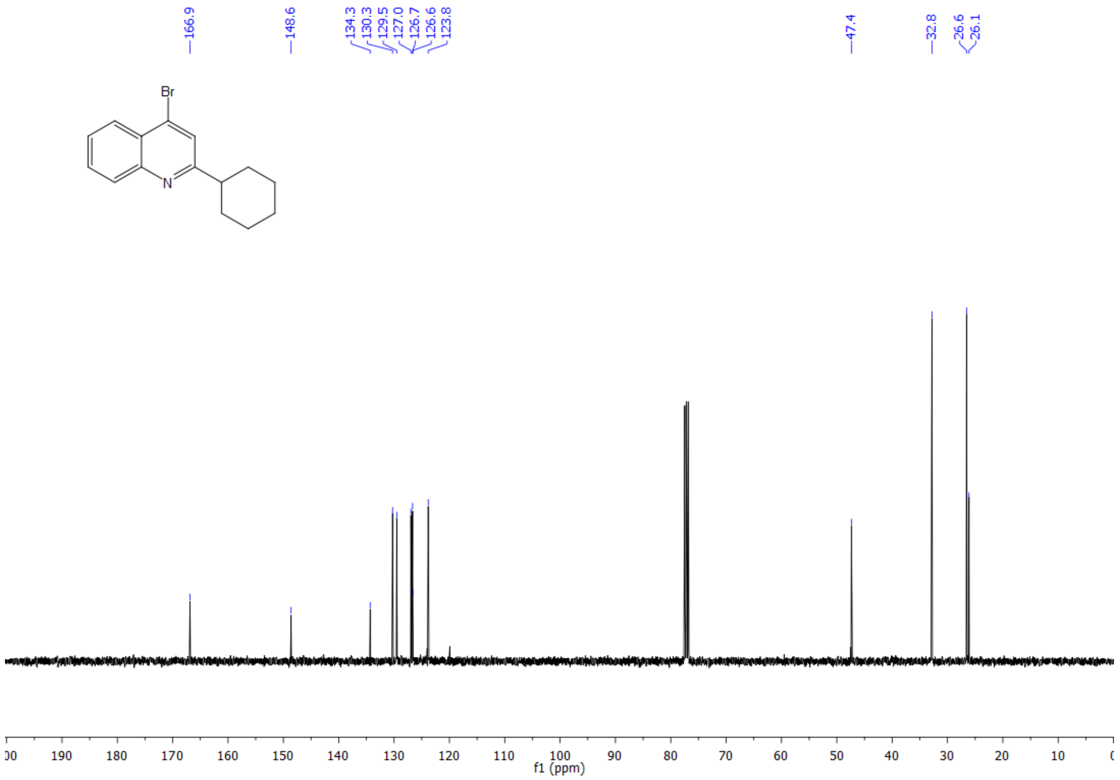

^{13}C NMR (101 MHz, $CDCl_3$) of **6o**

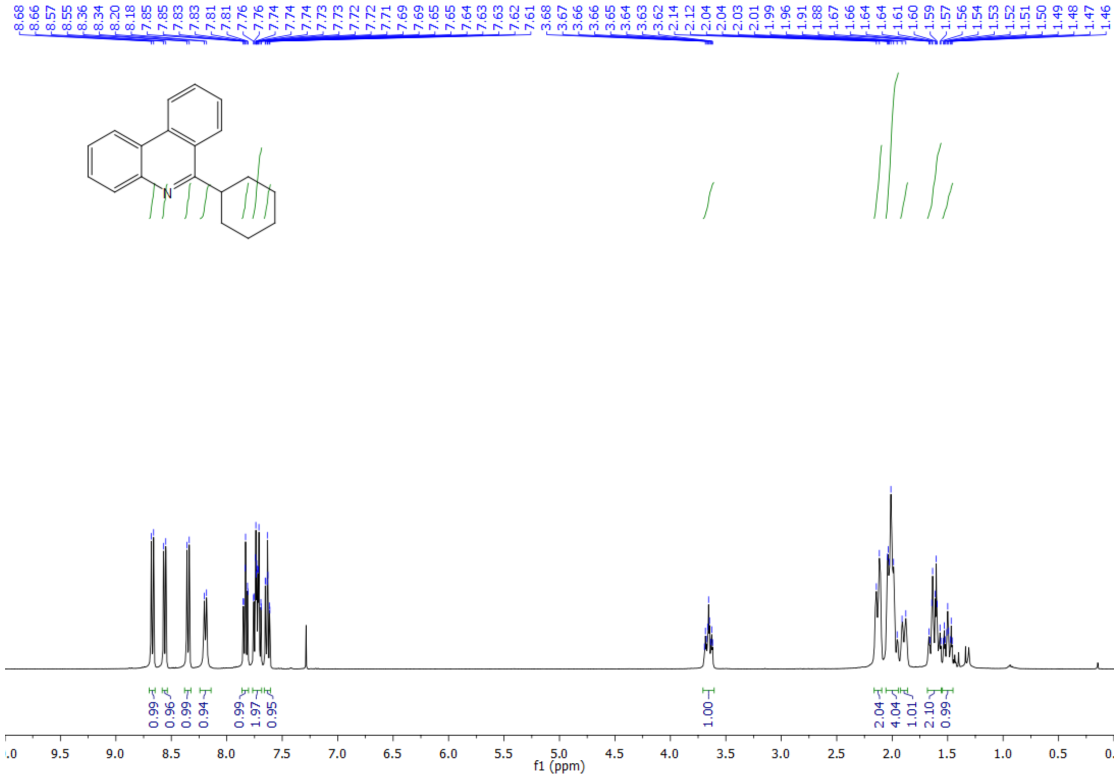

^{1}H NMR (400 MHz, $CDCl_3$) of **6p**

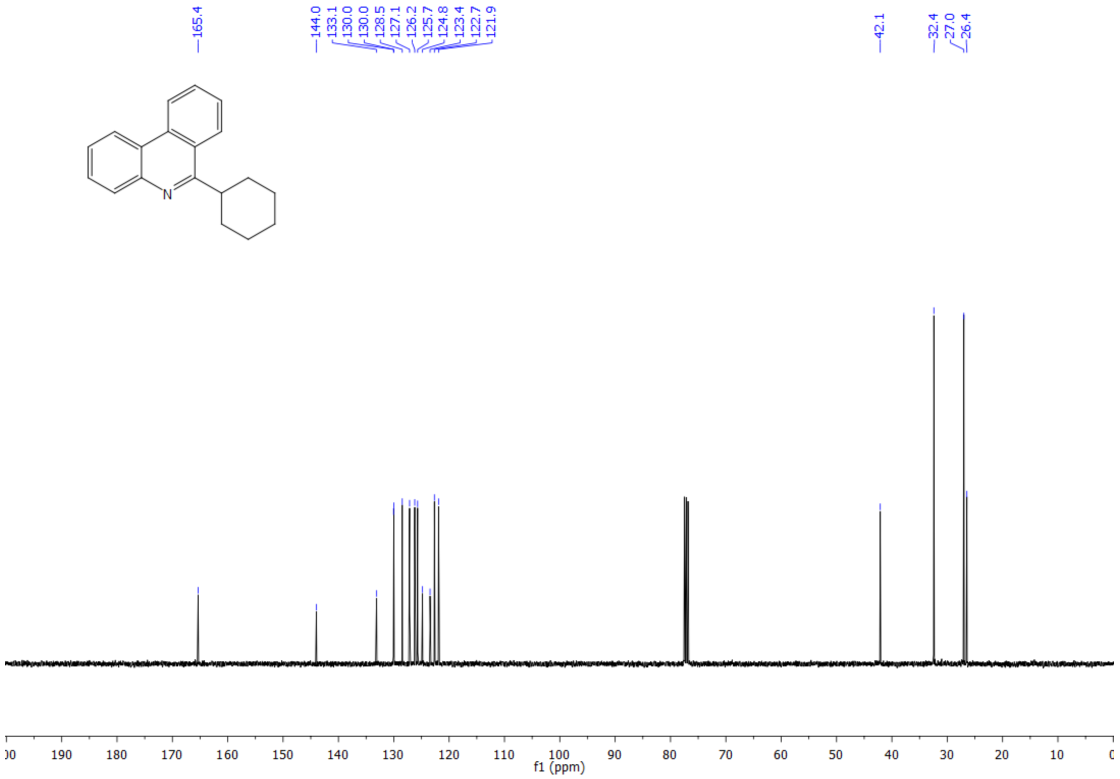

^{13}C NMR (101 MHz, $CDCl_3$) of **6p**

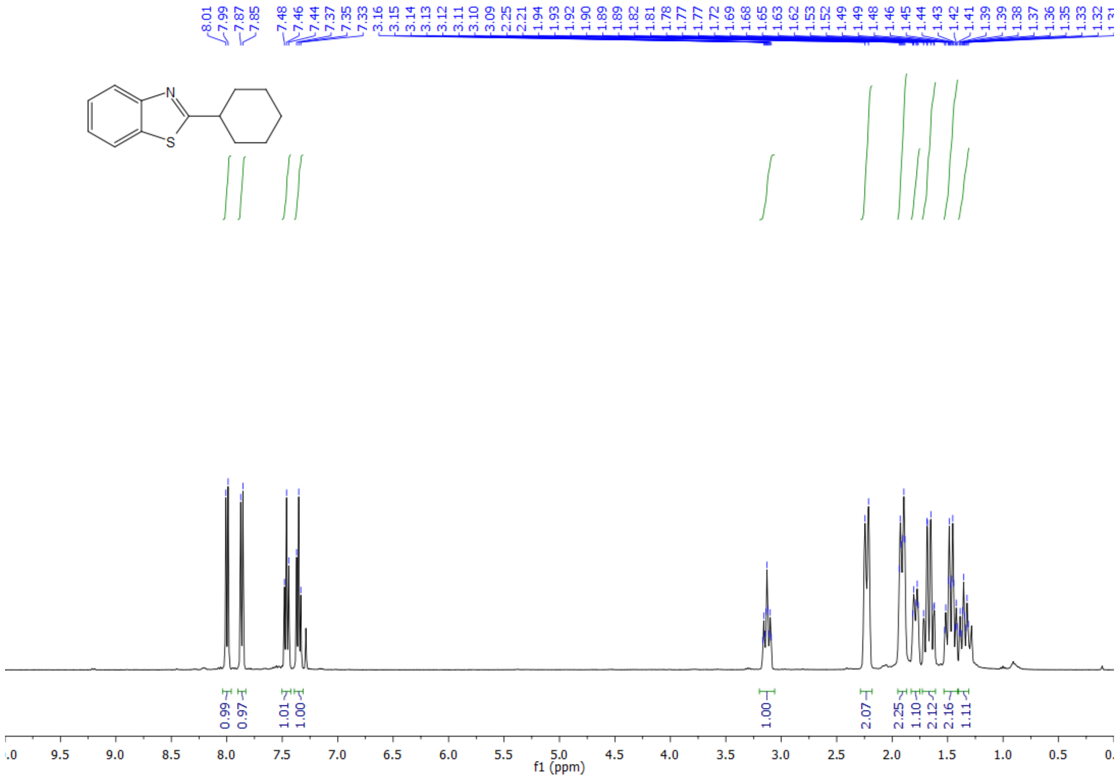

^{1}H NMR (400 MHz, $CDCl_3$) of **6q**

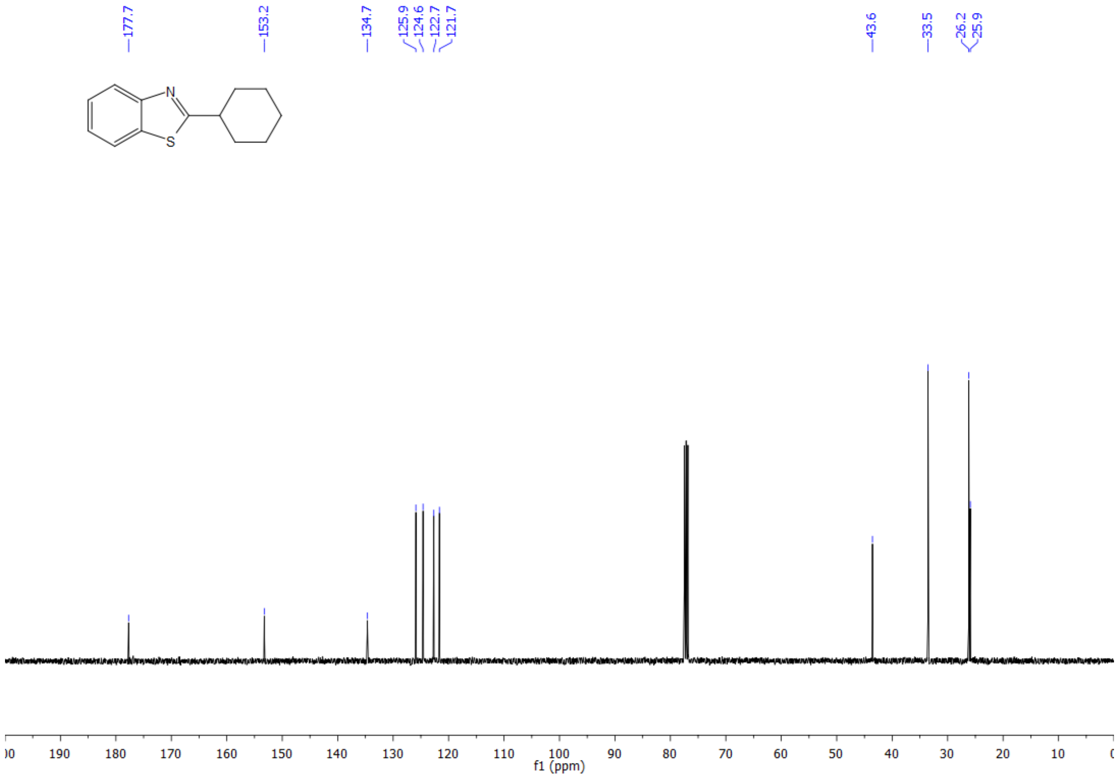

^{13}C NMR (101 MHz, $CDCl_3$) of **6q**

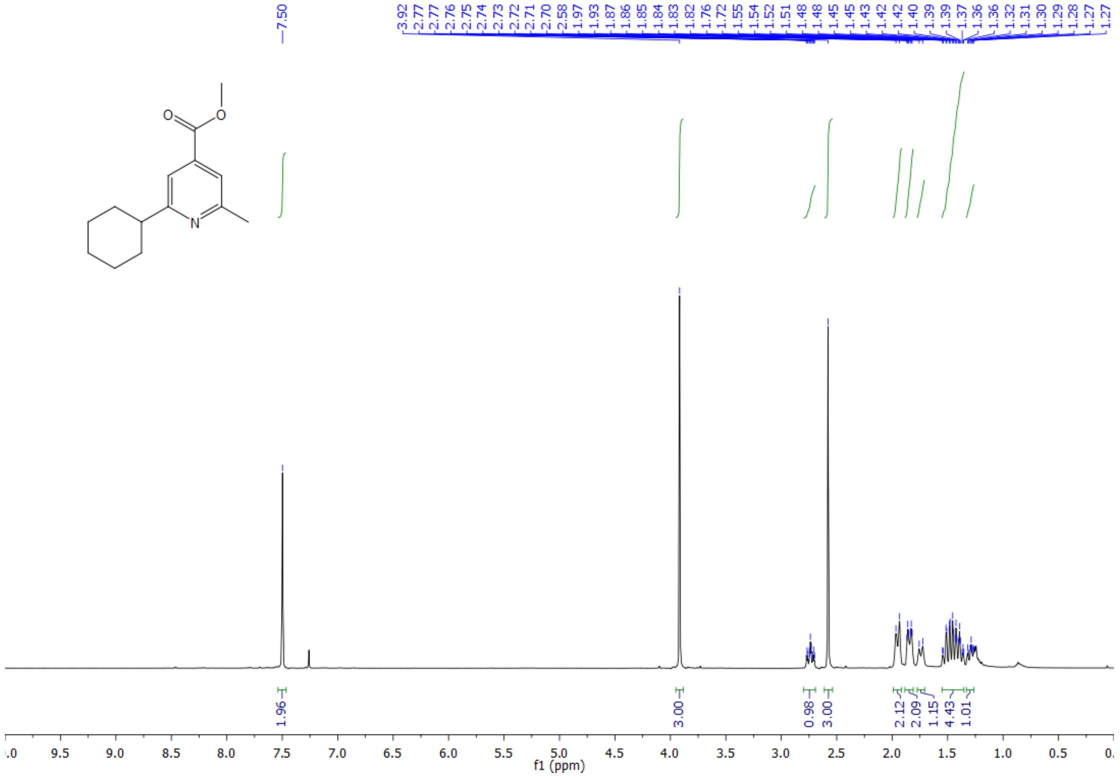

^{1}H NMR (400 MHz, CDCl$_3$) of **6r**

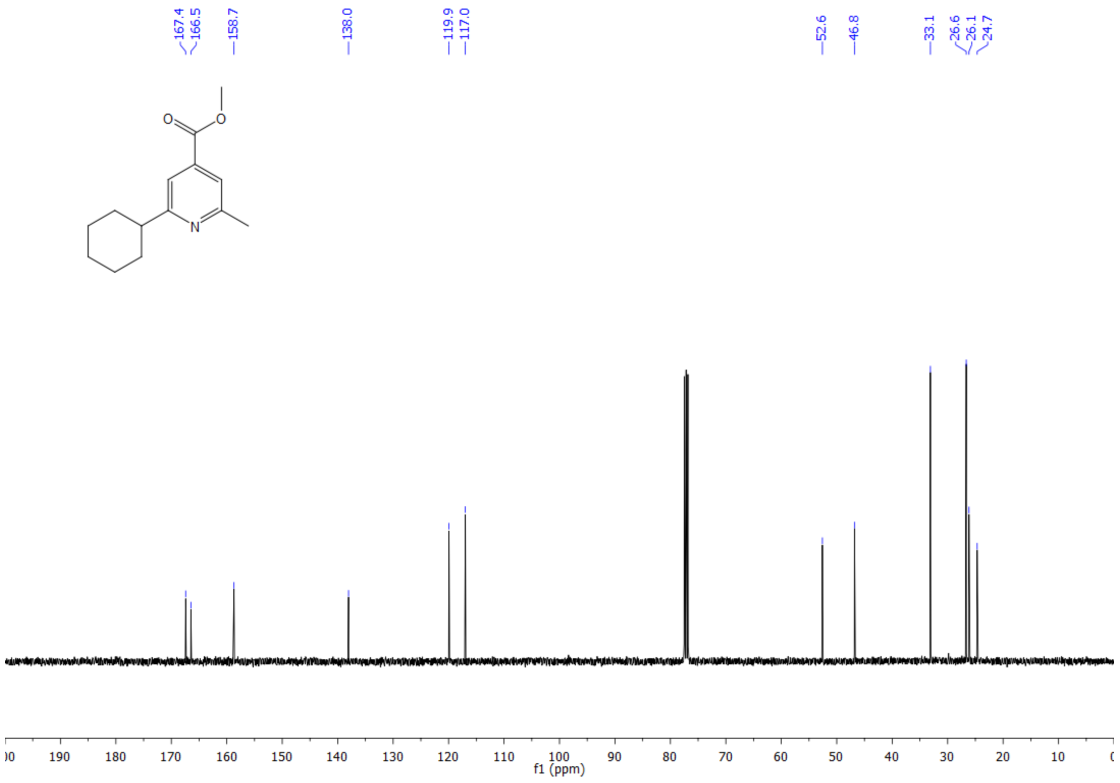

^{13}C NMR (101 MHz, CDCl$_3$) of **6r**

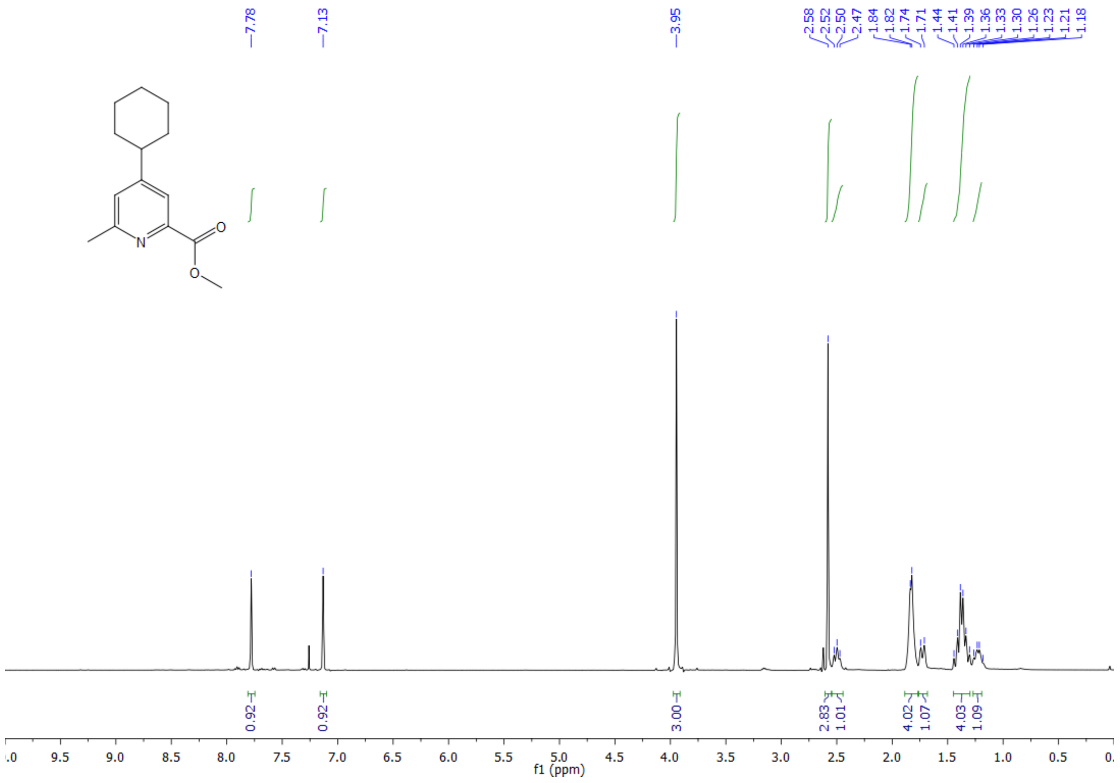

^{1}H NMR (400 MHz, $CDCl_3$) of **6s**

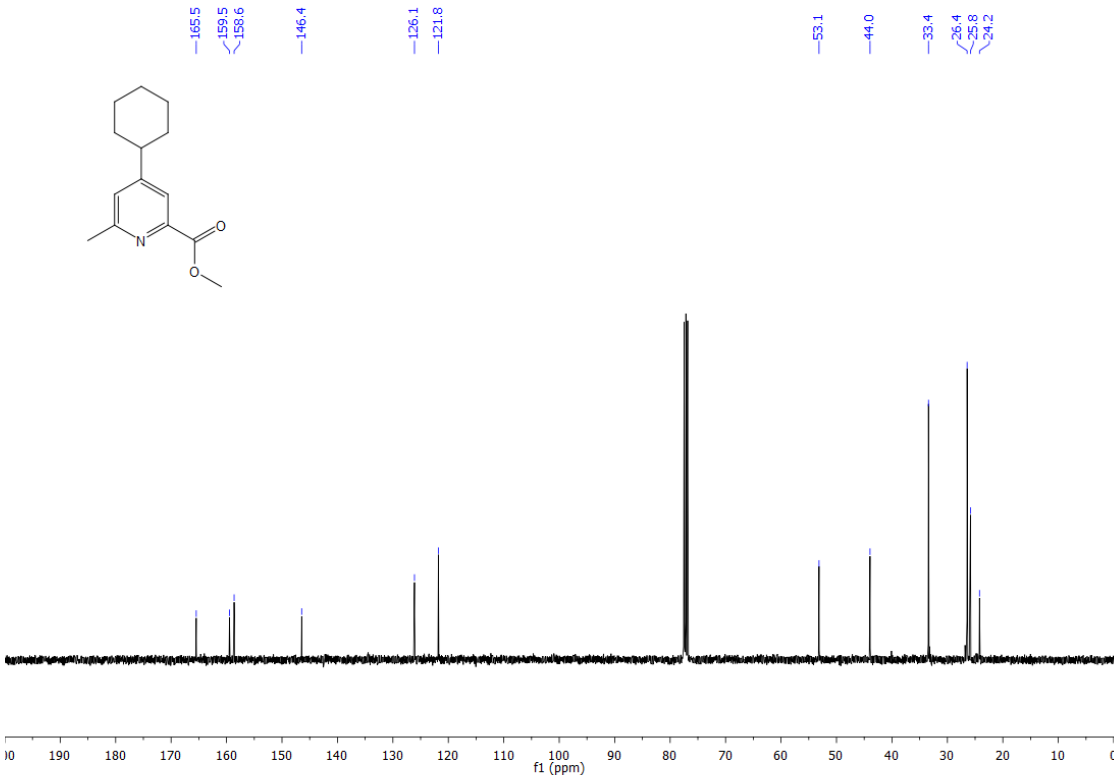

^{13}C NMR (101 MHz, $CDCl_3$) of **6s**

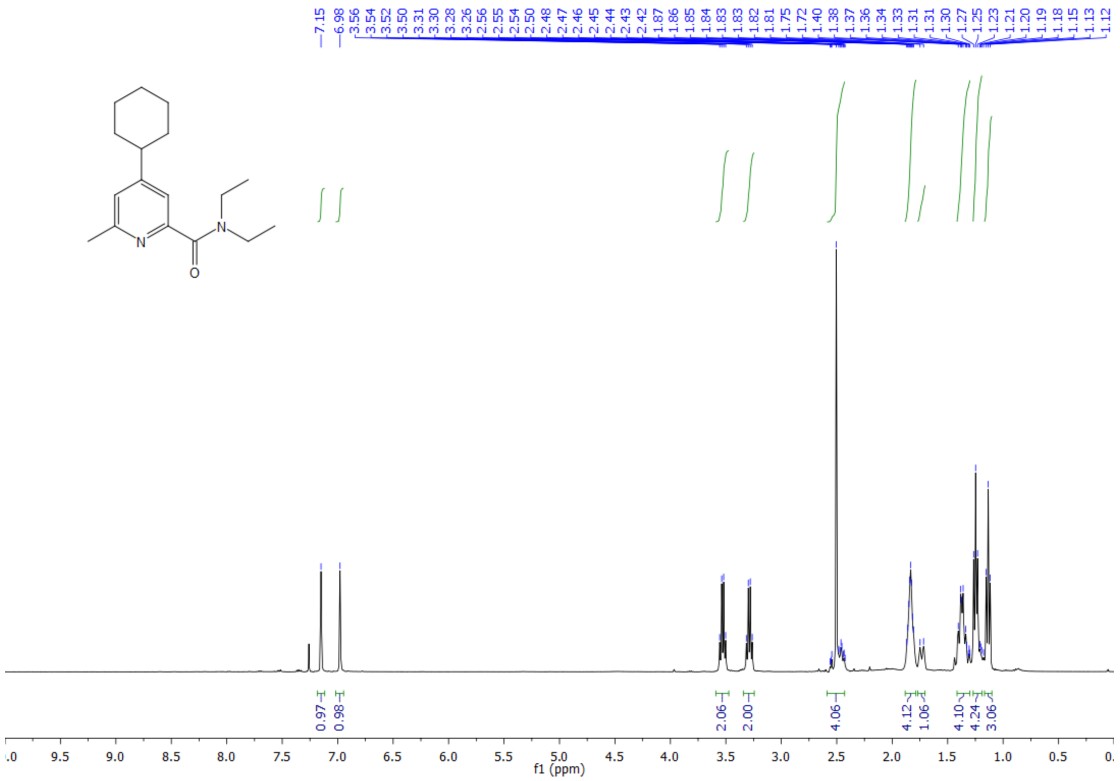

^{1}H NMR (400 MHz, $CDCl_3$) of **6t**

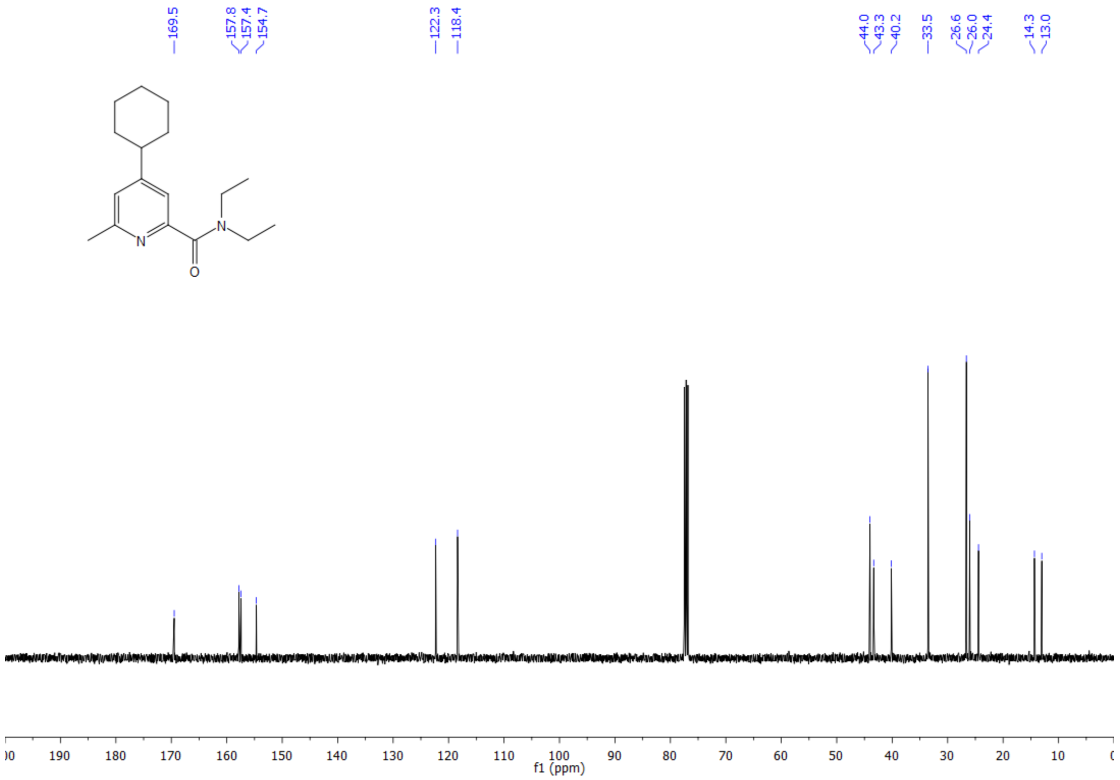

^{13}C NMR (101 MHz, $CDCl_3$) of **6t**

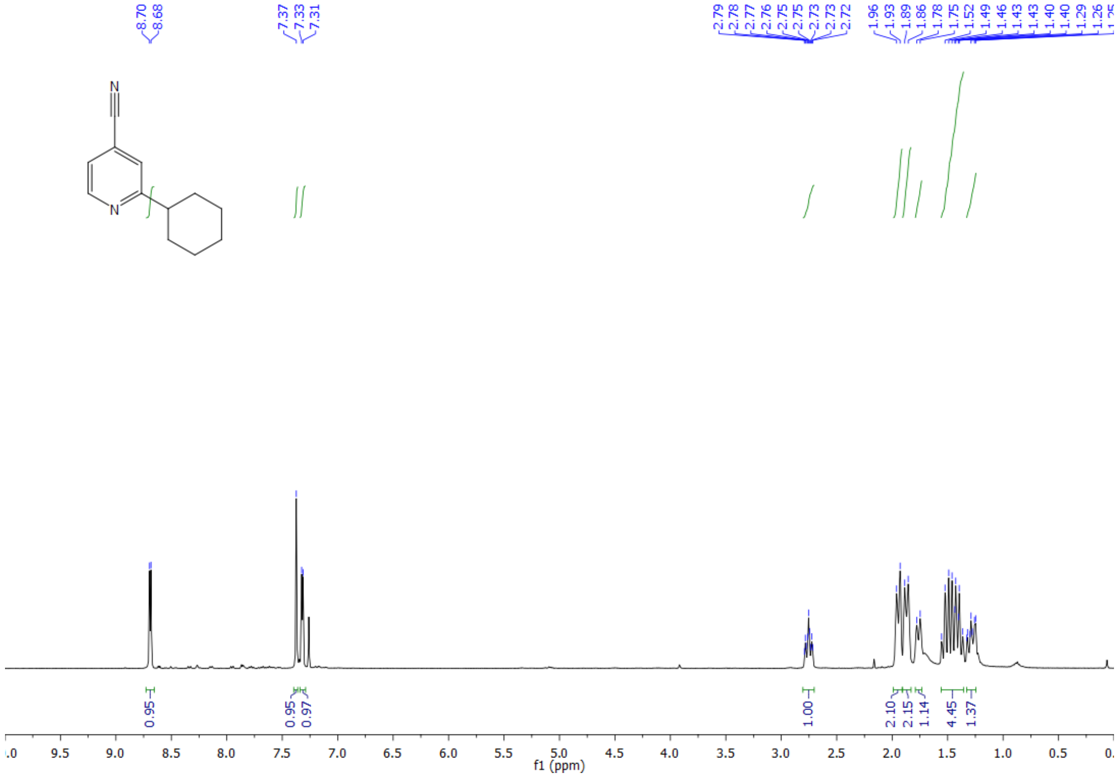

^{1}H NMR (400 MHz, $CDCl_3$) of **6u**

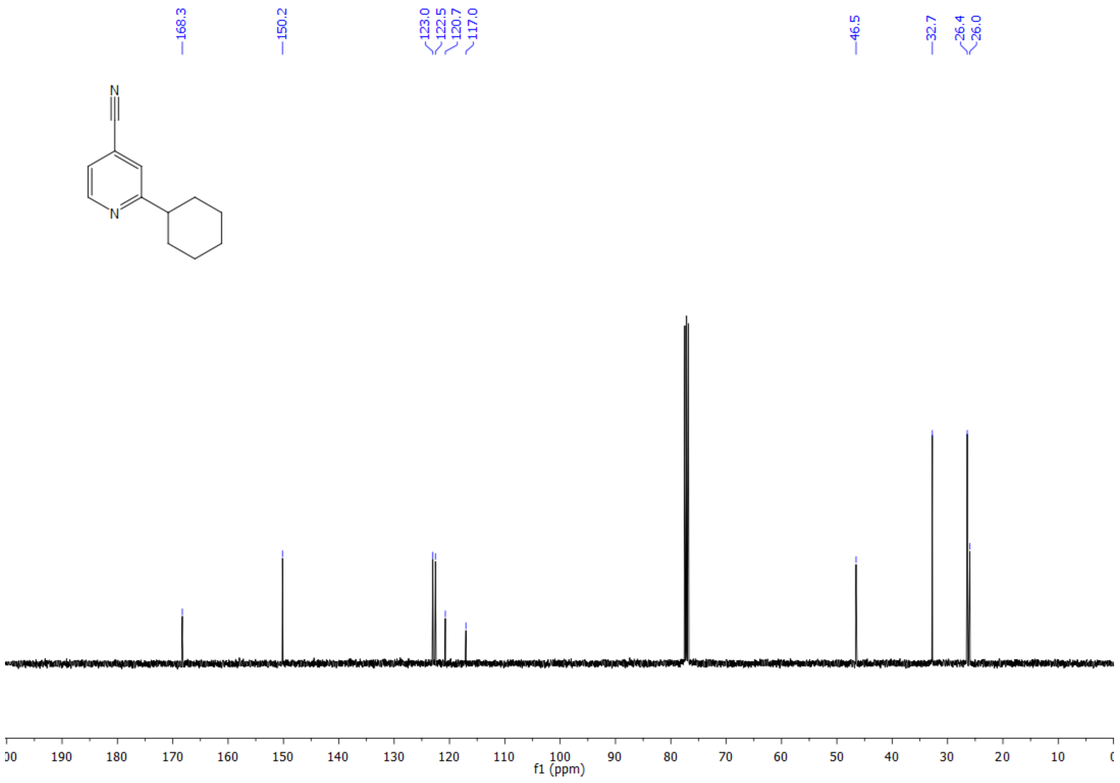

^{13}C NMR (101 MHz, $CDCl_3$) of **6u**

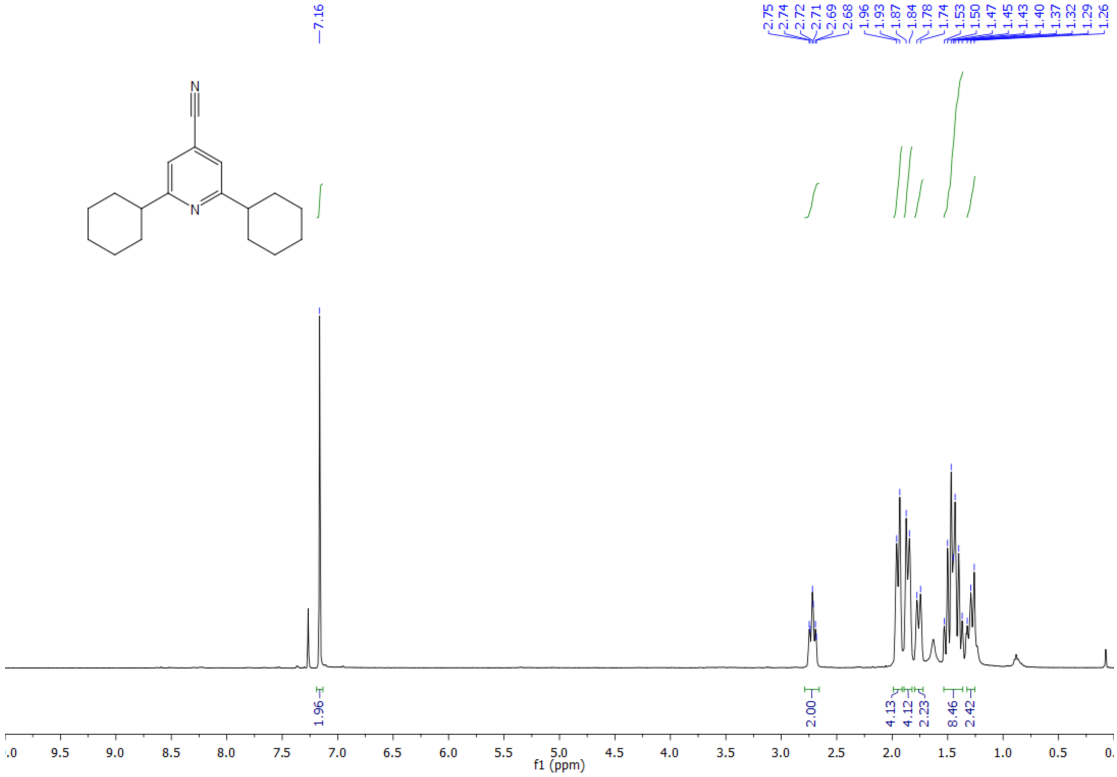

^{1}H NMR (400 MHz, $CDCl_3$) of **6u**

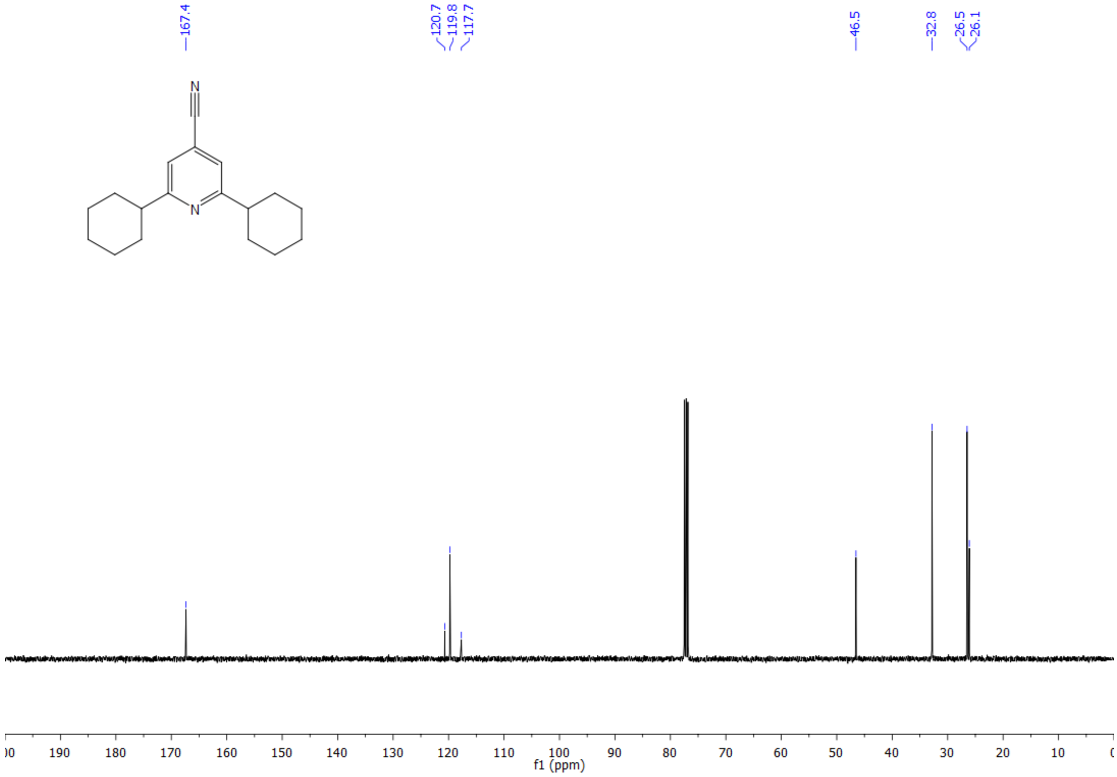

^{13}C NMR (101 MHz, $CDCl_3$) of **6u'**

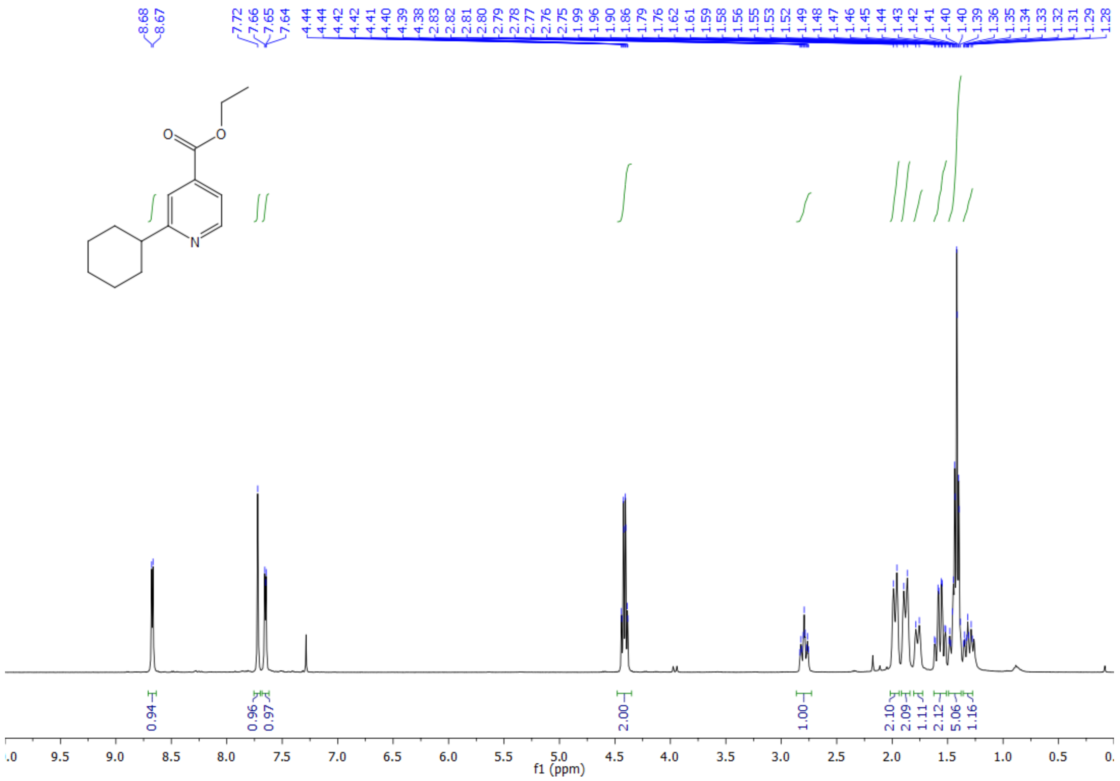

^{1}H NMR (400 MHz, $CDCl_3$) of **6v**

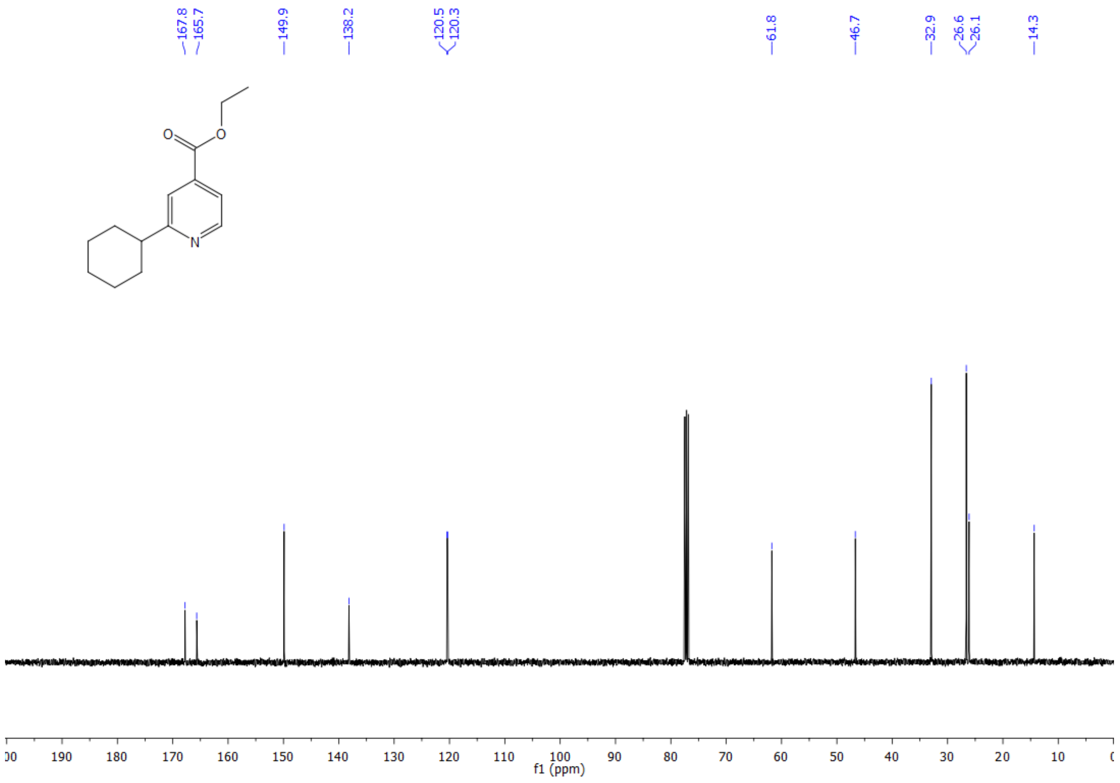

^{13}C NMR (101 MHz, $CDCl_3$) of **6v**

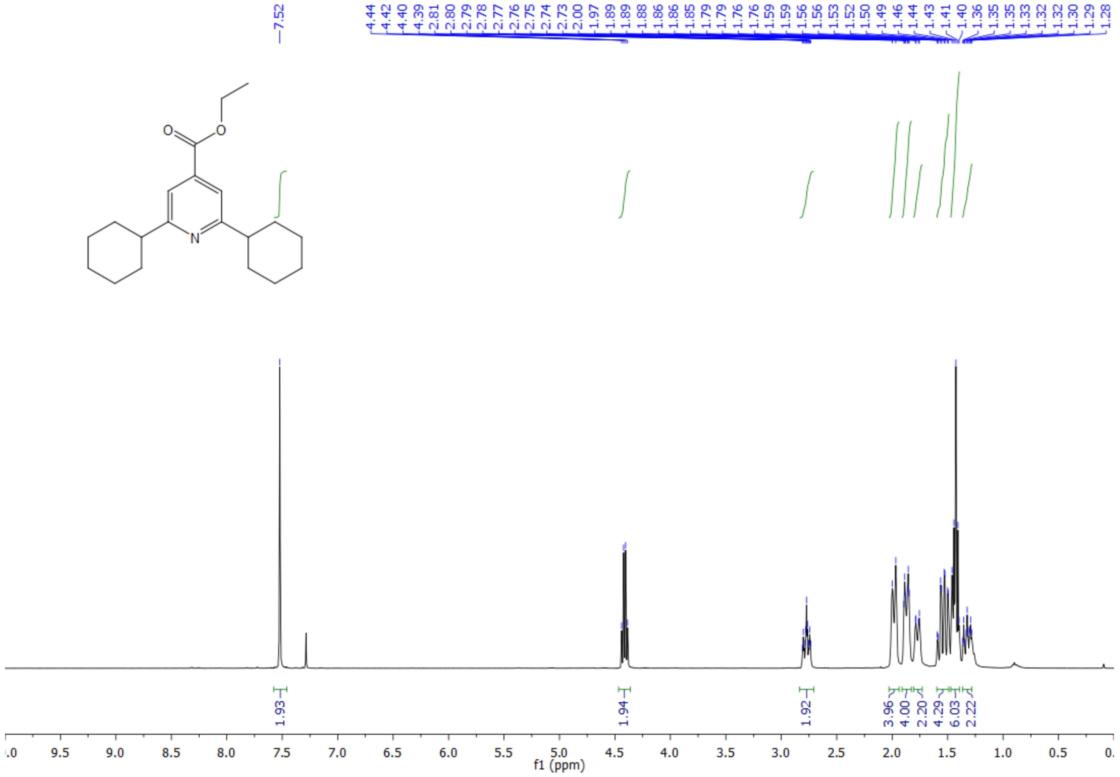

^{1}H NMR (400 MHz, CDCl$_3$) of **6v'**

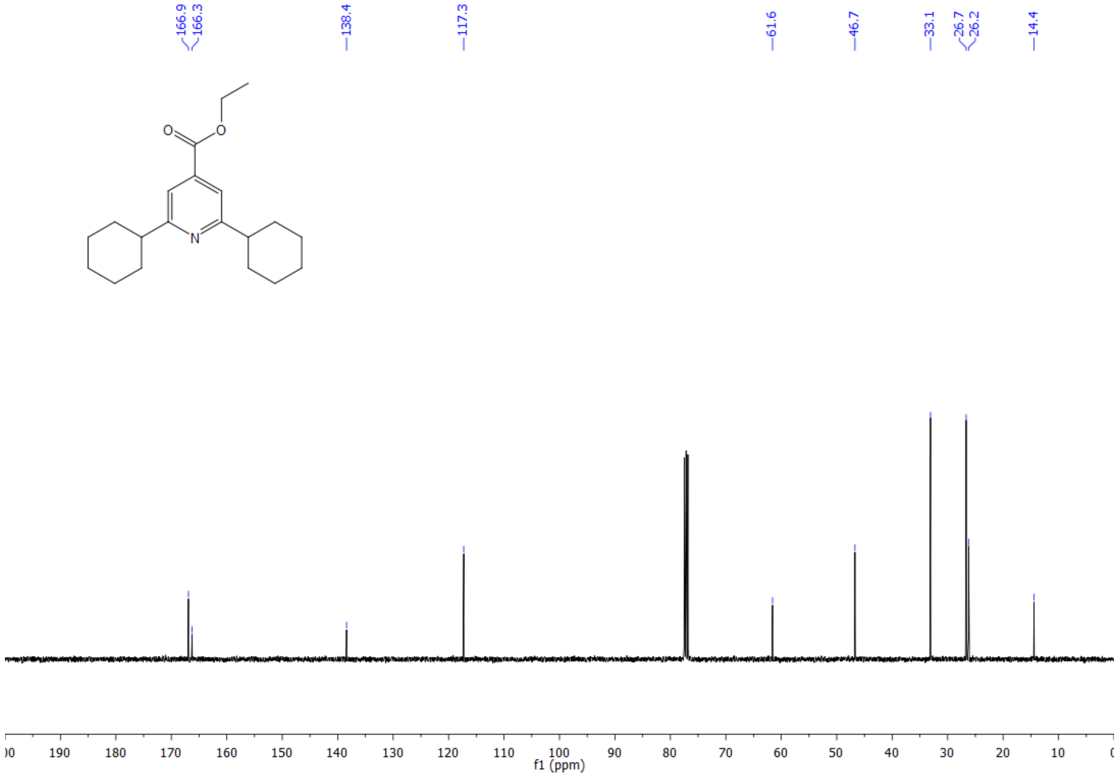

^{13}C NMR (101 MHz, CDCl$_3$) of **6v'**

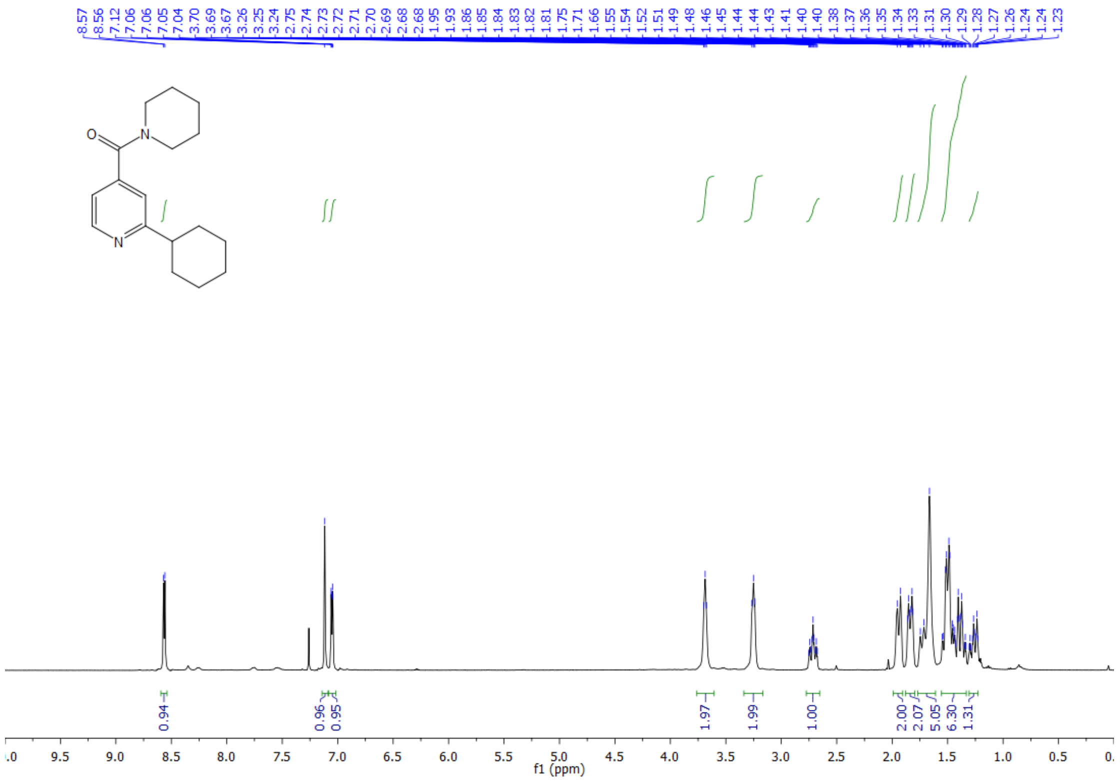

^{1}H NMR (400 MHz, $CDCl_3$) of **6w**

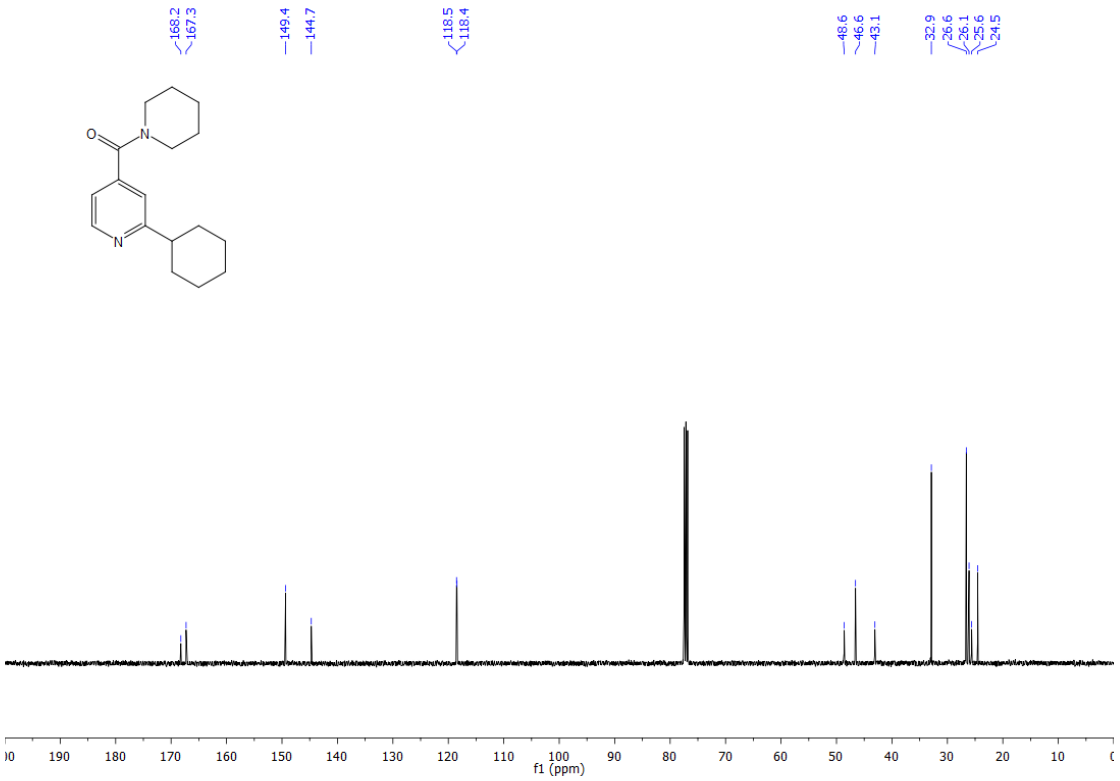

^{13}C NMR (101 MHz, $CDCl_3$) of **6w**

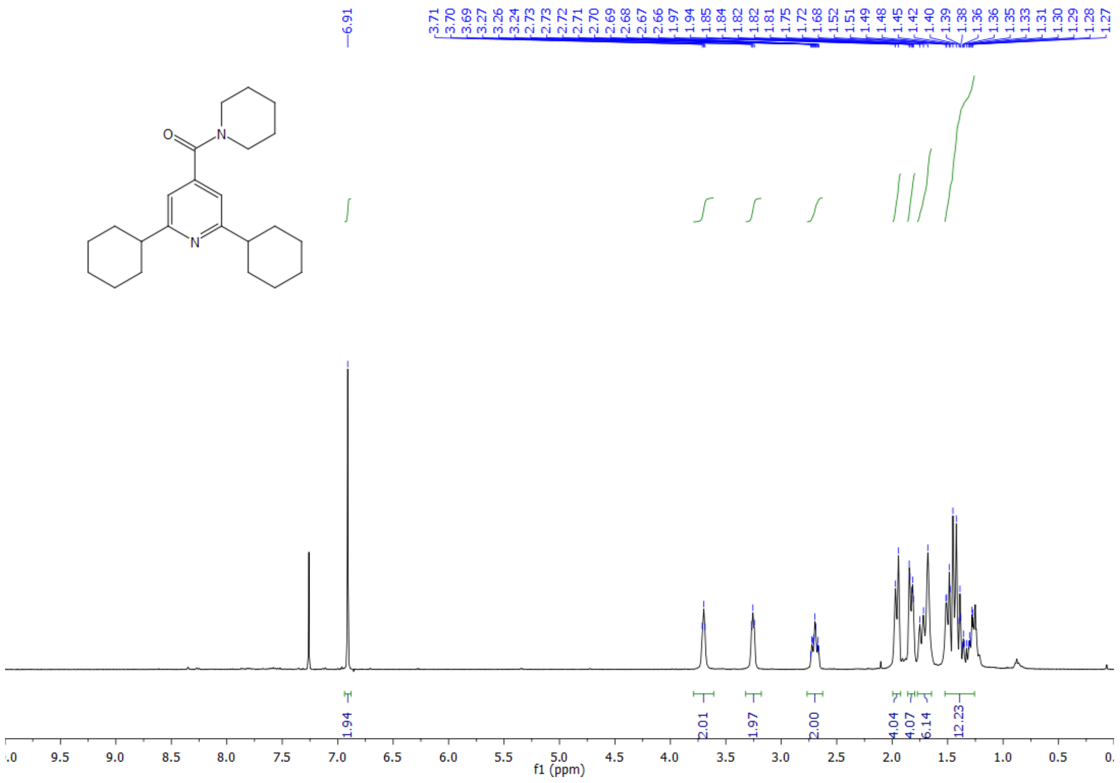

^{1}H NMR (400 MHz, $CDCl_3$) of **6w'**

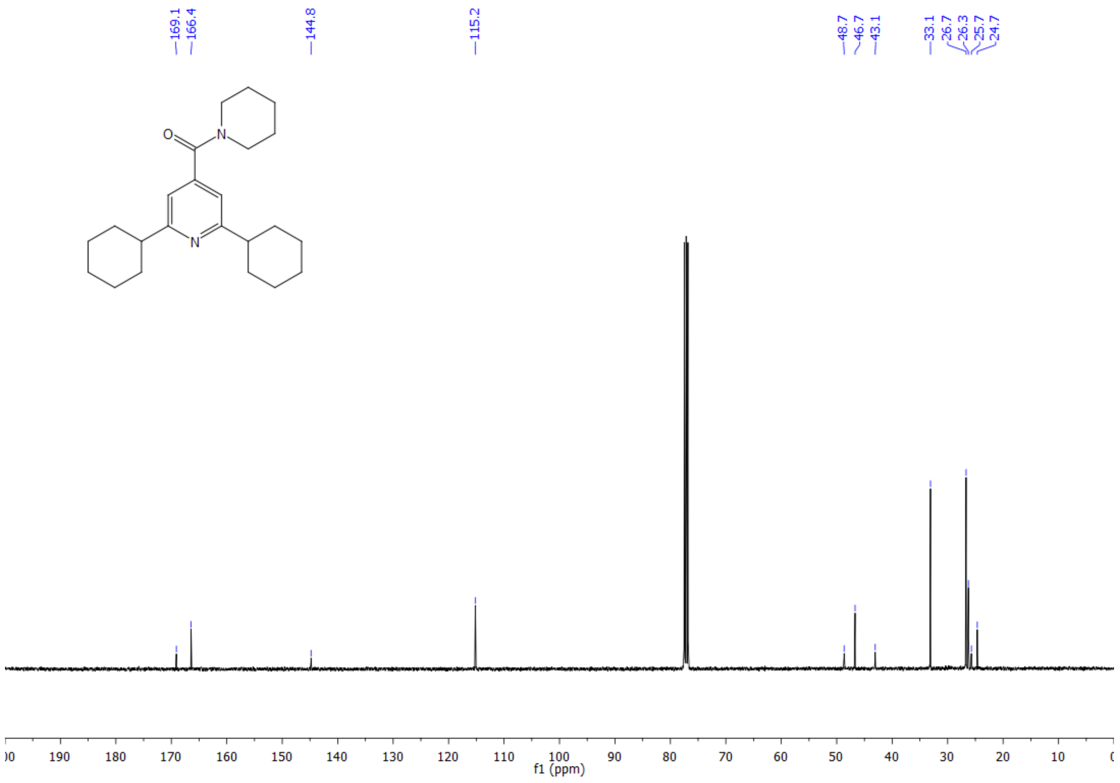

^{13}C NMR (101 MHz, $CDCl_3$) of **6w'**

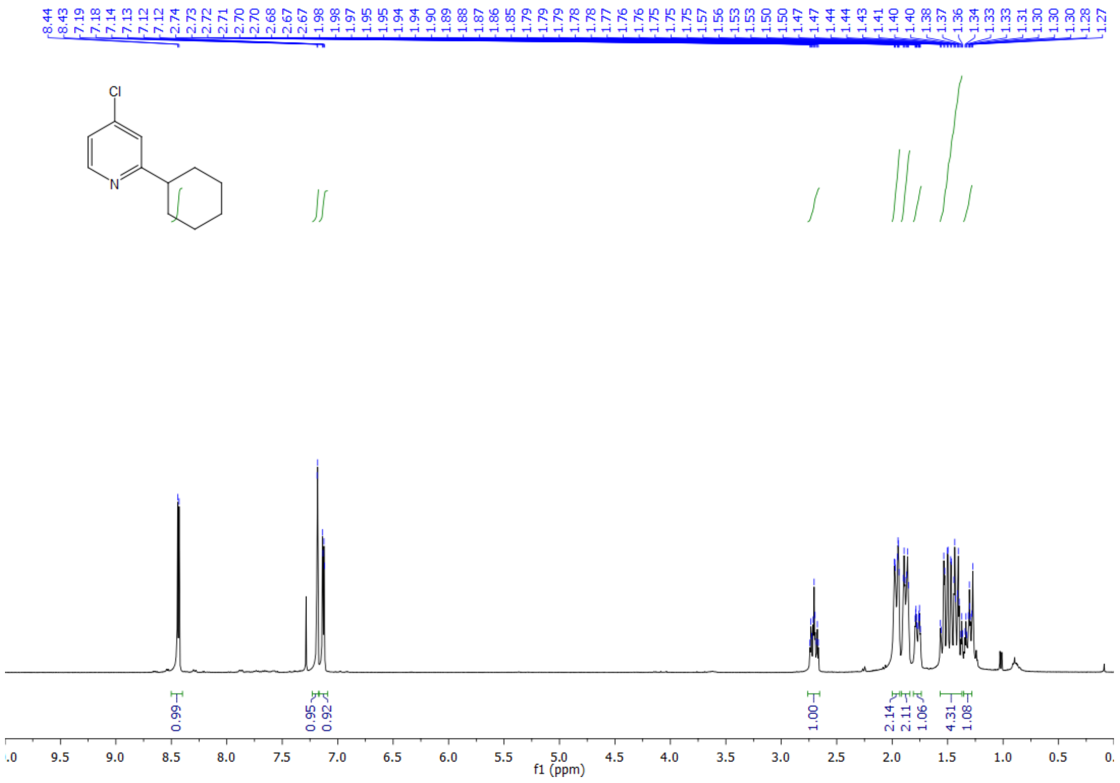

^{1}H NMR (400 MHz, $CDCl_3$) of **6x**

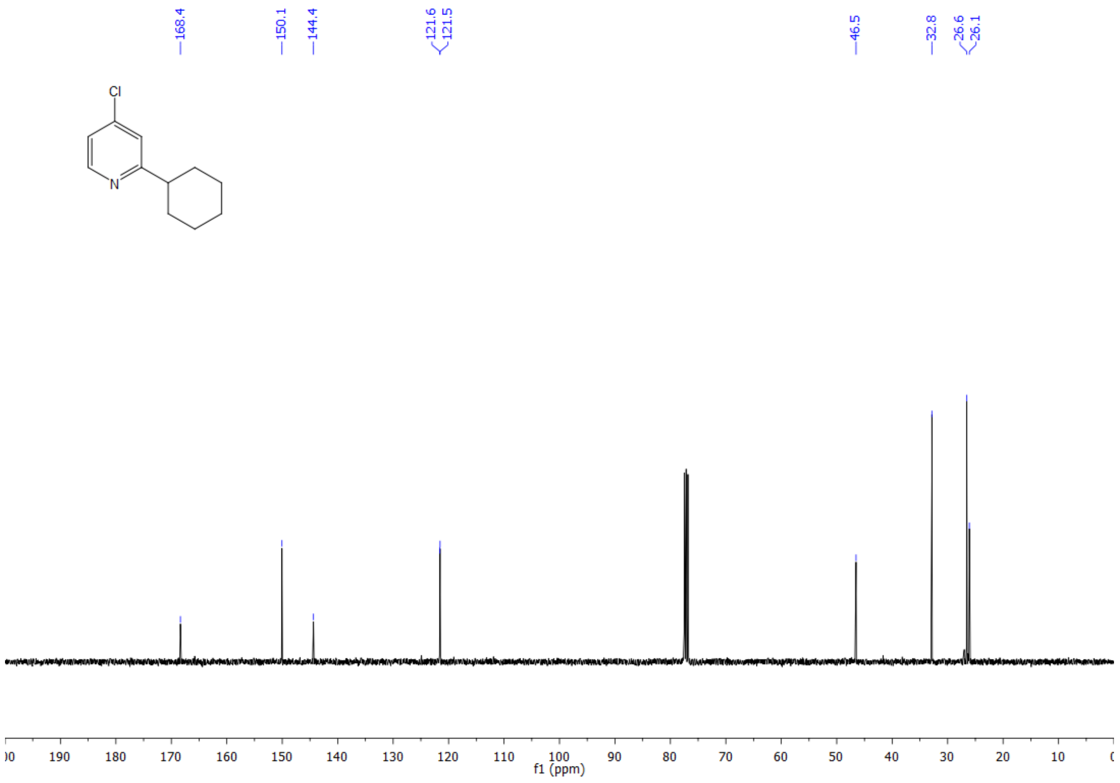

^{13}C NMR (101 MHz, $CDCl_3$) of **6x**

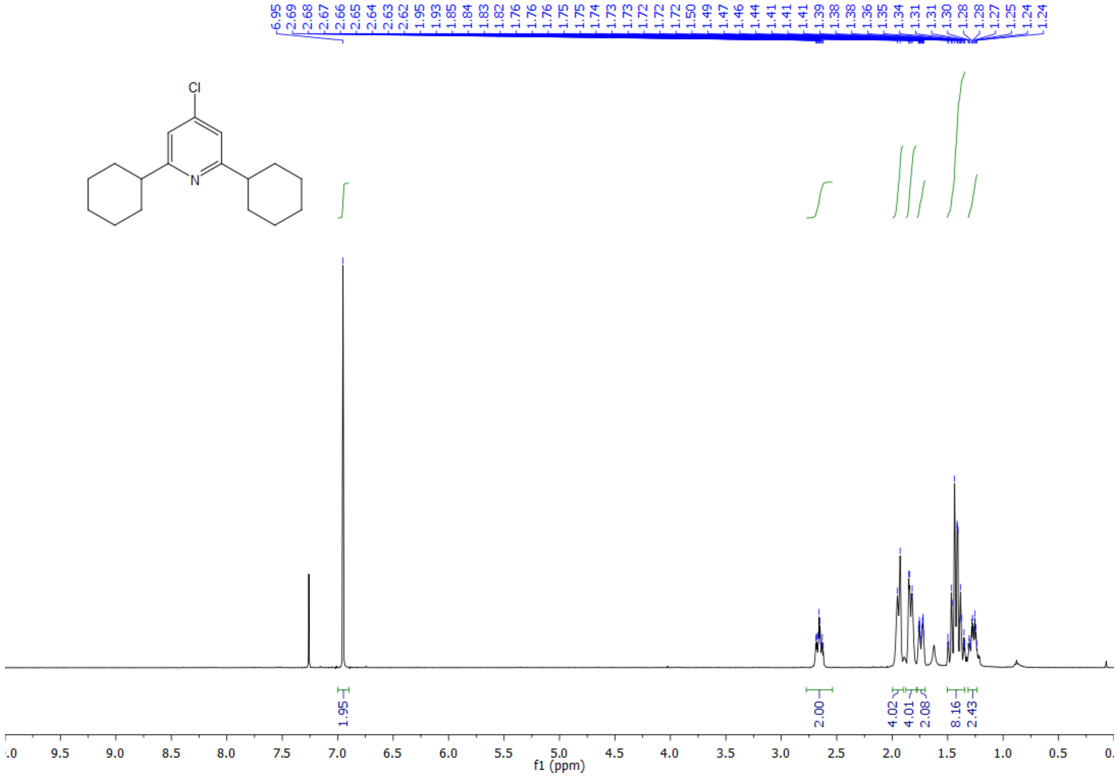

^{1}H NMR (400 MHz, $CDCl_3$) of **6x’**

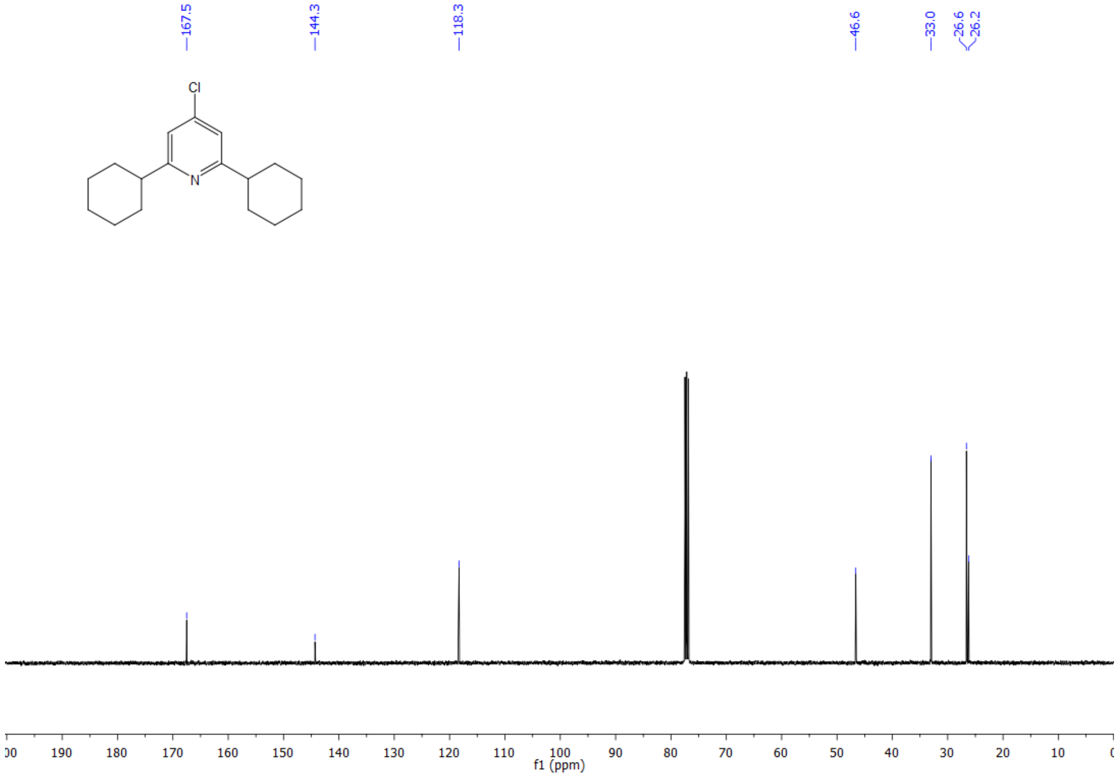

^{13}C NMR (101 MHz, $CDCl_3$) of **6x’**
